# Post-functionalization of triamino-phenazinium dyes to reach near-infrared emission†

**DOI:** 10.1039/d4ra03245d

**Published:** 2024-06-17

**Authors:** Tatiana Munteanu, Jean-François Longevial, Gabriel Canard, Denis Jacquemin, Simon Pascal, Olivier Siri

**Affiliations:** a Aix Marseille Univ., CNRS UMR 7325 Centre Interdisciplinaire de Nanoscience de Marseille (CINaM) Campus de Luminy 13288 Marseille cedex 09 France simon.pascal@cnrs.fr olivier.siri@univ-amu.fr; b Université de Lorraine, LCP-A2MC F-57000 Metz France; c Nantes Université, CEISAM UMR 6230, CNRS Nantes F-44000 France; d Institut Universitaire de France (IUF) Paris France

## Abstract

This study presents the synthesis and characterization of phenazinium dyes with absorption ranging from red to far-red, as well as emission extending into the far-red to near-infrared (NIR) region. The procedure involves the post-functionalization of a triamino-phenazinium that was recently reported as a theranostic agent. The introduction of electron-withdrawing moieties is accomplished through acylation or aromatic nucleophilic substitution. For one of the obtained products, a further substitution step could be achieved with primary amines to tune the electron density of the phenazinium core. The isolated dyes exhibit promising features that hold potential for future applications as biological markers or therapeutic agents.

## Abstract 2

We showed the possibility of redshifting the absorption and emission properties of triamino-phenazinium fluorophores by a one step post-synthesis strategy, involving the introduction of an electron-withdrawing group.

## Graphical abstract

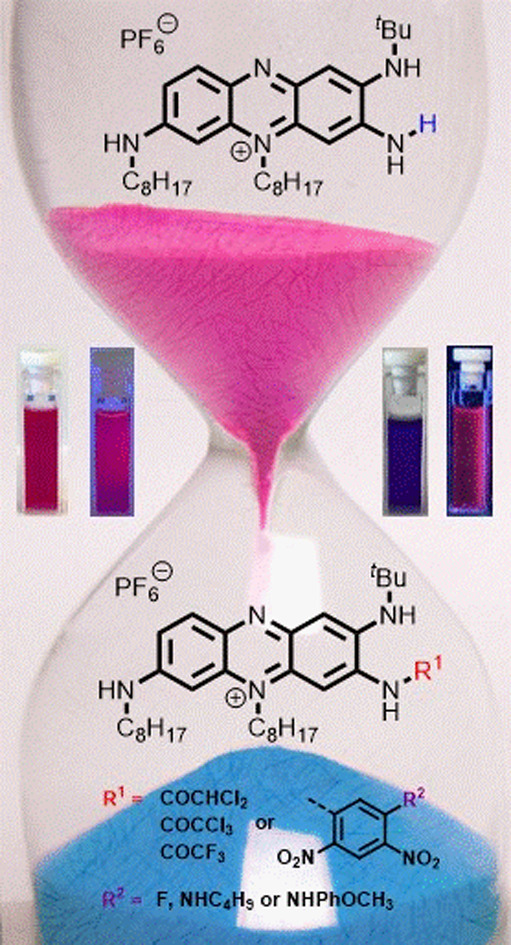

## Introduction

Following the serendipitous discovery of Mauveine by Perkin in 1856,^1^ phenazine dyes have known a period of industrial blossoming, marking the transition from natural pigments to synthetic dyes due to advantageous scalable fabrication, faster dying processes, deep coloration of various substrates and high photostability.^2–4^ Nowadays, the appealing features of phenazines and their cationic analogues, phenaziniums, have attracted the interest of researchers in a wide range of applications including the biomedical field. Indeed, this family of chromophores is displaying cytotoxicity through radical oxygen species generation, pH sensitivity, high Stokes shift and tunable photophysical properties, which are sought for optical bio-imaging.^5,6^

**Fig. 1:**
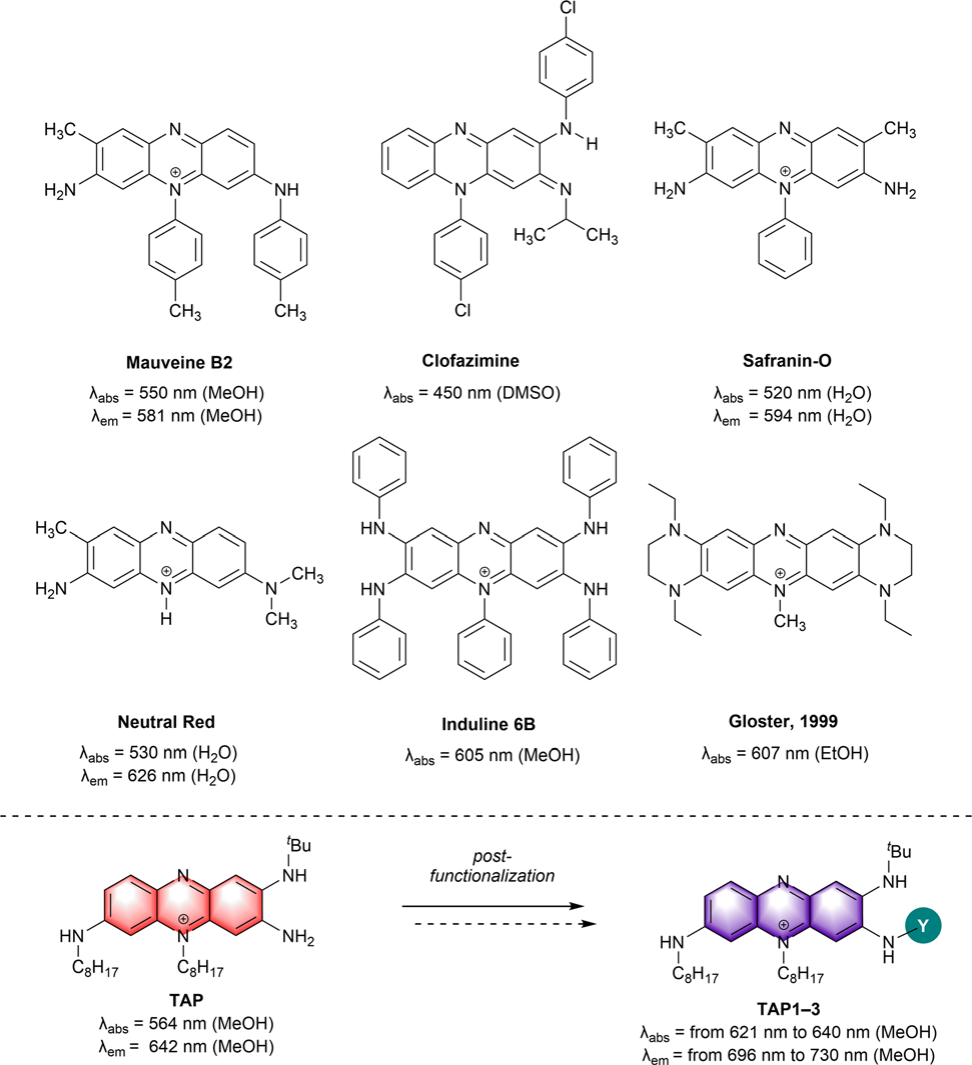
Representative examples of amino-phenazine dyes (top) and amino-phenaziniums reported herein (bottom). Counterions omitted for clarity.

Amino-phenazine and amino-phenazinium derivatives clearly stand out in terms of synthesis versatility and superior photophysical properties. Indeed, the amino moieties notably increase the electron density on the phenazine aromatic system and improve the fluorescence efficiency by suppressing non-radiative relaxation processes.^7^ Classical examples that are worth mentioning are clofazimine and derived structures that were clinically tested as antituberculosis agents,^8–11^ Safranin-O with remarkable photodynamic antimicrobial activity^12–14^ and Neutral Red, which gave the name to the well-known Neutral Red uptake assay, providing a quantitative estimation of cell viability/cytotoxicity (Fig. 1).^15,16^ Neutral Red has also been studied as a theranostic agent *in vitro*, however, *in vivo* testing is being limited by its absorption at high energy (*λ*_abs_ = 530 nm), which do not match the biological transparency window.^17,18^

For bio-imaging and photodynamic therapy purposes, the increased availability of laser sources and of diagnostic tools pushes the research towards the development of far-red and near-infrared (NIR) dyes, offering superior efficiency in terms of imaging resolution and safer incident light, respectively.^19,20^ Up to now, only few works report efficient strategies to redshift the optical properties of amino-phenazinium chromophores towards the NIR (Fig. 1). For instance, the extension and enrichment of the π-system of Induline 6B *via* the introduction of phenyl rings at the nitrogen atoms was a successful approach and resulted in a red located absorption maximum (*λ*_abs_ = 605 nm).^2^ Notably, the rigidification of the chromophore core, a strategy extensively applied for the design of NIR dyes^21–25^ was also tested in case of the phenazine derivatives. In a study described by Gloster and coworkers the incorporation of a peripheral tetrahydro-pyrazino moiety to the phenazine core led to a redshift of the absorption to a *λ*_max_ value of 607 nm *vs.* 564 nm for the non-rigidified congener. The induced restricted nitrogen atoms rotation eliminates the possibility of a twisted intramolecular charge transfer complex, improving also both the fluorescence and singlet oxygen quantum yields.^26^ In 2015, the attachment of strong guanidino donors, known for their modulation of the HOMO–LUMO gap, has been reported by the group of Himmel.^7^ Replacing classical amino functionalities by the guanidino groups provided a phenazine with strong orange-red emission (*Φ* = 0.39 at *λ*_em_ = 568 nm) and Stokes shift of up to 8750 cm^−1^ in H_2_O.

More recently, we reported the remarkable theranostic potential of triamino-phenazinium TAP (Fig. 1), which is synthesized in few steps and that features strong red fluorescence, two-photon absorption in the NIR and singlet oxygen generation capabilities, along with a selectivity for mitochondria staining.^27^ These unique assets prompted us to explore the possibility to tune the optical properties of TAP*via* straightforward modifications. We consequently report in this study the synthesis and photophysical characterization of a series of amino-phenazinium dyes, which show attractive optical features towards potent biological applications. The introduction of electron-withdrawing groups (EWG) on the available primary amine position has an impact on the structural and electrochemical properties of the dyes, but also resulted in a noticeable redshift of the absorption and emission bands towards the NIR domain, reaching wavelengths of interest corresponding to the biological transparency windows.^28^

## Results and discussion

### Synthesis

**Scheme 1:**
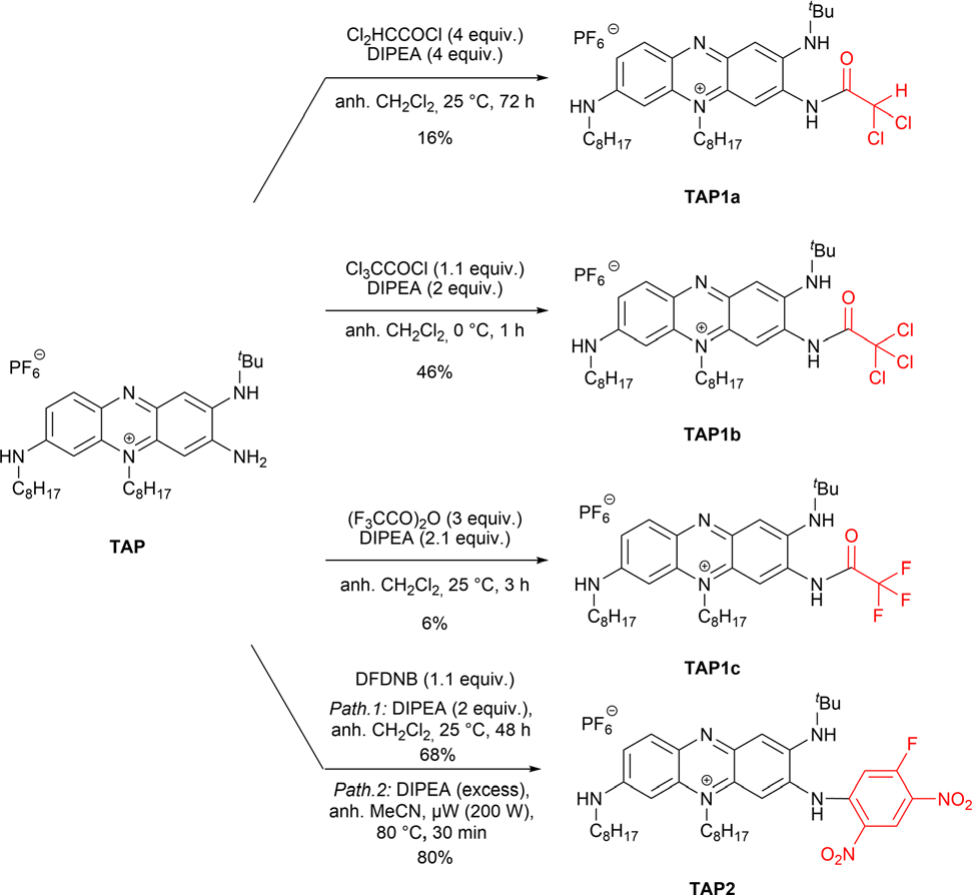
Functionalization of TAP leading to TAP1a–c and TAP2.

This work was initiated by considering the acylation of the primary amine function of TAP. Despite the limited nucleophilicity of the targeted amino group, which has a pronounced sp^2^ character due to its involvement in the positive charge delocalization,^27^ the acylation was envisaged using highly reactive and commercially available acyl chlorides or anhydrides (Scheme 1). Moreover, to overcome the acylation of the two secondary amines, high excess of the acylating reagents and elevated temperatures were avoided at first. The acylation of TAP using dichloroacetyl chloride was achieved at room temperature, affording TAP1a as a blue dye. The yield was slightly enhanced from 9% to 16% when an excess of the reagent was used along with a prolonged reaction time, from 16 to 72 hours. To achieve the acylation with the more reactive trichloroacetyl chloride, the reaction mixture was kept at 0 °C, affording the derivative TAP1b in 46% yield in only 1 hour. Unfortunately, the reaction yield could not be increased neither using a reagent (trichloroacetyl chloride) excess nor with longer reaction times. It is worth to note that the fluorinated phenazinium TAP1c was particularly challenging to isolate and required careful handling to avoid a possible hydrolysis of amide function. Then, the reactivity of TAP regarding nucleophilic aromatic substitution was investigated using the commercial building block 1,5-difluoro-2,4-dinitrobenzene (DFDNB). Due to the presence of two nitro moieties in *para* position, the C–F bond in DFDNB is strongly polarized, turning it into an electrophilic reagent of choice. The resulting blue-colored dye TAP2, introducing a fluoro-dinitrophenyl moiety, was isolated in 68% yield after purification by column chromatography. This later yield could be significantly improved to 80% with shorter reaction time by using microwave irradiation.

**Scheme 2:**
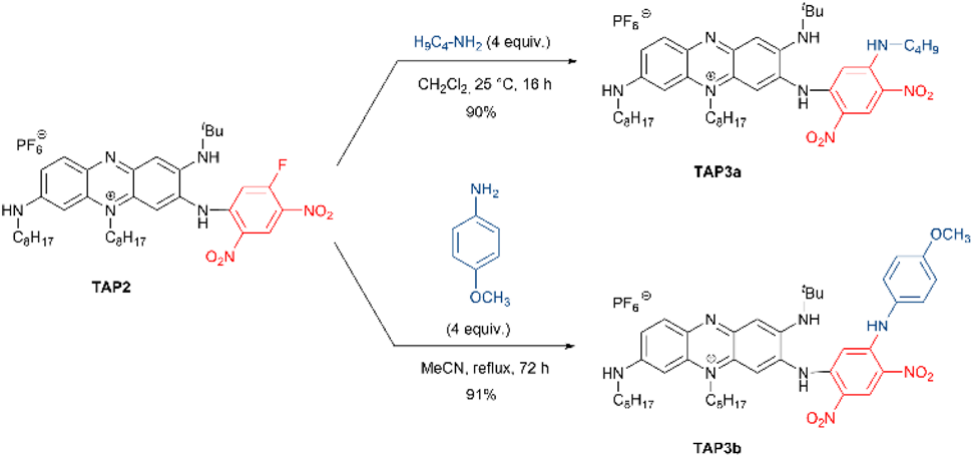
Aromatic nucleophilic substitution performed on TAP2 for the synthesis of dyes TAP3a,b.

Interestingly, the remaining electrophilic carbon on the highly electron-deficient fluoro-dinitrophenyl unit turns TAP2 into a viable platform in a strategy meant to use TAP2 for bioconjugation or attachment of other nucleophilic moieties for biological recognition. To confirm this hypothesis, we attempted to react TAP2 with commercial *n*-butylamine and *p*-anisidine, yielding the desired products TAP3a and TAP3b as purple solids with excellent yields (Scheme 2). A similar reaction performed with *m*,*m*′-CF_3_-aniline led to the recovery of the starting material, highlighting that such substitution was not possible with poorly nucleophilic amines.

### Structural analysis

**Fig. 2:**
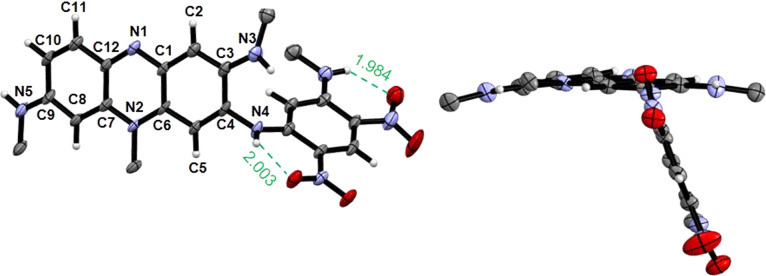
ORTEP views of TAP3a (right: side view, left: top view). Alkyl chains and counterion PF_6_^−^ omitted for clarity. Ellipsoids drawn at 50% probability. Selected bond lengths (Å): N(1)–C(1) 1.365(7), N(1)–C(12) 1.322(8), N(2)–C(6) 1.379(8), N(2)–C(7) 1.367(7), N(3)–C(3) 1.368(8), N(4)–C(4) 1.417(7), N(5)–C(9) 1.334(8), C(1)–C(2) 1.412(9), C(1)–C(6) 1.420(8), C(2)–C(3) 1.374(8), C(3)–C(4) 1.444(9), C(4)–C(5) 1.372(9), C(5)–C(6) 1.404(7), C(7)–C(8) 1.405(9), C(7)–C(12) 1.450(8), C(8)–C(9) 1.392(8), C(9)–C(10) 1.432(9), C(10)–C(11) 1.351(9), C(11)–C(12) 1.416(8).

All functionalized phenaziniums were characterized through NMR and HRMS measurements, and their cationic nature was confirmed by the presence of the hexafluorophosphate counterion (see NMR spectra in Fig. S10–S29, ESI†). Additionally, single crystals of TAP3a, suitable for XRD analysis, were obtained by slow diffusion of pentane in a dichloromethane (DCM) solution of the compound. The X-ray structure depicted in Fig. 2 fully established the tricyclic and planar phenazinium core with a maximum deviation from planarity of 0.100 Å (for the N(2) atom). In contrast with the parent dye TAP where the positive charge is stabilized by delocalization involving the lone pair of N(4) (delocalization on the N(4)–C(4)–C(5)–C(6)–N(2) segment), the N(4) atom in TAP3a is strongly conjugated with the nitro function (sp^2^ character). As a result, the positive charge is now stabilized by intramolecular delocalization involving N(5) over the N(2)–C(7)–C(8)–C(9)–N(5) unit. Moreover, we observe that the amino-dinitrophenyl substituent adopts a twisted position, at an angle of 72° in respect to the phenazinium plane, probably due to a simultaneous effect of steric hindrance and H-bond interactions.

### Electrochemical properties

**Fig. 3:**
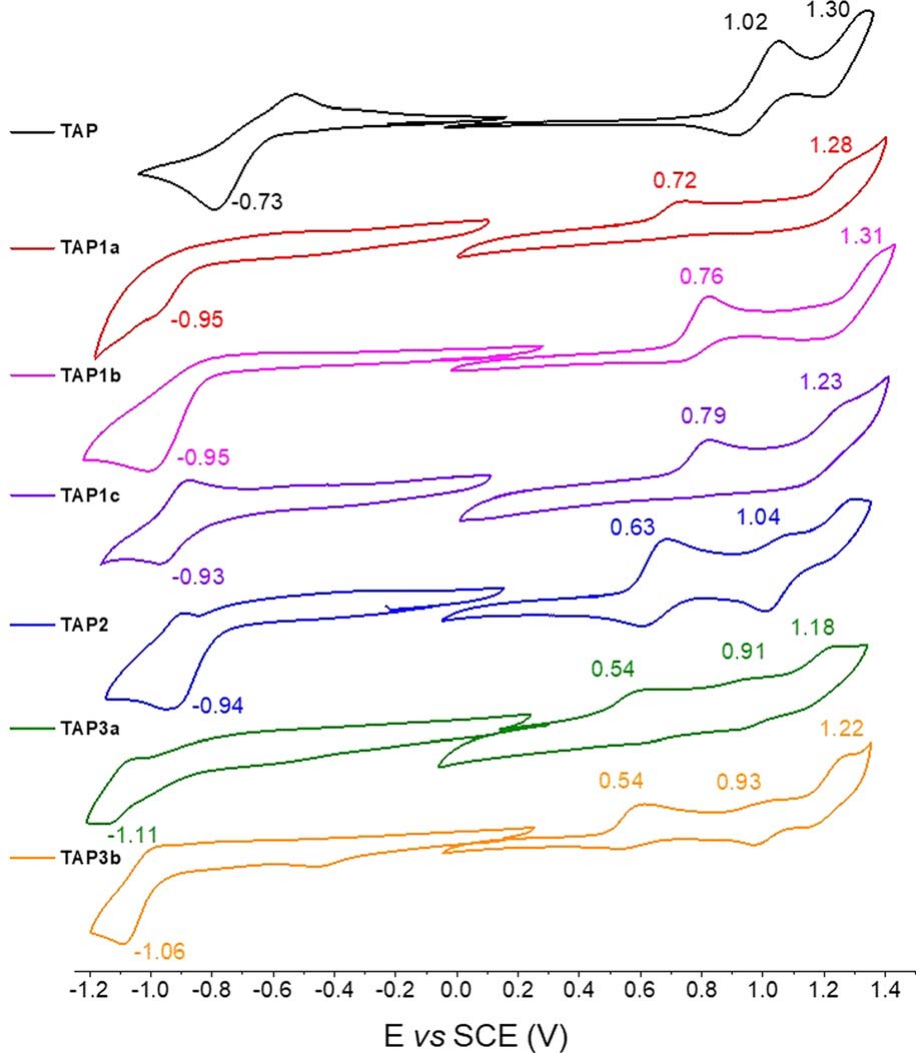
Cyclic voltammograms of TAP1–3 recorded in DCM (10^−3^ M) in the presence of tetra-*n*-butylammonium hexafluorophosphate as supporting electrolyte (10^−1^ M), with a scan rate of 100 mV s^−1^.

The parent phenazinium TAP as well as the modified dyes TAP1–3 were investigated by cyclic voltammetry, to gain insights into the substituent effect on the redox potential values (Fig. 3). Surprisingly, while the unsubstituted TAP presents a reduction process at −0.73 V/SCE, the introduction of electron-withdrawing moieties shifts the reduction potential of the substituted compounds to more negative values *ca.* −0.94 V/SCE, with no significant difference between the four dyes. This observation suggests that the reduction process does not occur at the same site upon introduction of the EWG. For the TAP3 series, the reduction gets even more cathodically shifted to −1.11 and −1.06 V/SCE respectively, which is explained by the introduction of an additional amine. On the same trend, the first oxidation event becomes easier, shifting from 1.02 V/SCE for the unsubstituted TAP to 0.72–0.79 V/SCE for the acylated compounds and 0.63 V/SCE for fluoro-dinitrophenyl bearing TAP2. The TAP3 dyes are prone to oxidation at even lower potentials of 0.54 V/SCE. Overall, the electrochemical gap is being reduced for all the functionalized species TAP1–3 compared to the unsubstituted congener TAP, which is consistent with the recorded optical trend (*vide infra*).

### Photophysical properties

**Fig. 4:**
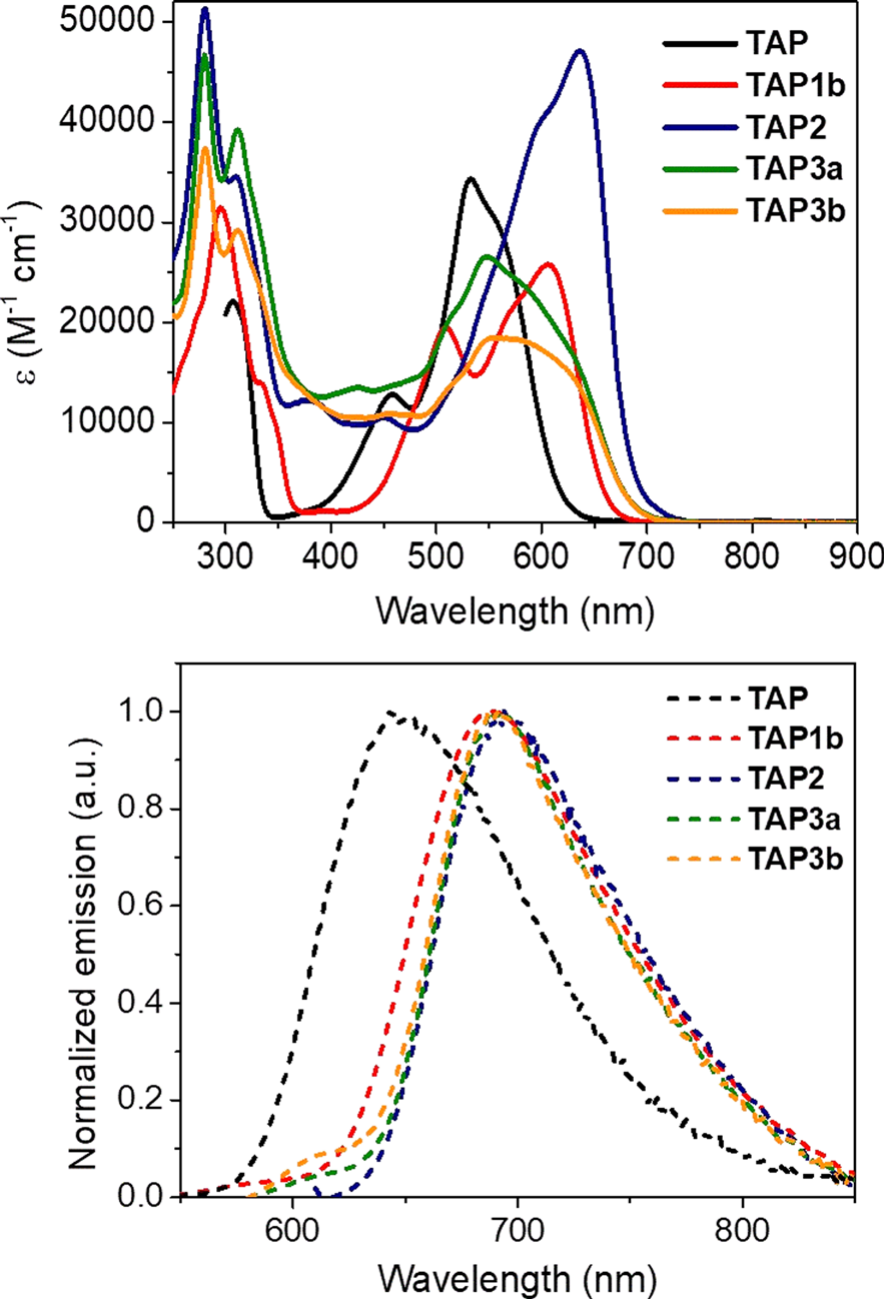
Electronic absorption and normalized emission spectra of the parent TAP and isolated TAP1b, TAP2 and TAP3a,b dyes in DCM.

Afterwards, we investigated the absorption and emission properties, notably aiming to explore the potential modulation of the optical characteristics of the post-functionalized derivatives, and their consistence with the reduction of the electrochemical gap. Compared to the parent derivative TAP, for which the absorption is centered in the green region (*λ*_max_ = 533 nm, Fig. 4 and Table 1), the acylated dyes TAP1a–c all display redshifted absorptions peaking at 606 nm, with a shoulder at 508 nm, and molar extinction coefficients (*ε*) in the order of 20 000–25 000 M^−1^ cm^−1^ (Table 1, Fig. 4, and S1–S3, ESI†). The trifling differences in absorption maxima for the TAP1 series points out a negligible effect of the electron-withdrawing amide nature. The EWG effect is more pronounced for the fluoro-dinitrophenyl substituted TAP2, which lowest energy absorption band is peaking at 635 nm with *ε* = 45 000 M^−1^ cm^−1^ (Table 1, Fig. 4 and S4 in the ESI†). The replacement of the electron-withdrawing fluorine atom with alkyl- or arylamine donors in TAP3a,b is accompanied in both cases by a blueshift towards the green region, with maxima found at 548 nm and 552 nm, respectively (, Table 1, Fig. 4, S6 and S7 in the ESI†).

**Table 1:** Summary of the optical properties for the isolated TAP1–3 and parent dye TAP

Dye	Solvent	*λ*_max_ (nm), (*ε* (M^−1^ cm^−1^))	*λ*_em_ (nm)	ΔSS^a^ (cm^−1^)	*Φ* b	*τ* (ns)	*k*_R_ c (10^6^ s^−1^)	*k*_NR_ c (10^6^ s^−1^)
TAP	MeCN^d^	553 (43 140), 465 (16 340)	637	2380	63%	6.6	95	56
	DCM	533 (34 340)	645	3260	69%	6.1	113	50
TAP1a	DCM	606 (19 000), 560 (16 880), 508 (15 200)	703	2280	2.7%	4.2	6.4	231
TAP1b	DCM	606 (25 750), 508 (19 500)	685	1900	3.9%	4.7	8.3	204
TAP1c	DCM	606 (20 260), 569 (18 190), 507 (17 000)	687	1950	5.2%	5.6	9.3	169
TAP2	DCM	635 (45 000), 596 (39 560), 450 (10 500)	694	1340	<1%	1.0	10	990
TAP3a	DCM	548 (26 570)	694	3840	6.3%	3.6	18	260
TAP3b	DCM	552 (18 500)	694	3710	1%	2.6	4	380

a ΔSS: Stokes shift, calculated as a difference between the absorption and emission maxima.

b Relative fluorescence quantum yields measured with oxazine 725 perchlorate as reference (*ϕ* = 11% in EtOH, *λ*_ex_ = 620 nm) for TAP1–3, or rhodamine B as reference (*ϕ* = 70% in MeOH, *λ*_ex_ = 530 nm) for TAP.

c Radiative and non-radiative decay constants calculated using the following equations: *k*_R_ = *ϕ*/*τ* and *k*_NR_ = (1 − *ϕ*)/*τ*.

d From ref. 27.

The unsubstituted TAP displays a bright emission with a narrow red-located (*λ*_em_ = 645 nm) fluorescence band (Fig. 4, Table 1). The introduction of electron-withdrawing substituents considerably shifts the emission maxima towards the far-red and even NIR regions. For the acylated TAP1a, TAP1b and TAP1c the band is peaking at 703, 685 and 687 nm (Table 1, Fig. 4, and S1–S3 in the ESI†) with quantum yields of 2.7%, 3.9% and 5.2%, respectively (Table 1), while for TAP2 we witness a fluorescence band centered at 694 nm and tailing in the NIR region. Upon the modification of the primary amine unit, there is a remarkable increase in the nonradiative deexcitation of all the dyes, from four-fold to twenty-fold, compared to the parent TAP (see Table 1), and hence a marked decrease of quantum yield. The different alkyl- or arylamine substituent in TAP3a,b has no effect on the emission band (Fig. 4 and Table 1) but an unexpected drop in fluorescence efficiency is noticed for the arylamine functionalized derivative.

**Fig. 5:**
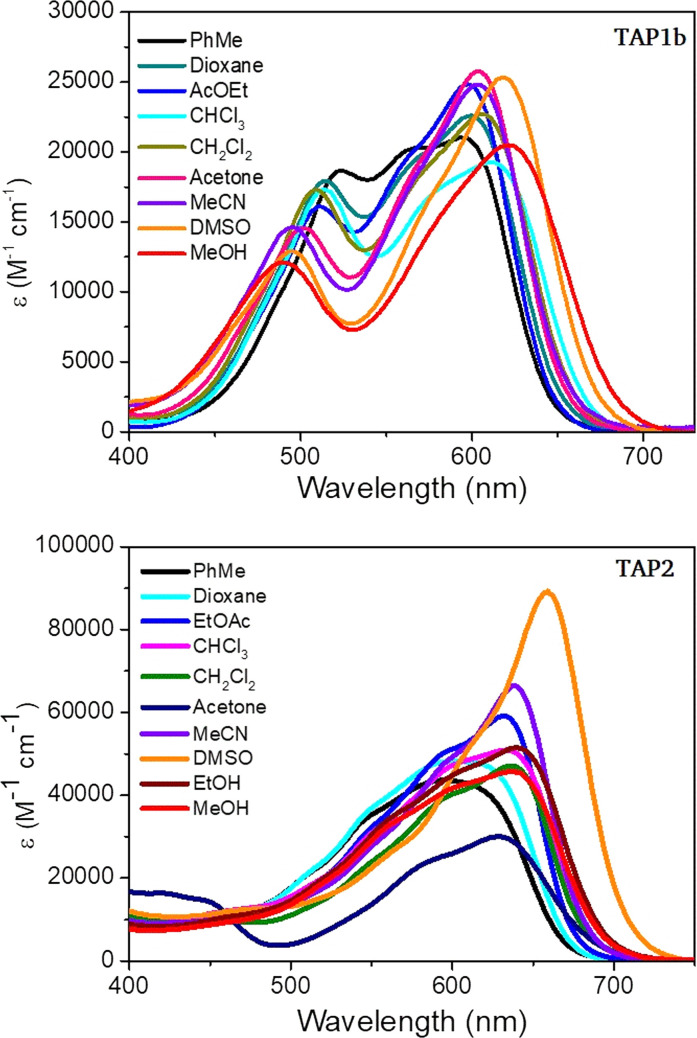
Electronic absorption solvatochromism of TAP1b and TAP2 (*c* = 1.15 × 10^−5^ M and 1.3 × 10^−5^ M respectively).

The excellent solubility of the isolated phenaziniums allowed to record a solvatochromism study, for which purpose we selected TAP1b and TAP2. The results highlight a trend of the absorption band to become narrower and accompanied by a moderate bathochromic shift with the increased polarity of the medium, ranging from 594 nm in toluene to 621 nm in MeOH for TAP1b and from 592 nm in toluene to 659 nm in DMSO for TAP2 (Fig. 5). The weak positive solvatochromism is also a feature of the parent compound TAP. When the fluorescence of TAP3a,b was measured in MeOH and dioxane, a strong redshift was observed in the former solvent (Fig. S8 in the ESI†). This tendency suggests that the solvent-mediated stabilization of the excited-state is higher in protic solvents, leading to redshifted emissions with higher Stokes shifts in methanol of approximately 4400 cm^−1^*vs.* 2150 cm^−1^ recorded for the reference compound TAP (see Table 1 for DCM). The influence of the protic nature of MeOH could not be verified as the compounds TAP2 and TAP3a,b displayed quenched emission in MeCN and DMSO. Compared to the parent TAP2 derivative, the TAP3a,b dyes showed increased values of emission efficiency (Table 1).

**Fig. 6:**
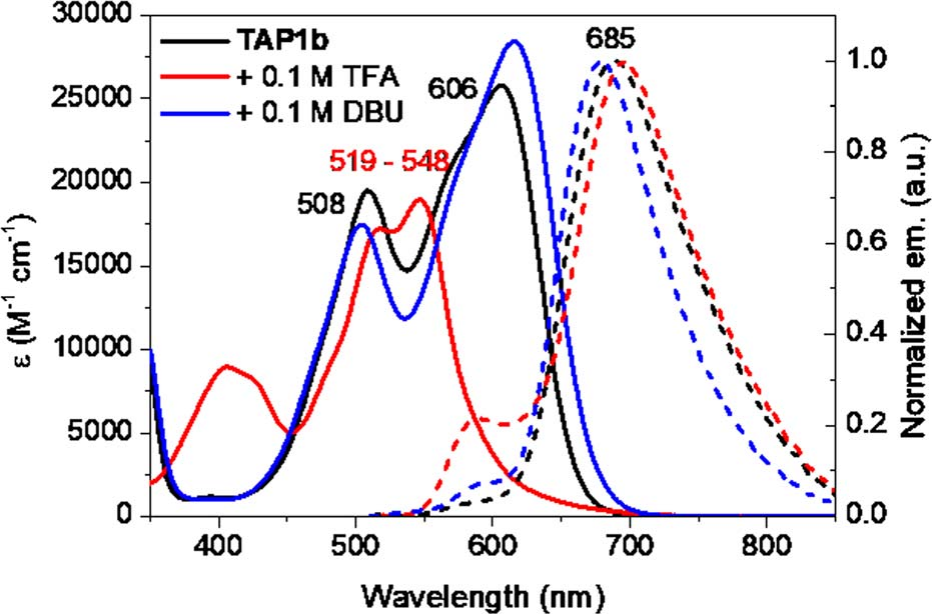
Electronic absorption (solid lines, *c* = 1.15 × 10^−5^ M) and normalized emission (dashed lines) spectra of TAP1b in DCM (*λ*_ex_ = 510 nm), DCM + TFA (*λ*_ex_ = 510 nm) and DCM + DBU (*λ*_ex_ = 565 nm).

The acidochromic properties of the new dyes were shortly investigated as well, knowing the several protonation states reported for TAP.^27^ Upon addition of trifluoroacetic acid (TFA), the absorption spectra of the TAP1a–c series is blueshifted towards 519–548 nm (Fig. 6 and S2–S3 in the ESI†). This trend suggests the protonation of the amino-phenazinium, in a similar manner to the parent phenazinium TAP and is explained by a rather localized electronic structure. On the emission spectra, this translates into the appearance of a shoulder along with the maintaining of the main emission band, contrary to parent derivative TAP, whose protonation induces the loss of emission properties. The addition of 1,8-diazabicyclo[5.4.0]undec-7-ene (DBU) leads to almost negligible hyperchromic and bathochromic absorption shifts, with a trifling hypsochromic effect on the fluorescence spectra. Considering the acidity of the NH proton linked to the acyl group, this outcome might be explained by the possibility that compounds TAP1a–c are partially deprotonated in the diluted solutions, DBU addition only increasing the process.

As expected, upon introduction of a strong EWG, a high excess of acid is needed to partially protonate TAP2, and this event is accompanied by a less intense and blueshifted absorption band as well as a notably blueshifted emission (see Fig. S4†). Addition of DBU induces the appearance of an additional shoulder, while maintaining the main absorption band. The already weak emission of TAP2 is even more quenched in basic medium. To eliminate the possibility that the compound is already deprotonated in solution (due to water traces), the measurements were repeated in anhydrous DCM, additionally dried over molecular sieves (Fig. S5 in the ESI†). We could witness the same behavior upon the addition of TFA. Basified solution, on the contrary, showed an evolution within 1 hour timeframe, the deprotonated species exhibiting a blueshifted absorption (*λ*_max_ = 543 nm). The transition turned to be reversible upon TFA addition, pointing towards a slow deprotonation process, and therefore ruling out an eventual nucleophilic reaction with DBU.

Finally, the fluorescence lifetime measurements performed on the starting derivative TAP and the functionalized series TAP1–3 return rather long values for NIR emitters, ranging from 1 to 6.6 ns. These results can be easily correlated with the efficiency of the emission process and are explained by the high rigidity of the tricyclic phenazinium core (Table 1 and Fig. S9 in the ESI†).

### Theoretical calculations

**Fig. 7:**
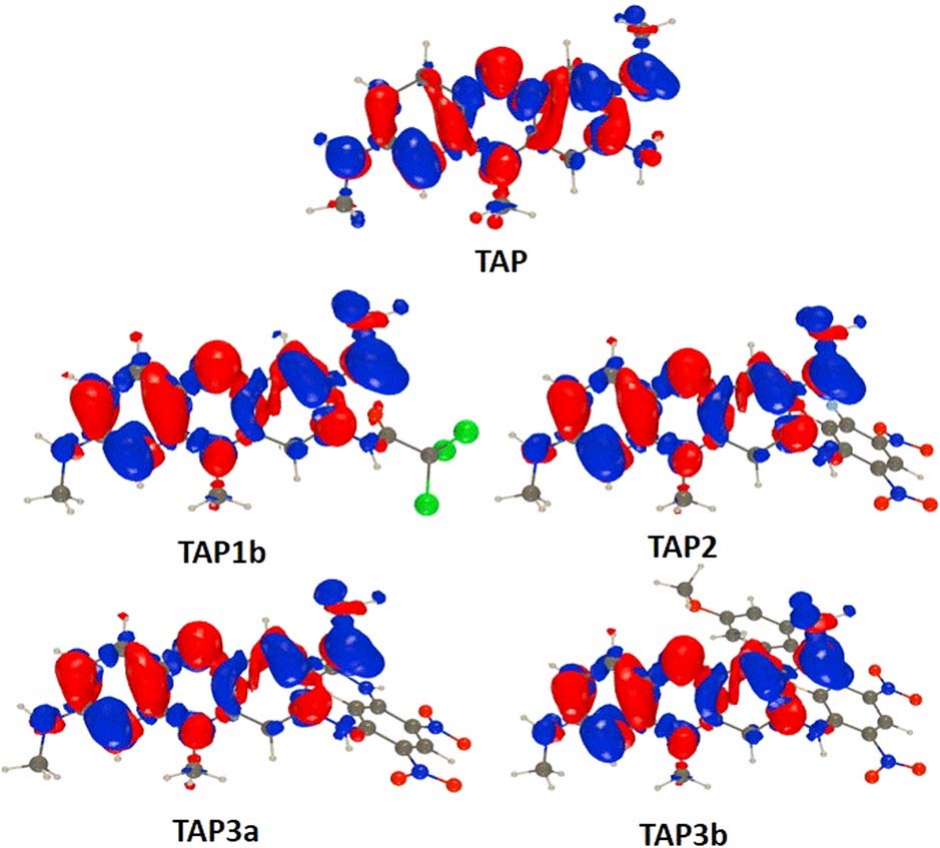
Electron density difference plots for the lowest excited-state (S_0_–S_1_) of the investigated compounds. The red and blue lobes correspond to regions of increase and decrease of density upon absorption. Contour threshold: 0.001 au.

To obtain additional insights we performed theoretical calculations using TD-DFT and CC2, as detailed in the ESI.†Fig. 7 shows electron density difference (EDD) plots corresponding to the absorption. For TAP, one can see alternance of blue (loss of density) and red (gain of density) regions, the two intracyclic nitrogen atoms gaining density whereas the side amino groups lose some density. This alternance is quite typical of a cyanine-like character. This is confirmed by the very small charge-transfer character determined for TAP (see Table S1 in the ESI†). Theory foresees a 0–0 energy of 1.97 eV (630 nm, see Table S2 in the ESI†) for TAP, which fits quite well the experimental absorption–emission crossing point (2.05 eV, see Fig. S1†). All substituted systems that were modelled, namely TAP1b, TAP2, TAP3a and TAP3b, display similar EDD plots, that differ from the one of TAP by the significant asymmetry between the contributions of the side amino groups (Fig. 7). In other words, the transition gains a mild yet non-trifling CT character.

This is confirmed by the data of Table S1:† the absorption of TAP1–3 induces the transfer of *ca*. 0.5 electron over 1.2–1.6 Å. This variation of excited-state nature is accompanied by significant redshifts of the absorption and emissions (Table S2†), which fits the measurements. The two effects (CT character and redshift), naturally explain the drop in fluorescence quantum yield from TAP to TAP1–3. One aspect that remains intriguing however is the strong difference of quantum yield between TAP3a and TAP3b, despite very similar spectral properties. In TPA3a, the computed TD-DFT S_0_–S_1_ absorption is bright and mainly corresponds to a HOMO–LUMO excitation, both orbitals being mainly centered on the phenazium core, *i.e.*, the expected trend is obtained. In contrast, in TAP3b, the computed TD-DFT S_0_–S_1_ excitation can be mainly ascribed to a HOMO-1 to LUMO transition, the HOMO being localized on the side group (see the ESI† for details). On can therefore suspect the presence of photoinduced electron transfer in TAP3b.

## Conclusions

In conclusion, we have investigated the straightforward post-functionalization of a triamino-phenazinium dye TAP and showed that the introduction of electron-withdrawing groups on the reactive primary amine function has a dramatic impact on the electron density of the phenazinium core (TAP1–3). The synthetic approach, employing a one-step acylation or nucleophilic aromatic substitution, induces a drastic colour change and enables the easy access to dyes with red to far-red absorption and NIR emission properties, making them attractive candidates for biological staining and therapeutic applications. Notably, the TAP2 derivative, bearing a reactive fluorine moiety holds promise as a versatile platform for substitution reactions with nucleophiles and easy bioconjugations with drugs, sugars or proteins. Alternatively, the incorporation of fluorine moieties can enhance the pharmacokinetic profile and staining capacities of fluorophores. Moreover, an advantageous combination of complementary fluorescence imaging and PET (positron emission tomography) scan technique, for example, open the possibility for a superior resolution imaging probe, especially for the oncology field.

## Author contributions

S. P. and O. S. conceived, designed and supervised the project. T. M., J.-F. L. and G. C. designed and conducted the experiments and collected and analysed the results. D. J. performed and analyzed the theoretical calculations. T. M. and S. P. drafted the manuscript. All authors read, reviewed, and approved the final draft.

## Conflicts of interest

There are no conflicts to declare.

## Supplementary Material

RA-014-D4RA03245D-s001

RA-014-D4RA03245D-s002
