# Supplementary material for: Post-functionalization of triamino-phenazinium dyes to reach near-infrared emission
Source: RSC Adv. 2024 Jun 17;14(27):19257–63. doi: 10.1039/d4ra03245d (PMC11180995; doi:10.1039/d4ra03245d)
Supplement: RA-014-D4RA03245D-s001 [file RA-014-D4RA03245D-s001.pdf]

Electronic Supplementary Material (ESI) for RSC Advances.
This journal is © The Royal Society of Chemistry 2024

Supporting information

Post-Functionalization of Triamino-Phenaziniums Dyes to Reach Near-Infrared Emission

Tatiana Munteanu,[a] Jean-François Longevial,[a,b] Gabriel Canard,[a] Denis Jacquemin,[c,d] Simon Pascal,*[a,c] Olivier Siri*[a]

[a] Aix Marseille Univ., CNRS UMR 7325 Centre Interdisciplinaire de Nanoscience de Marseille (CINaM), Campus de Luminy, 13288 Marseille cedex 09, France.

[b] Université de Lorraine, LCP-A2MC, F-57000 Metz, France.

[c] Nantes Université, CEISAM UMR 6230, CNRS, Nantes F-44000, France.

[d] Institut Universitaire de France (IUF), Paris, France.

TABLE OF CONTENT

I. GENERAL REMARKS AND ANALYSIS CONDITIONS 2

II. ADDITIONAL OPTICAL SPECTRA 5

III. SYNTHETICS PROTOCOLS AND CHARACTERIZATIONS 10

IV. NMR SPECTRA 15

V. MASS SPECTROMETRY 25

VI. X-RAY DIFFRACTION 30

VII. THEORETICAL CALCULATIONS 31

VIII. REFERENCES 42

I. GENERAL REMARKS AND ANALYSIS CONDITIONS

Reagents. All reagents were purchased from Alfa Aesar or Sigma Aldrich and were used without further purification. When heating was required, oil bathes were used. Column chromatography was performed using silica gel (60-120 mesh) and alumina neutral (63-200 µm, Beckmann grade **I**). Analytical thin layer chromatography (TLC) was performed on precoated silica gel-60 F254 (0.5 mm) aluminium plate or precoated Al_2O_3 gel-60 neutral (0.2mm) aluminium plate. Visualization of the spots on TLC plates was achieved by exposure to UV light. Filter aid was performed using Celite AW standard Supercel® or Celite® type 545. Unless otherwise specified, the desired product was dried under vacuum (< 10 mbar) over 5 hours at room temperature.

Analytical methods and apparatus. **^{1}H NMR** spectra were recorded on a JEOL ECS400 spectrometer operating at 400 MHz or with a Bruker Avance NEO 600 MHz Spectrometer. **^{13}C{^{1}H} NMR** spectra were recorded on a JEOL ECS400 spectrometer operating at 100 MHz, respectively or on a Bruker Avance DRX 500 NMR spectrometer equipped with a double resonance broadband fluorine observe (BBFO) 5 mm probe head. Chemical shifts are reported in delta (δ) units, expressed in parts per million using the residual protonated solvent as an internal standard (For proton: $CDCl_3$, 7.26 ppm; CD_2Cl_2, 5.32 ppm; For ^{13}C**{^{1}H}**: $CDCl_3$, 77.0 ppm; $C_4D_2Cl_4$, 73.7 ppm). The multiplicity of signals is designated by the following abbreviations: s, singlet; br s, broad singlet; d, doublet; br d, broad doublet; t, triplet; m, multiplet. Coupling constants, *J*, are reported in Hertz (Hz). All the NMR spectra, except the ^{13}C**{^{1}H}** for **TAP3a** were run at 300 K. ^{13}C**{^{1}H}** NMR of **TAP3a** was performed at 343 K. **High resolution mass spectrometry** (HRMS-ESI) analyses were performed on a QStar Elite (Applied Biosystems SCIEX) spectrometer or on a SYNAPT G2 HDMS (Waters) spectrometer by the *Spectropole* of Aix-Marseille University. These two instruments are equipped with an electrospray ionization (ESI) or a MALDI source and a TOF analyzer. **Electronic absorption** spectra were measured on a Varian Cary 50 UV-vis or on an Agilent Cary 5000 UV-vis-NIR or on a Perkin-Elmer Lambda EZ 210 UV-vis spectrophotometer.

Crystallography. Crystals were mounted on a Rigaku Oxford Diffraction SuperNova diffractometer and measured at 293 K at the Cu radiation (λ= 1.54184 Å). Data collection, reduction and multiscan ABSPACK correction were performed with CrysAlisPro (Rigaku Oxford Diffraction). Using Olex2[1] the structures were solved with the ShelXT[2] structure solution program using Intrinsic Phasing and refined with ShelXL[2] using least-square minimization.

Electrochemistry. Cyclic voltammetry (CV) data were recorded using a BAS 100 (Bioanalytical Systems) potentiostat and the BAS100W software (v2.3). All the experiments were conducted in a standard one-compartment using a three electrodes setup: a Pt working electrode (Ø = 1.6 mm), a Pt counter electrode and an Ag/AgCl reference electrode (filled with a 3 M NaCl solution). Tetra-*n*-butylammonium hexafluorophosphate ([TBA][PF_6]) was used as supporting electrolyte (10^{-1} M), with a concentration of the electro-active compound of *ca*. 10^{-3} M. The reference electrode was calibrated using ferrocene (E°(Fc/Fc^+) = 0.46V/SCE).[3] The scan rate was 100 mV/s. The solution was degassed

using argon before recording each reductive scan, and the working electrode (Pt) was polished before each scan recording.

Fluorescence. Emission spectra were measured using a Horiba-Jobin Yvon Fluorolog-3 spectrofluorometer equipped with a three-slit double-grating excitation and a spectrograph emission monochromator with dispersions of 2.1 nm mm^{-1} (1200 grooves per mm). A 450 W xenon continuous wave lamp provided excitation. The luminescence of diluted solutions was detected at right angle using 10 mm quartz cuvettes.

Fluorescence quantum yields Φ were measured in diluted solutions with an optical density lower than 0.1 using the following equation:

$$\frac{\Phi_x}{\Phi_r} = \left(\frac{A_r(\lambda)}{A_x(\lambda)}\right)\left(\frac{n_x^2}{n_r^2}\right)\left(\frac{D_x}{D_r}\right) \quad (eq.1)$$

where A is the absorbance at the excitation wavelength (λ), *n* the refractive index and *D* the integrated intensity. "*r*" and "*x*" stand for reference and sample. The fluorescence quantum yields were measured with oxazine 725 perchlorate as reference (ϕ = 11% in EtOH, λ_{ex} = 620 nm) for **TAP1–3**, or rhodamine B as reference (ϕ = 70% in MeOH, λ_{ex} = 530 nm) for **TAP**.[4]

Fluorescence lifetime. Short luminescence decay was monitored using a Horiba DeltaFlex modular fluorescence lifetime system equipped with a TC-SPC. Excitation was performed using a DeltaDiode (Model: DD-440L; peak wavelength: 438 nm) and Ludox in distilled water was used to determine the instrumental response function (IRF) used for deconvolution, which was performed using the EzTime software. All the measurements were fitted with single exponential decays.

Theoretical calculations. We have performed the DFT and TD-DFT calculations with Gaussian 16.[5,6] The long alkyl chains were replaced by Me groups for obvious computational reasons. Default Gaussian16 thresholds and algorithms were used but for an improved optimization threshold (10^{-5} au on average residual forces), a stricter self-consistent field convergence criterion (10^{-10} a.u.) and the systematic use of the *superfine* DFT integration grid, the denser grid available in Gaussian.

Firstly, the S_0 geometries have been optimized with DFT and the vibrational frequencies have been analytically determined, using the M06-2X *meta*-GGA hybrid exchange-correlation functional.[7] These calculations were performed with the 6-311G(d,p) atomic basis set in solution using the PCM model.[8] Dichloromethane was considered as solvent since this solvent is ideal for PCM applications (no H-bonds). Secondly, starting from the optimal ground-state geometries, we have used TD-DFT with the same functional and basis set to optimize the S_1 geometry and compute analytically the vibrational frequencies. Again, solvent effects were accounted with PCM for using the default Gaussian16 model for excited-state optimization. All optimized (both ground and excited states) structures correspond to true minima of the potential energy surface. Thirdly, the vertical transition energies were determined

with TD-DFT and the same functional, but a diffuse-containing basis set, namely 6-311+G(2d,p), in gas-phase as well as in solution using the cLR^2 variant of the PCM,[9] in its *non-equilibrium* limit.

As we are aware of the significant dependency of the TD-DFT results on the selected functional,[10] the obtained transition energies were also computed using CC2[11] with the Turbomole 7.3 code.[12] The CC2 energies were calculated in gas phase applying the resolution of identity scheme, and using the *aug*-cc-pVDZ atomic basis set. Combining the CC2 and TD-DFT data using a well-known protocol,[13] one can obtain accurate CC2-corrected estimates (our theoretical best estimates, TBE) of the absorption, emission and 0-0 energies that can be straightforwardly compared to experimental values.

II. ADDITIONAL OPTICAL SPECTRA

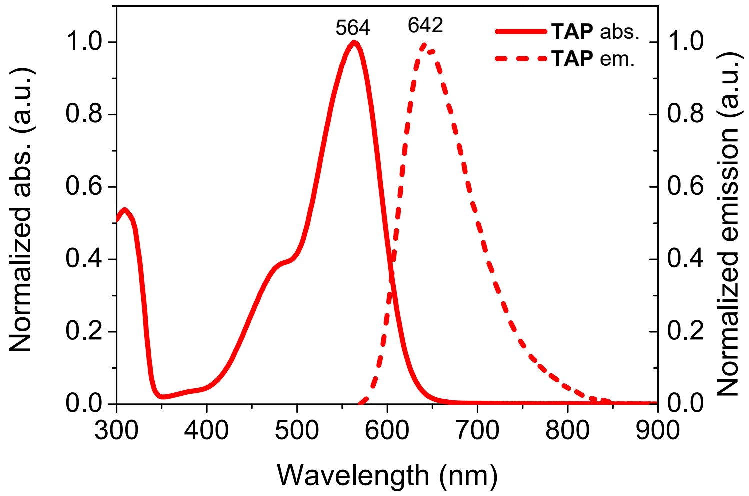

Figure S 1 Normalized electronic absorption et emission spectra of **TAP** in MeOH.

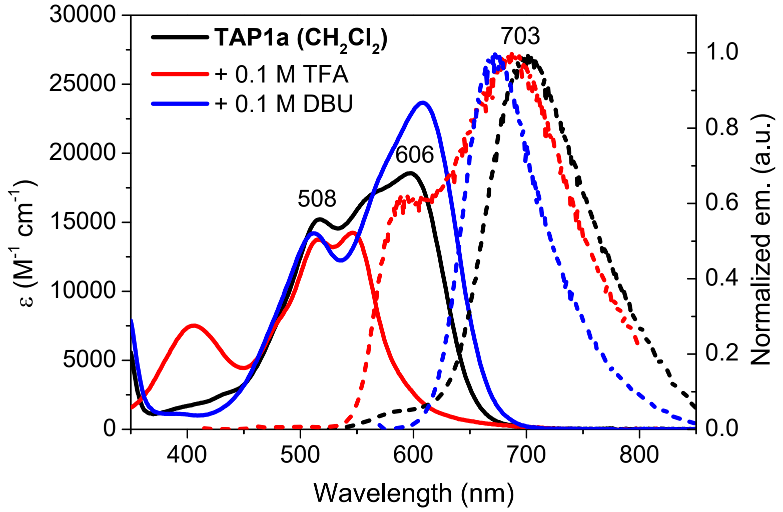

Figure S 2. Electronic absorption (solid lines, c = 1.7 × 10^{-5} M) and normalized emission (dashed lines) spectra of **TAP1a** in CH_2Cl_2 (λ_{ex} = 510 nm), CH_2Cl_2+TFA (λ_{ex} = 510 nm) and CH_2Cl_2+DBU (λ_{ex} = 565 nm).

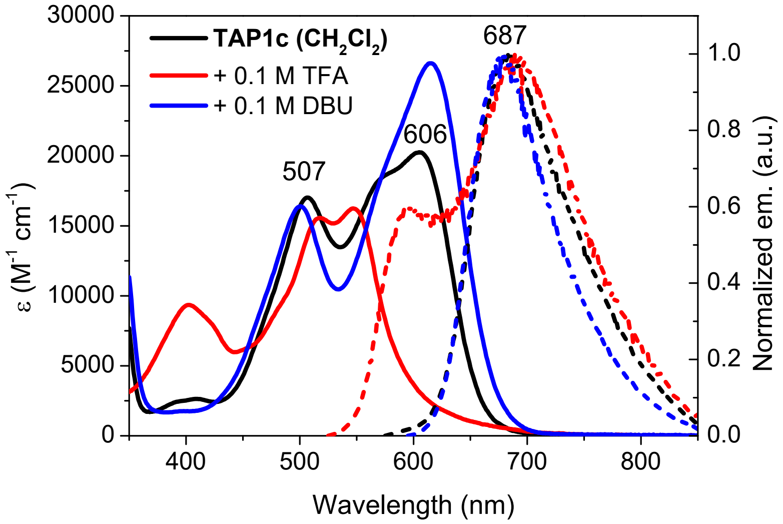

Figure S 3. Electronic absorption (solid lines, c = 1.92 × 10^{-5} M) and normalized emission (dashed lines) spectra of **TAP1c** in CH_2Cl_2 (λ_{ex} = 565 nm), CH_2Cl_2+TFA (λ_{ex} = 515 nm) and CH_2Cl_2+DBU (λ_{ex} = 585 nm).

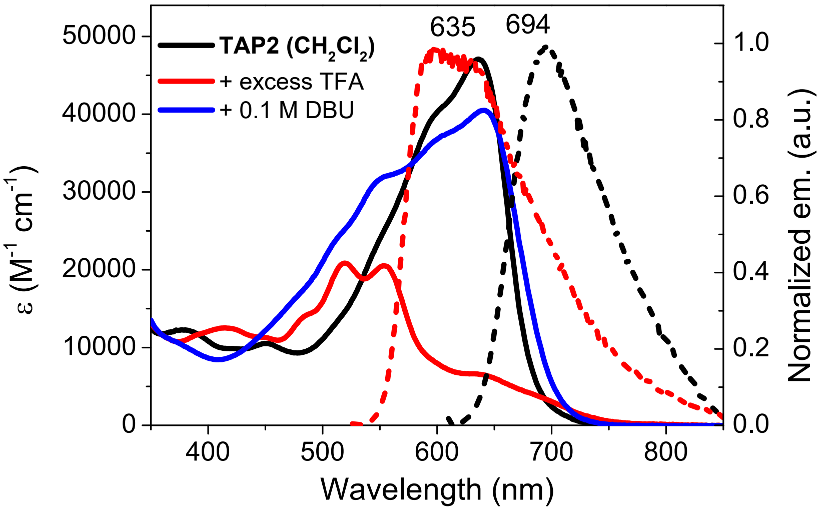

Figure S 4. Electronic absorption (solid lines, c = 1.3 × 10^{-5} M) and normalized emission (dashed lines) spectra of **TAP2** in CH_2Cl_2 (λ_{ex} = 600 nm), CH_2Cl_2+TFA (λ_{ex} = 500 nm).

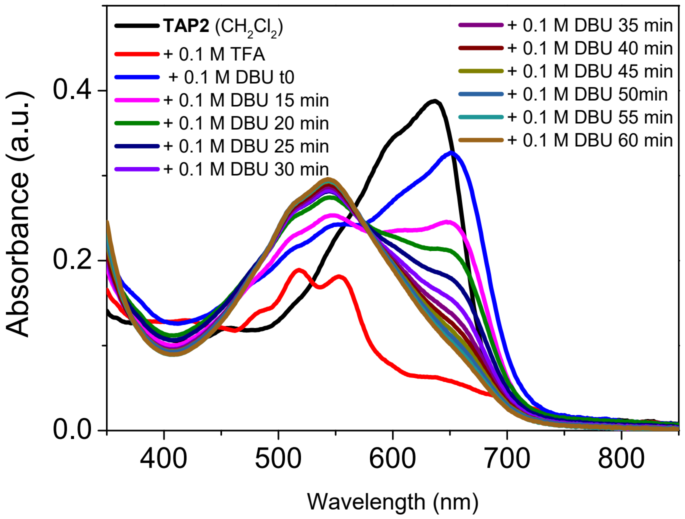

Figure S 5. Electronic absorption spectra of **TAP2** in CH_2Cl_2 (c = 1.2 × 10^{-5} M) and the evolution of **TAP2** in CH_2Cl_2 + 0.1 M DBU.

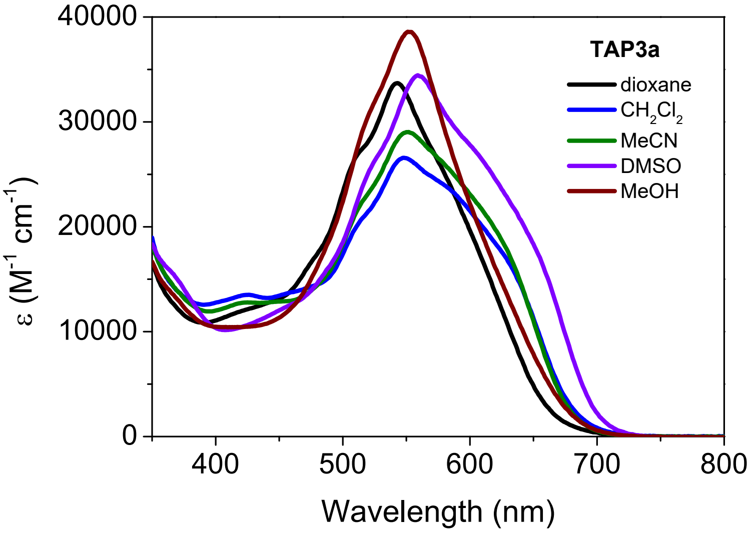

Figure S 6. Electronic absorption solvatochromism of **TAP3a** (c = 1.8 × 10^{-5} M).

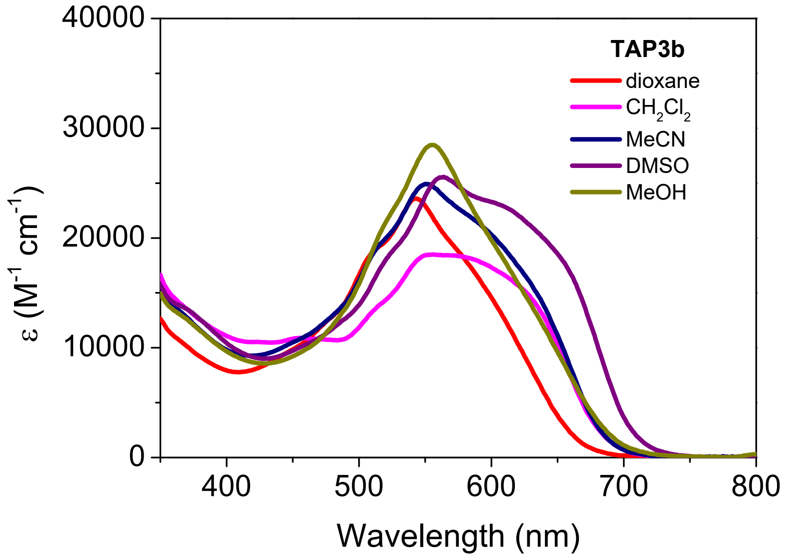

Figure S 7. Electronic absorption solvatochromism of **TAP3a** (c = 1.8 × 10^{-5} M).

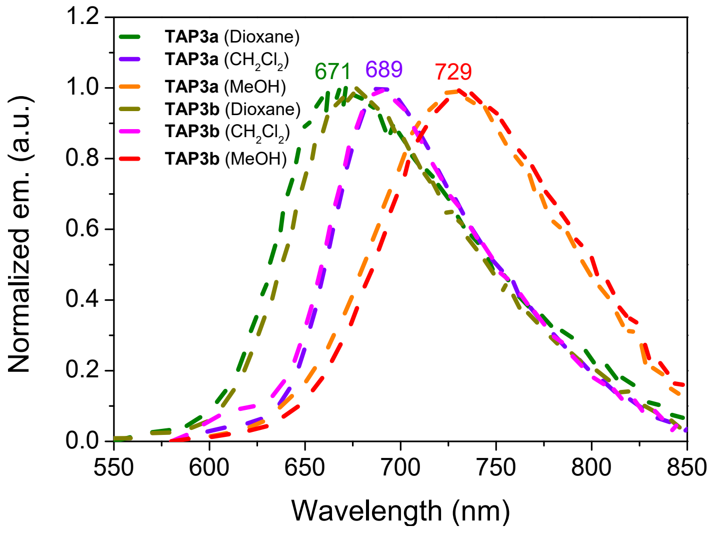

Figure S 8. Solvatofluorochromism of **TAP3**. Normalized emission spectrum in dioxane (λ_{ex} = 530 nm), CH_2Cl_2 (λ_{ex} = 540 nm) and MeOH (λ_{ex} = 540 nm).

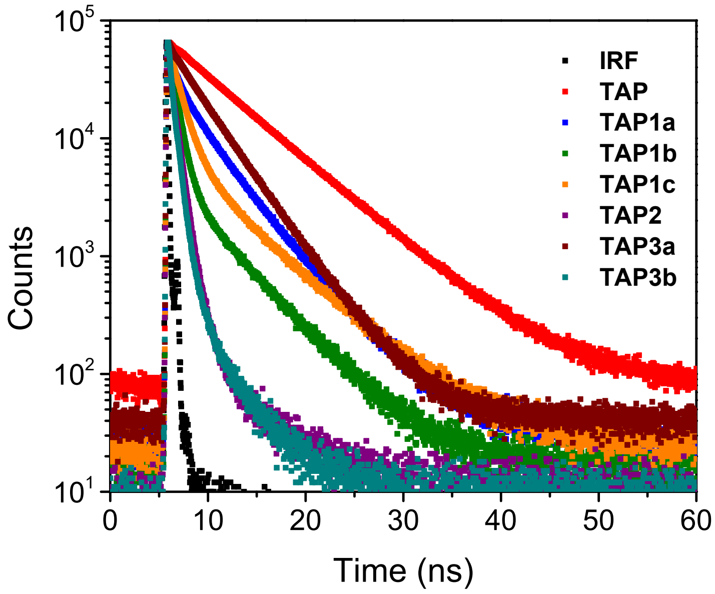

Figure S 9. Fluorescence decays measured by single photon counting of the emissive compounds in CH_2Cl_2 solutions. Excitation at 438 nm. IRF: instrument response function (Ludox).

III. SYNTHETICS PROTOCOLS AND CHARACTERIZATIONS

TAP

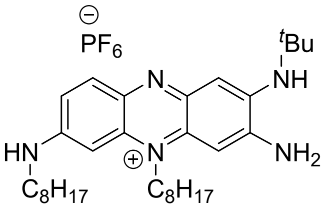

The phenazinium precursor **TAP** was synthesized according to the reported protocol.[14]

A solution of **TAP** precursor *N*[1]-(5-(*tert*-butylamino)-2,4-dinitrophenyl)-*N*[2], *N*[4]- dioctylbenzene-1,2,4-triamine (1.08 g, 1.84 mmol, 1 equiv.) in MeOH (70 mL) was hydrogenated (40 bars) in the presence of Pd/C (197 mg, 5 wt. %, 5% mol) and HCl (12M, 0.8 mL) for 6 hours. Then the mixture was stirred under air for 16 h. Pd/C was removed by filtration through a Celite® plug. After removal of the solvent under reduced pressure, the resulting solid was taken up with CH_2Cl_2 (100 mL), washed with 60 mL of an 0.1 M aqueous solution of KPF_6 then with 30 mL of water. The organic layers were collected, dried over anhydrous Na_2SO_4, filtered and the solvent was evaporated. The residue was subjected to silica gel chromatography using CH_2Cl_2/ MeOH (gradient from 98:2 to 95:5) as eluent to afford the product as a deep purple solid in 79% yield (952 mg, 1.46 mmol). NMR spectrum and electronic absorption of **TAP** was comparable to the previously reported ones.

R_f: 0.36 (SiO_2, CH_2Cl_2/ MeOH, 98:2). **^{1}H NMR ($CDCl_3$, 400 MHz):** δ = 7.70 (d, J^3 = 8.8 Hz, 1H, CH), 7.17 (s, 1H, CH), 7.11 (d, J^3 = 8.7 Hz, 1H, CH), 7.04 (s, 1H, CH), 6.39 (s, 1H, CH), 6.04 (br s, 2H, NH_2), 5.56 (br s, 1H, NH), 4.44 (t, J^3 = 7.8 Hz, 2H, CH_2), 4.09 (br s, 1H, NH), 3.31 (m, 2H, CH_2), 1.85 (m, 2H, CH_2), 1.76 (m, 2H, CH_2), 1.60 (m, 2H, CH_2), 1.53 (s, 9H, CH_3), 1.45 (m, 2H, CH_2), 1.25 (m, 16H, 8CH_2), 0.85 (m, 6H, 2CH_3).

TAP1a

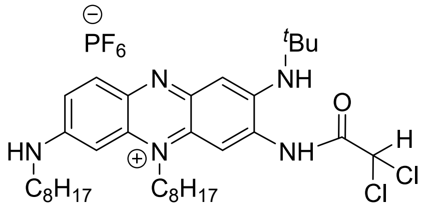

In a 10 mL two-neck flask, pre-dried under vacuum and purged with Argon, 50 mg (0.076 mmol, 1 equiv.) of **TAP** and 54 µL (0.304 mmol, 4 equiv.) of *N,N*-diisopropylethylamine were dissolved in 3 mL of anhydrous CH_2Cl_2. An ice bath was prepared to cool the mixture at 0 °C and 30 µL (0.304 mmol, 4 equiv.) of Cl_2HCOCl were added *via* a microsyringe. The reaction was stirred at 25 °C for 72 h, then it was taken in 50 mL of CH_2Cl_2, washed with 150 mL H_2O, and with 60 mL of an 0.1 M aqueous solution of KPF_6. The organic layers were collected, dried over anhydrous Na_2SO_4, filtered and the solvent was evaporated. The residue was subjected to column chromatography on aluminium oxide (neutral, Brockmann activity I) using CH_2Cl_2/AcOEt as eluent (gradient from 9:1 to 8:2). The blue fraction was collected and purified by silica gel chromatography, using CH_2Cl_2/MeOH (90:10) as eluent to afford the product as a blue solid in 16% yield (9 mg, 0. 011 mmol).

R_f: 0.56 (Al_2O_3, CH_2Cl_2/ AcOEt, 8:2). **R_f:** 0.35 (SiO_2, CH_2Cl_2/ MeOH, 9:1). **^{1}H NMR (CD_2Cl_2, 400 MHz):** δ = 8.85 (s, 1H, CH), 7.83 (d, J^3 = 9.1 Hz, 1H, CH), 7.12 (d, J^3 = 9.3 Hz, 1H, CH), 7.02 (s, 1H, CH), 6.89 (br s, 1H, NH), 6.50 (s, 1H, CH), 6.34 (s, 1H, CH), 5.21 (br s, 1H, NH), 4.60 (t, J^3 = 7.4 Hz, 2H, CH_2), 3.33 (m, 2H, CH_2), 2.03 (m, 2H, CH_2), 1.76 (m, 2H, CH_2), 1.53 (s, 9H, CH_3), 1.47 (m, 4H, CH_2), 1.31 (m, 22H, CH_2), 0.89 (m, 6H, CH_3). **^{13}C{^{1}H} NMR ($CDCl_3$, 101 MHz):** No quaternary C due to relaxation problems. The primary, secondary and tertiary carbon signals were defined and confirmed with **^{13}C{^{1}H} DEPT135 NMR. ^{13}C{^{1}H} NMR ($CDCl_3$, 101 MHz, DEPT135:** δ =131.8 (CH), 119.6 (CH), 101.2 (CH), 90.4 (CH), 77.3 (CH), 72.0 (CH), 47.9 (CH_2), 43.8 (CH_2), 31.8 (2 CH_2), 30.3 (CH_3), 29.7 (CH_2), 29.4 (CH_2), 29.37 (CH_2), 29.32 (CH_2), 29.2 (CH_2), 28.9 (CH_2), 28.7 (CH_3), 27.2 (CH_2), 27.1 (CH_2), 26.8 (CH_2), 22.7 (CH_2), 14.1 (CH_3). **^{19}F (CD_2Cl_2, 376 MHz):** δ = -70.74 (d, J = 720 Hz, PF_6). **HRMS (ESI+)** calculated for [C^+]: 616.3543 ($C_{34}H_{52}Cl_2N_5O^+$), found: 616.3542; **HRMS (ESI-)** calculated for [A^-]: 144.9647 (PF_6^-), found 144.9651.

TAP1b

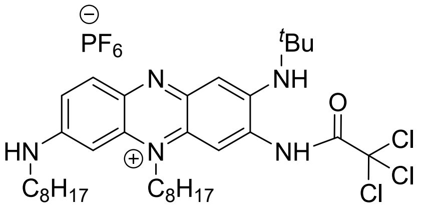

In a 10 mL two-neck flask, pre-dried under vacuum and purged with Argon, 50 mg (0.076 mmol, 1 equiv.) of **TAP** and 14 µL (0.079 mmol, 1.1 equiv.) of *N,N*-diisopropylethylamine were dissolved in 2 mL of anhydrous CH_2Cl_2. An ice bath was prepared to cool the mixture at 0 °C and 17 µL (0.153 mmol, 2 equiv.) of Cl_3COCl were added and a color change of the mixture from purple to blue was noticed. The reaction was stirred at 0 °C for 1 h, then it was taken in 30 mL of CH_2Cl_2, washed with 60 mL of H_2O and with 40 mL of a 0.1 M aqueous solution of KPF_6.The aqueous layers were extracted another time with 10 mL of CH_2Cl_2. The organic layers were collected, dried over anhydrous Na_2SO_4, filtered and the solvent evaporated. The residue was purified by silica gel chromatography, using CH_2Cl_2/MeOH as eluent (gradient from 98:2 to 95:5). The blue fractions were collected to afford the product as a blue solid in 46% yield (29 mg, 0.036 mmol).

R_f: 0.6 (SiO_2, CH_2Cl_2/ MeOH, 95:5). **^{1}H NMR (CD_2Cl_2, 400 MHz):** δ = 8.82 (s, 1H, CH), 7.84 (d, J^3 = 9.0 Hz, 1H, CH), 7.11 (d, J^3 = 9.1 Hz, 1H, CH), 7.02 (s, 1H, CH), 6.50 (s, 1H, CH), 5.09 (br s, 1H, NH), 4.65 (t, J^3 = 7.7 Hz, 2H, CH_2), 3.32 (m, 2H, CH_2), 2.04 (m, 2H, CH_2), 1.76 (m, 2H, CH_2), 1.64 (m, 2H, CH_2), 1.52 (s, 9H, CH_3), 1.47 (m, 4H, CH_2), 1.32 (m, 16H, CH_2), 0.90 (m, 6H, CH_3). **^{13}C{^{1}H} NMR ($CDCl_3$, 101 MHz):** δ = 167.8 (C), 151.3 (C), 145.2 (C), 141.7 (C), 137 (C), 132 (CH), 131.5 (C), 130.8 (2C), 120.6 (CH), 101.4 (CH), 99.2 (C), 90.2 (CH), 51.1 (C), 48.2 (CH_2), 44 (CH_2), 32.1 (CH_2), 29.6 (CH_2), 29.5 (CH_2), 29.1 (CH_2), 29.04 (CH_2), 28.96 (CH_2), 27.57 (CH_2), 27.52 (CH_2), 27.4 (CH_2), 27.2 (CH_2), 27.1 (CH_2), 22.9 (CH_2), 14.4 (CH_3), 14.3 (CH_3). One CH signal is overlapping with the solvent peak (confirmed by ^{13}C{^{1}H} DEPT 135 spectrum, see Figure S 15, Figure S 16). **^{19}F ($CDCl_3$, 376 MHz):** δ = -71.11 (d, J = 706 Hz, PF_6). **HRMS (ESI+)** calculated for [C^+]: 652.3131 ($C_{34}H_{51}Cl_3N_5O^+$), found: 652.3131; **HRMS (ESI-)** calculated for [A^-]: 144.9647 (PF_6^-), found 144.9650.

TAP1c

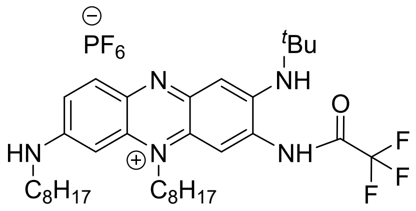

In a two-neck flask, pre-dried under vacuum and purged with Argon, a solution of trifluoroacetic anhydride (42 µL, 0.3 mmol, 1 equiv.) in 5 mL of anhydrous CH_2Cl_2 was mixed with 59 µL of *N,N*-diisopropylethylamine (0.33 mmol, 1.1 equiv.) and the mixture was cooled to -15 °C. A solution of 200 mg (0.3 mmol, 1 equiv.) of **TAP** in 25 mL of anhydrous CH_2Cl_2 was prepared and added dropwise to the cold solution. The reaction was stirred at -15 °C for 15-20 min, then it was allowed to warm to room temperature and stirred at 25 °C for 3 h. Another 84 µL (0.6 mmol, 2 equiv.) of trifluoroacetic anhydride and 59 µL (0.33 mmol, 1.1 equiv.) of *N,N*-diisopropylethylamine were added and the reaction was stirred at 25 °C for another 16 h. Then, the mixture was taken in 50 mL of CH_2Cl_2, washed with 100 mL of H_2O, then with 100 mL of an 0.1 M aqueous solution of KPF_6. The aqueous layers were extracted another time with 30 mL of CH_2Cl_2. The organic layers were collected, dried over anhydrous Na_2SO_4, filtered and the solvent was evaporated. The residue was further purified on silica gel chromatography, using CH_2Cl_2/MeOH as eluent (95:5). The turquoise fractions were collected and subjected to a second silica gel column chromatography, using AcOEt/petroleum ether (7:3) as eluent to afford the product as a blue solid in 6% yield (14 mg, 0.018 mmol).

R_f: 0.7 (SiO_2, AcOEt/petroleum ether, 7:3).**^{1}H NMR ($CDCl_3$, 400 MHz):** δ = 8.86 (s, 1H, CH), 7.77 (d, J^3 = 9.6 Hz, 1H, CH), 7.10 (dd, J^3 = 9.1 Hz, J^4 = 1.8 Hz, 1H, CH), 7.03 (s, 1H, CH), 6.31 (d, J^4 = 1.8 Hz 1H, CH), 5.37 (br s, 1H, NH), 4.53 (t, J^3 = 7.0 Hz, 2H, CH_2), 3.25 (m, 2H, CH_2), 1.99 (m, 2H, CH_2), 1.76 (m, 2H, CH_2), 1.61 (m, 2H, CH_2), 1.52 (s, 9H, CH_3), 1.29 (m, 18H, CH_2), 0.89 (m, 6H, CH_3). **$^{13}C\{^1H\}$ NMR ($CDCl_3$, 101 MHz):** δ = 163.2 (C, $J^2_{C\text{-}F}$ = 33.5 Hz, CO), 154.4 (C), 151.5 (C), 144.38 (C), 141.1 (C), 137.3 (C), 131.8 (CH), 131.4 (C), 130.0 (C), 120.9 (CH), 119.2 (C, $J^1_{C\text{-}F}$ = 291.3 Hz), 102.6 (CH), 102.2 (CH), 89.6 (CH), 51.0 (C, ^{t}Bu), 48 (CH_2), 43.8 (CH_2), 31.9 (CH_2), 31.8 (CH_2), 29.8 (CH_2), 29.5 (CH_2), 29.3 (CH_2), 29.2 (CH_2), 28.87 (CH_2), 28.85 (CH_3), 27.3 (CH_2), 27.1 (CH_2), 26.9 (CH_2), 22.77 (CH_2), 22.74 (CH_2), 14.3 (CH_3). **^{19}F ($CDCl_3$, 376 MHz):** δ = -70.68 (d, *J* = 719 Hz, PF_6), -75.41 (CF_3). **HRMS (ESI+)** calculated for the agglomerate [(C^+A^-)C^+]: 1349.7728 ($C_{68}H_{102}F_{12}N_{10}O_2P^+$), found: 1349.7745.

TAP2

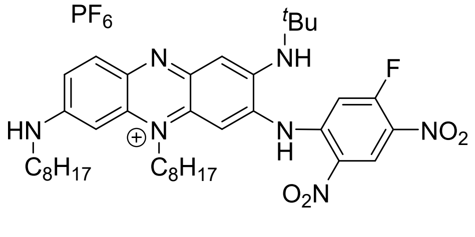

In a 100 mL two-neck flask, pre-dried under vacuum and purged with Argon, 250 mg (0.38 mmol, 1 equiv.) of **TAP** and 0.13 mL (0.76 mmol, 2 equiv.) of *N,N*-diisopropylethylamine were dissolved in 50 mL of anhydrous CH_2Cl_2. After that, 86 mg of 1,5-difluoro-2,4-dinitrobenzene (0.42 mmol, 1.1 equiv.) were added and the reaction was stirred at 25 °C for 48 h. Then, the reaction was taken in 50 mL of CH_2Cl_2, washed with

100 mL H_2O then with 100 mL of a 0.1 M aqueous solution of KPF_6. The aqueous layers were extracted another time with 30 mL of CH_2Cl_2. The organic layers were collected, dried over anhydrous Na_2SO_4, filtered and the solvent was evaporated. The residue was purified twice on silica gel chromatography, using CH_2Cl_2/MeOH as eluent (95:5). The turquoise fractions were collected to afford the product as a blue solid in 68% yield (217 mg, 0.259 mmol).

Alternatively, **TAP2** can be isolated in a microwave reaction with the following protocol. In a microwave adapted flask, **TAP** (15 mg, 0.022 mmol, 1 equiv.) and 1,5-difluoro-2,4-dinitrobenzene (5.2 mg, 0.024 mmol, 1.1 equiv.) were dissolved in 1.5 mL of anhydrous acetonitrile. *N,N*-Diisopropylethylamine (0.1 mL, excess.) was added and the mixture was purged with Argon. The mixture was introduced was heated for 30 min at 80 °C at fixed power of 200 W. The residue was purified on silica gel chromatography, using CH_2Cl_2/MeOH as eluent (95:5) to give 20 mg of product (0.017 mmol, 80% yield).

R$_f$: 0.65 (SiO_2, CH_2Cl_2/MeOH, 95:5). **^{1}H NMR ($CDCl_3$, 400 MHz):** δ = 8.88 (d, $J^4_{CH\text{-}F}$ = 7.9 Hz, 1H, CH), 7.75 (d, J^3 = 8.6 Hz, 1H, CH), 7.11 (d, $J^3_{CH\text{-}F}$ = 13 Hz, 1H, CH), 6.83 (d, J^3 = 8.5 Hz, 1H, CH), 6.79 (s, 1H, CH), 6.38 (s, 1H, CH), 6.30 (s, 1H, CH), 6.22 (br s, 1H, NH), 4.46 (br s, 1H, NH), 4.17 (t, J^3 = 8.0 Hz, 2H, CH_2), 3.25 (t, J^3 = 7.4 Hz, 2H, CH_2), 1.86 (m, 2H, CH_2), 1.74 (m, 2H, CH_2), 1.52 (s, 9H, CH_3), 1.46 (m, 4H, CH_2), 1.33 (m, 16H, CH_2), 0.89 (m, 6H, CH_3). **^{13}C{^{1}H} NMR ($CDCl_3$, 101 MHz):** δ = 160.0 (C, $J^1_{C\text{-}F}$ = 269.4 Hz), 153.9 (C), 153.8 (C, $J^2_{C\text{-}F}$ = 12.6 Hz), 150.0 (C), 143.3 (C), 143.2 (C), 137.8 (C), 132.8 (C), 132.4 (C), 131.2 (CH), 128.1 (C), 128.0 (C), 125.9 (CH), 115.4 (CH), 110.1 (CH, $J^2_{C\text{-}F}$ = 22.9 Hz), 101.3 (CH), 91.4 (CH), 89.2 (CH), 51.1 (C, ^{t}Bu), 46.7 (CH_2), 43.8 (CH_2), 31.9 (CH_2), 31.8 (CH_2), 29.5 (CH_2), 29.37 (CH_2), 29.35 (CH_2), 29.29 (CH_2), 29.27 (CH_2), 28.9 (CH_3), 27.3 (CH_2), 27.1 (CH_2), 26.4 (CH_2), 22.78 (CH_2), 22.71 (CH_2), 14.18 (CH_3), 14.12 (CH_3). **^{19}F ($CDCl_3$, 376 MHz):** δ = -70.95 (d, *J* = 721 Hz, PF_6), -108.19 (F). **HRMS (ESI+)** calculated for the agglomerate [(C^+A^-)C^+]: 1525.7922 ($C_{76}H_{106}F_8N_{14}O_8P^+$), found: 1525.7915.

TAP3a

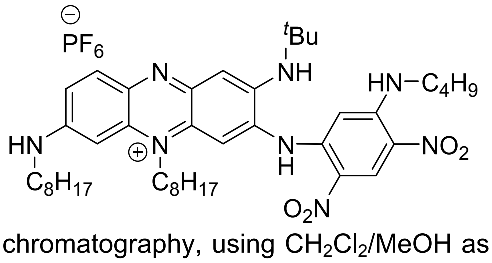

In round-bottom flask, 35 mg of **TAP2** (0.042 mmol, 1 equiv.) were dissolved in 15 mL of MeCN and 16 µL of *n*-butylamine (0.167 mmol, 4 equiv.) were added. The reaction was stirred at 25 °C for 16 h. Then the solvent was evaporated and the residue was purified on silica gel chromatography, using CH_2Cl_2/MeOH as eluent (95:5) to afford the product as a purple solid in 90% yield (34 mg, 0.038 mmol).

R$_f$: 0.4 (SiO_2, CH_2Cl_2/MeOH, 95:5).**^{1}H NMR ($CDCl_3$, 400 MHz):** δ = 9.12 (s, 1H, CH), 8.31 (s, 1H, CH), 7.64 (d, J^3 = 9.0 Hz, 1H, CH), 6.72 (s, br, 2H, 2CH), 6.30 (s, 1H, CH), 6.26 (s, 1H, CH), 6.09 (s, br, H, NH), 5.93 (s, br, H, NH), 4.00 (br s, 2H, CH_2), 3.25 (m, 4H, CH_2), 1.70 (m, 6H, CH_2), 1.51 (s, 9H, CH_3), 1.45 (m, 4H, CH_2), 1.27 (m, 20H, CH_2), 0.95 (m, 3H, CH_3), 0.87 (m, 6H, CH_3). **^{13}C{^{1}H} NMR ($C_4D_2Cl_4$, 125 MHz, 343 K):** δ = 148.6 (C), 141.9 (C), 132.9 (C), 128.3 (CH), 126.4 (CH), 90.3 (CH), 51.9 (N-

CH_2), 51.7 (CH_2), 47.4 (CH_2), 44.3 (CH_2), 43.3 (CH_2), 31.8 (CH_2), 31.6 (CH_2), 30.7 (CH_2), 29.7 (CH_2), 29.2 (C, ^{t}Bu), 27.2 (CH_2), 26.9 (CH_2), 22.6 (CH_2), 22.5 (CH_2), 20.1 (CH_2), 14.0 (CH_3), 13.9 (CH_3), 13.6 (CH_3). Poorly resolved spectra didn't allow the identification of all the aromatic carbons (4 CH and 7 C missing). **^{19}F ($CDCl_3$, 376 MHz):** δ = -71.26 (d, J = 722 Hz, PF_6). **HRMS (ESI+)** calculated for [C^+]: 743.4967 ($C_{42}H_{63}N_8O_4^+$), found: 743.4974.

TAP3b

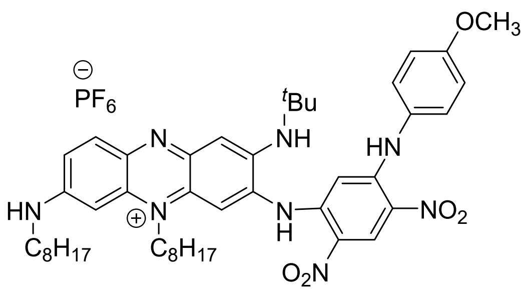

In round-bottom flask, 55 mg of **TAP2** (0.065 mmol, 1 equiv.) were dissolved in 25 mL of MeCN and 32 mg of *p*-anisidine (0.26 mmol, 4 equiv.) were added at 25 °C. The reaction was refluxed for 48 h. Then the solvent was evaporated and the residue was purified on silica gel chromatography, using CH_2Cl_2/AcOEt as eluent (8:2) to afford the product as a purple solid in 91% yield (56 mg, 0.059 mmol).

R_f: 0.2 (SiO_2, CH_2Cl_2/AcOEt, 8:2).**^{1}H NMR ($CDCl_3$, 400 MHz):** δ = 9.74 (s, 1H, CH), 9.13 (s, 1H, CH), 7.63 (d, J^3 = 8.9 Hz, 1H, CH), 7.19 (d, J^3 = 8.7 Hz, 1H, CH), 6.91 (d, J^3 = 8.9 Hz, 2H, CH), 6.70 (d, J^3 = 7.7 Hz, 1H, CH), 6.61 (s, 1H, CH), 6.51 (s, 1H, CH), 6.27 (s, 1H, CH), 6.11 (br s, 1H, NH), 5.75 (br s, 1H, NH), 4.28 (br s, 1H, NH), 3.97 (s, 2H, CH_2), 3.77 (s, 3H, CH_3), 3.20 (m, 2H, CH_2), 1.70 (m, 6H, CH_2), 1.48 (s, 9H, CH_3), 1.29 (m, 4H, CH_2), 1.27 (m, 16H, CH_2), 0.90 (m, 6H, CH_3). **^{13}C{^{1}H} NMR ($CDCl_3$, 125 MHz):** δ = 158.4 (C), 154.3 (C), 149.4 (C), 149.2 (C), 147.4 (C), 144.1 (C), 143.0 (C), 133.1 (C), 132.8 (C), 131.8 (C), 130.9 (C), 130.5 (CH), 130.0 (C), 128.8 (C), 127.2 (CH), 126.2 (CH), 115.1 (CH), 113.0 (CH), 106.0 (CH), 100.5 (CH), 92.0 (CH), 87.9 (CH), 55.5 (O-CH_3), 50.9 (C, ^{t}Bu), 46.1 (CH_2), 43.8 (CH_2), 31.9 (CH_2), 31.8 (CH_2), 31.7 (CH_2), 29.7 (CH_2), 29.4 (CH_2), 29.3 (CH_2), 29.2 (CH_2), 28.9 (CH_2), 27.2 (CH_2), 27.0 (CH_2), 25.4 (CH_2), 22.66 (CH_2), 22.64 (CH_2), 14.1 (CH_3), 14.0 (CH_3). **^{19}F ($CDCl_3$, 376 MHz):** δ = -70.87 (d, J = 711 Hz, PF_6). **HRMS (ESI+)** calculated for [C^+]: 793.4759 ($C_{45}H_{61}N_8O_5^+$), found: 793.4764.

IV. NMR SPECTRA

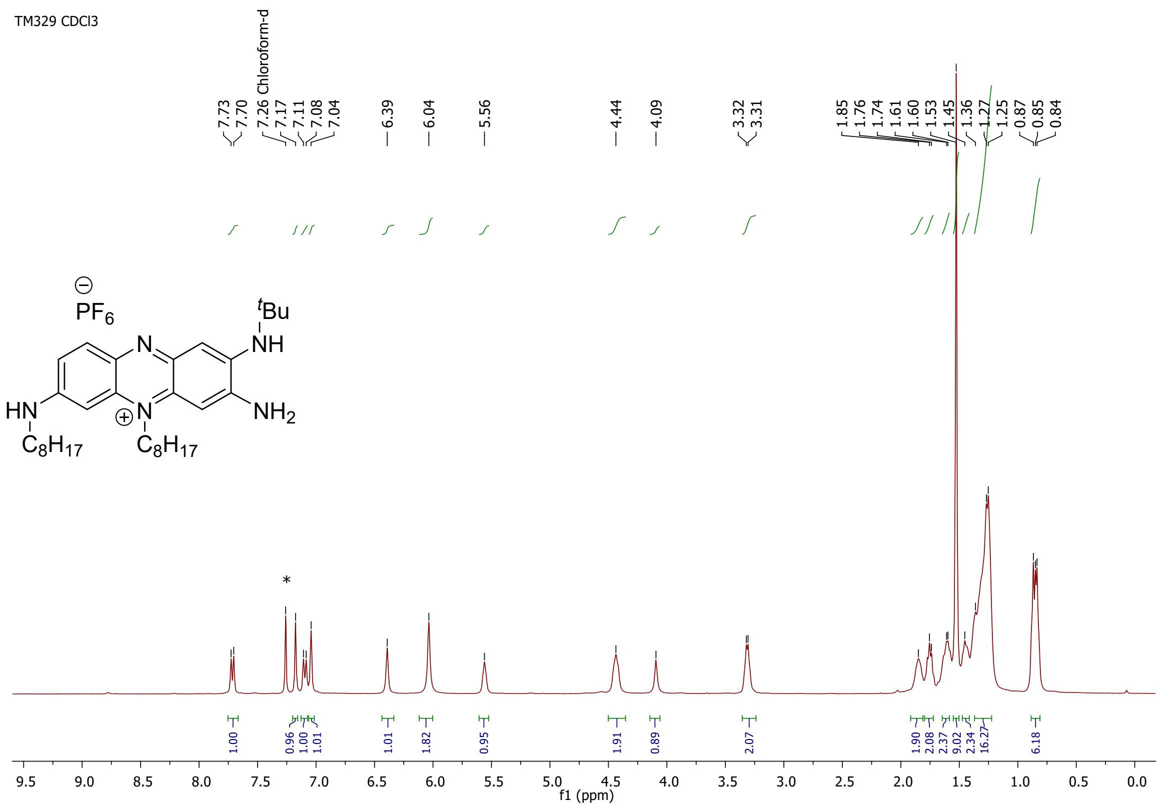

Figure S 10. ^1H NMR (400 MHz, $CDCl_3$) of **TAP.**

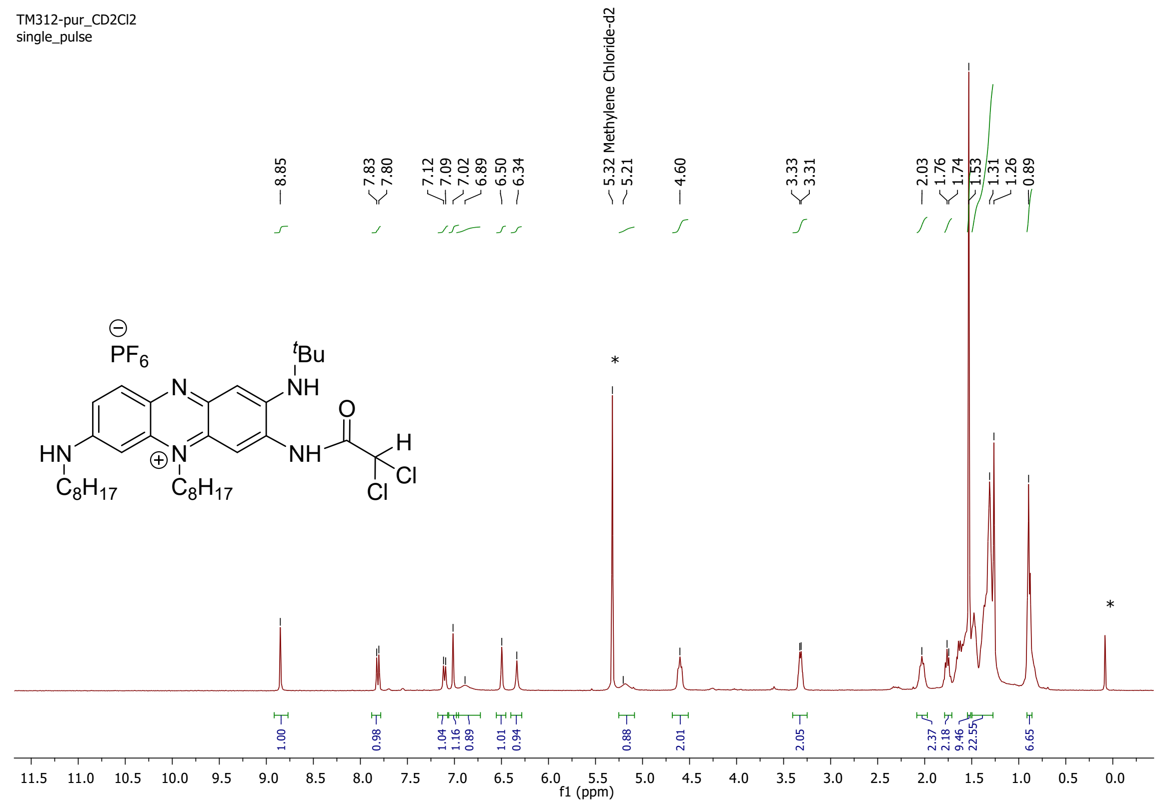

Figure S 11. ^1H NMR (400 MHz, CD_2Cl_2) of **TAP1a**.

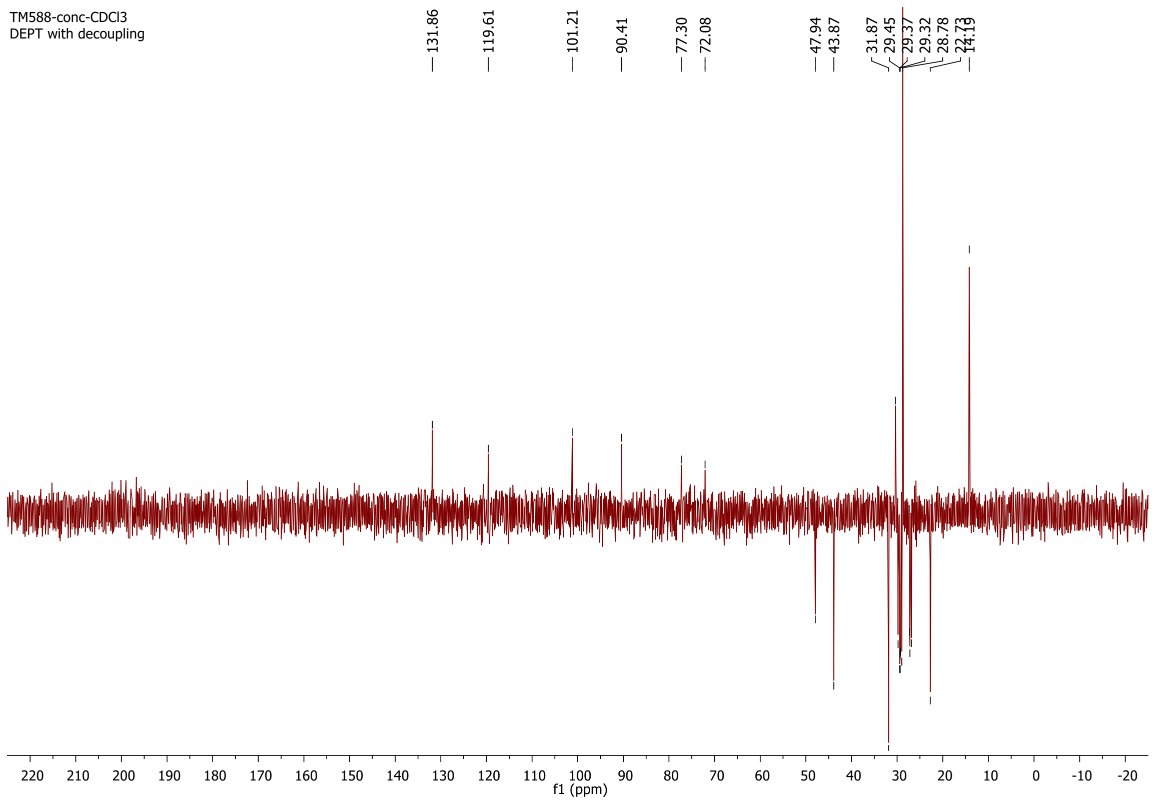

Figure S 12. $^{13}C\{^{1}H\}$ NMR (101 MHz, $CDCl_3$, DEPT135) of **TAP1a**.

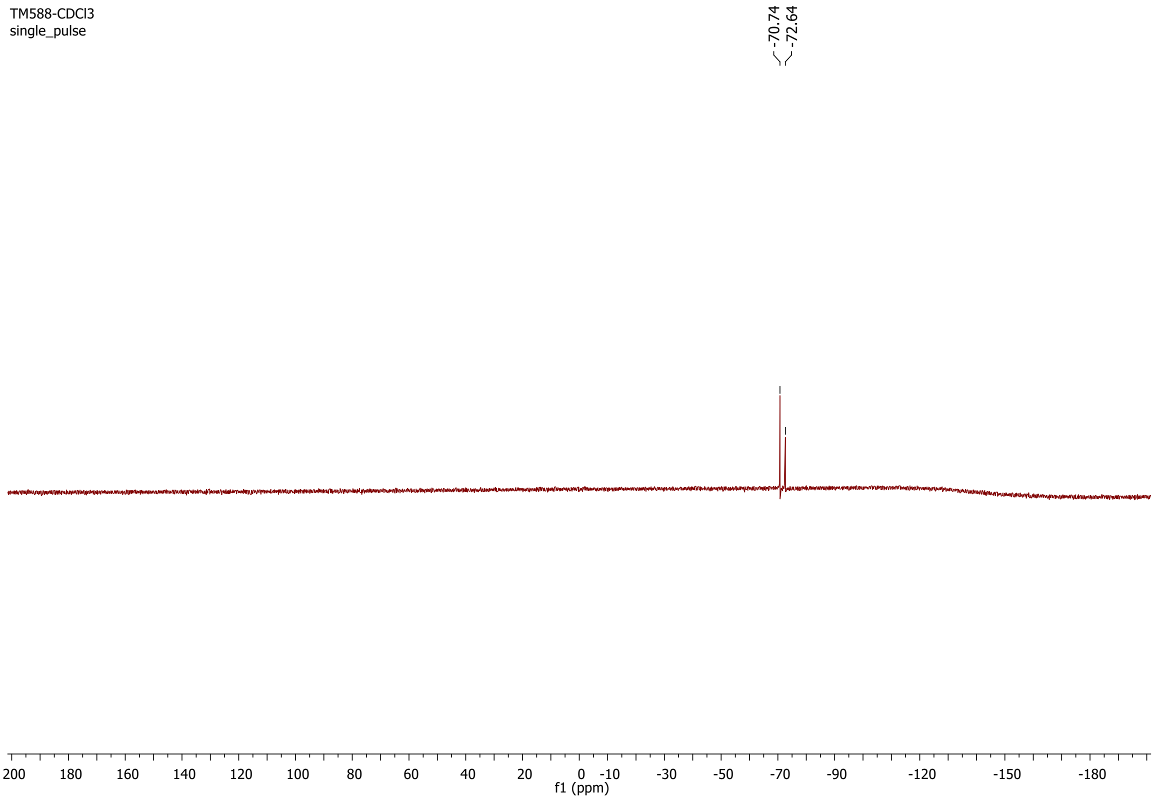

Figure S 13. ^{19}F NMR (376 MHz, $CDCl_3$) of **TAP1a**.

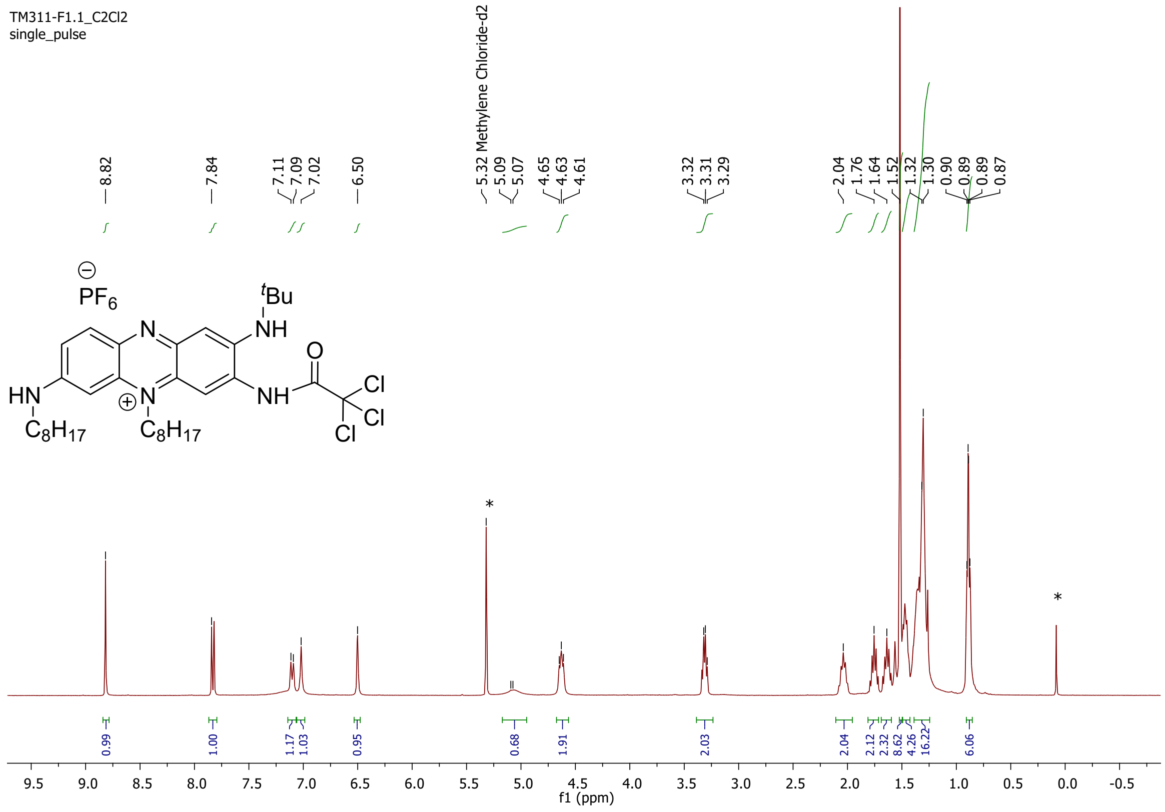

Figure S 14. ^{1}H NMR (400 MHz, CD_2Cl_2) of **TAP1b**.

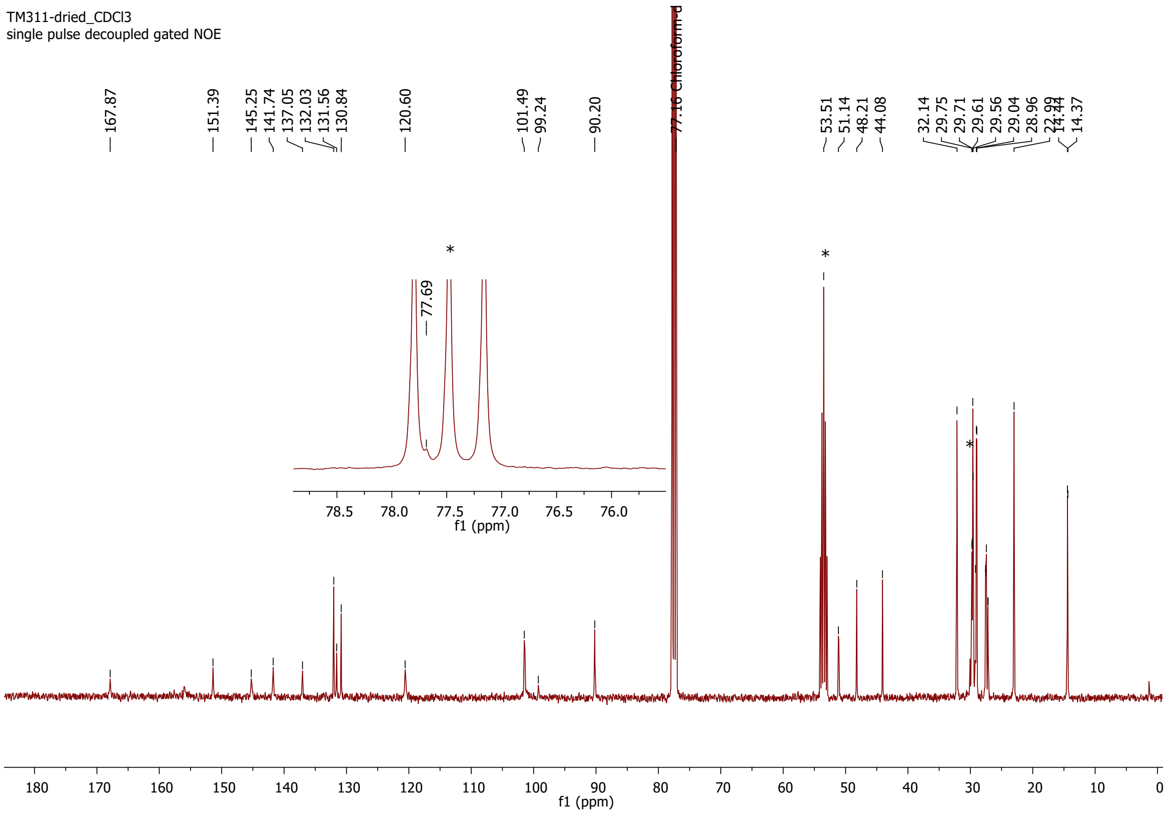

Figure S 15. ^{13}C NMR (101 MHz, $CDCl_3$) of **TAP1b**. Zoom on the residual solvent peak to highlight the overlapped CH signal.

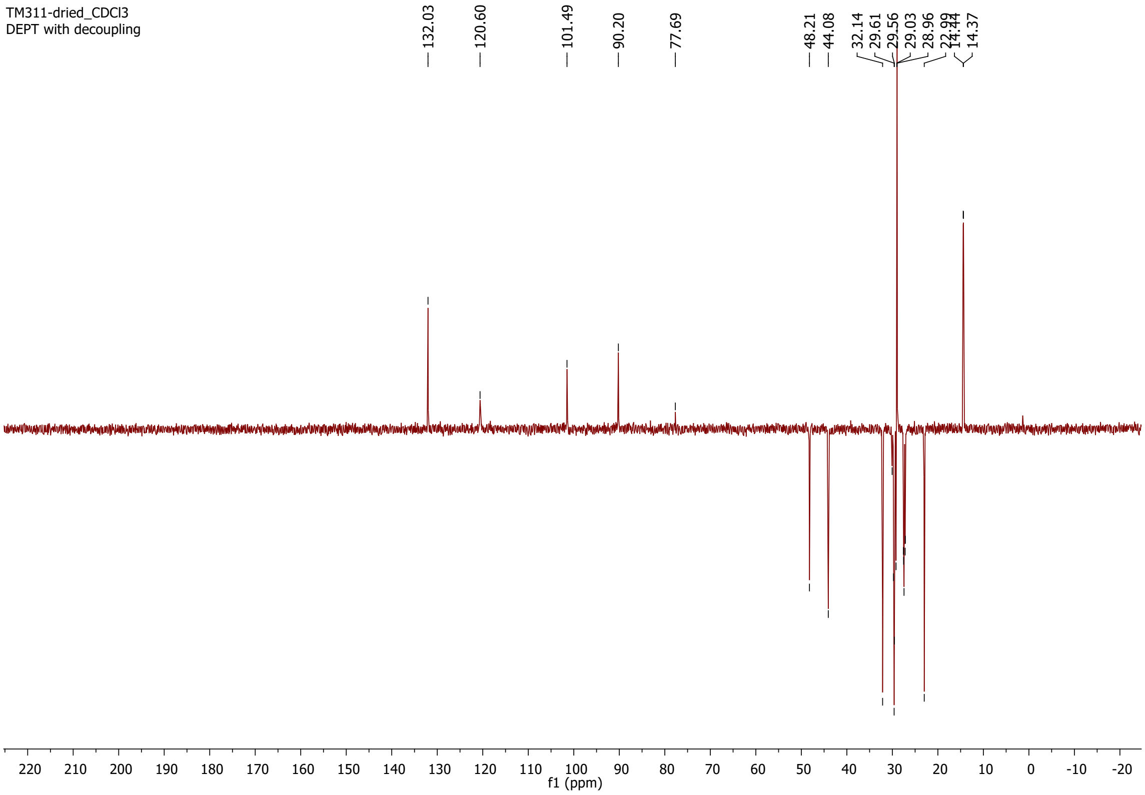

Figure S 16. $^{13}C\{^{1}H\}$ NMR (101 MHz, $CDCl_3$, DEPT135) of **TAP1b**.

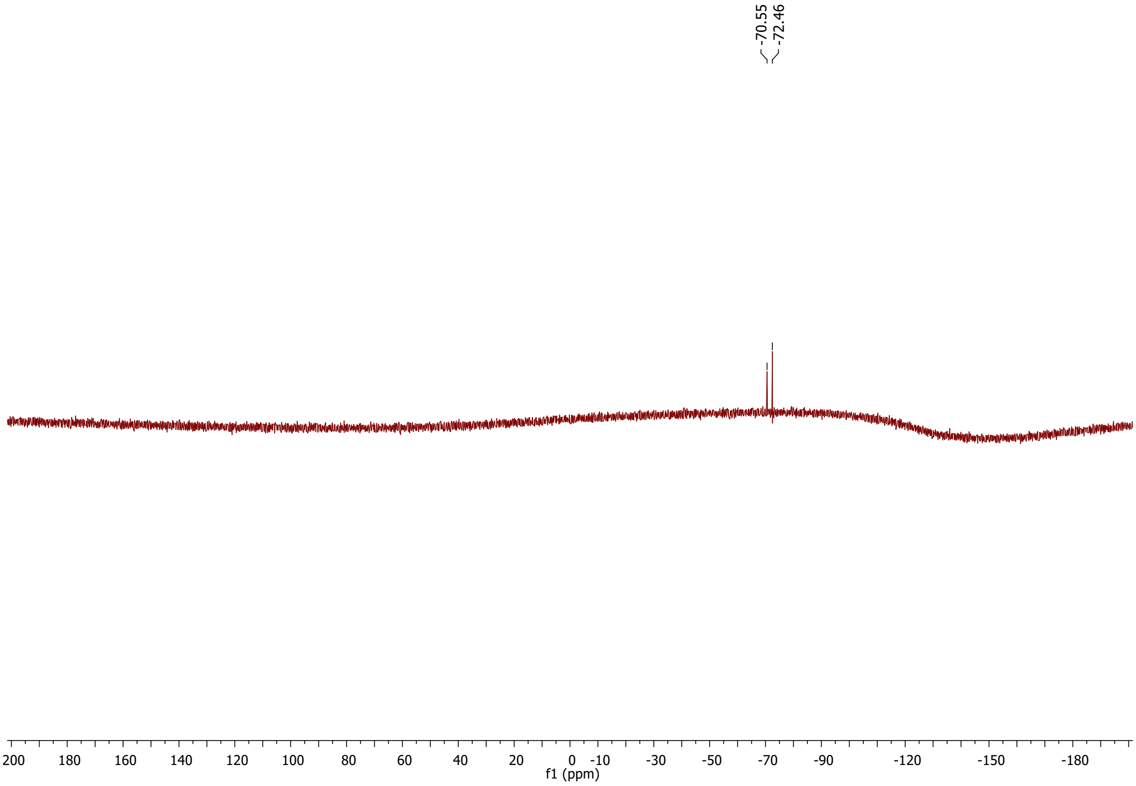

Figure S 17. ^{19}F NMR (376 MHz, $CDCl_3$) of **TAP1b**.

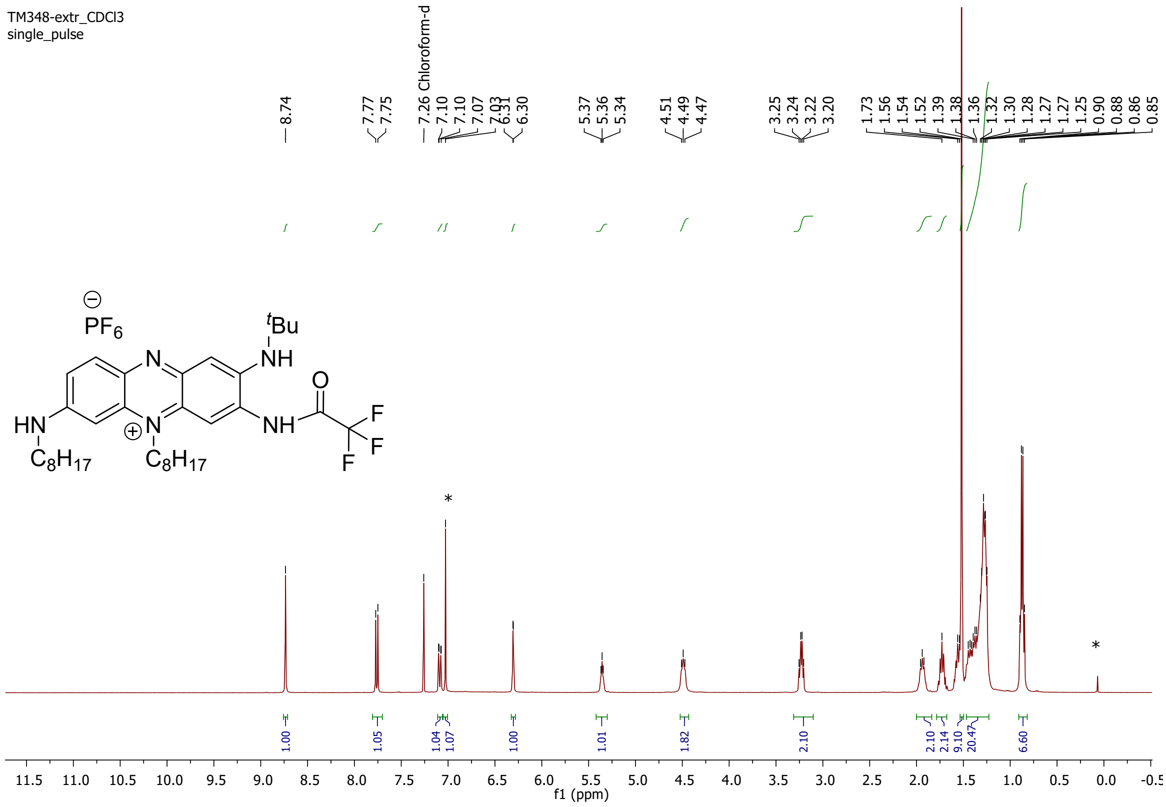

Figure S 18. ^{1}H NMR (400 MHz, $CDCl_3$) of **TAP1c**.

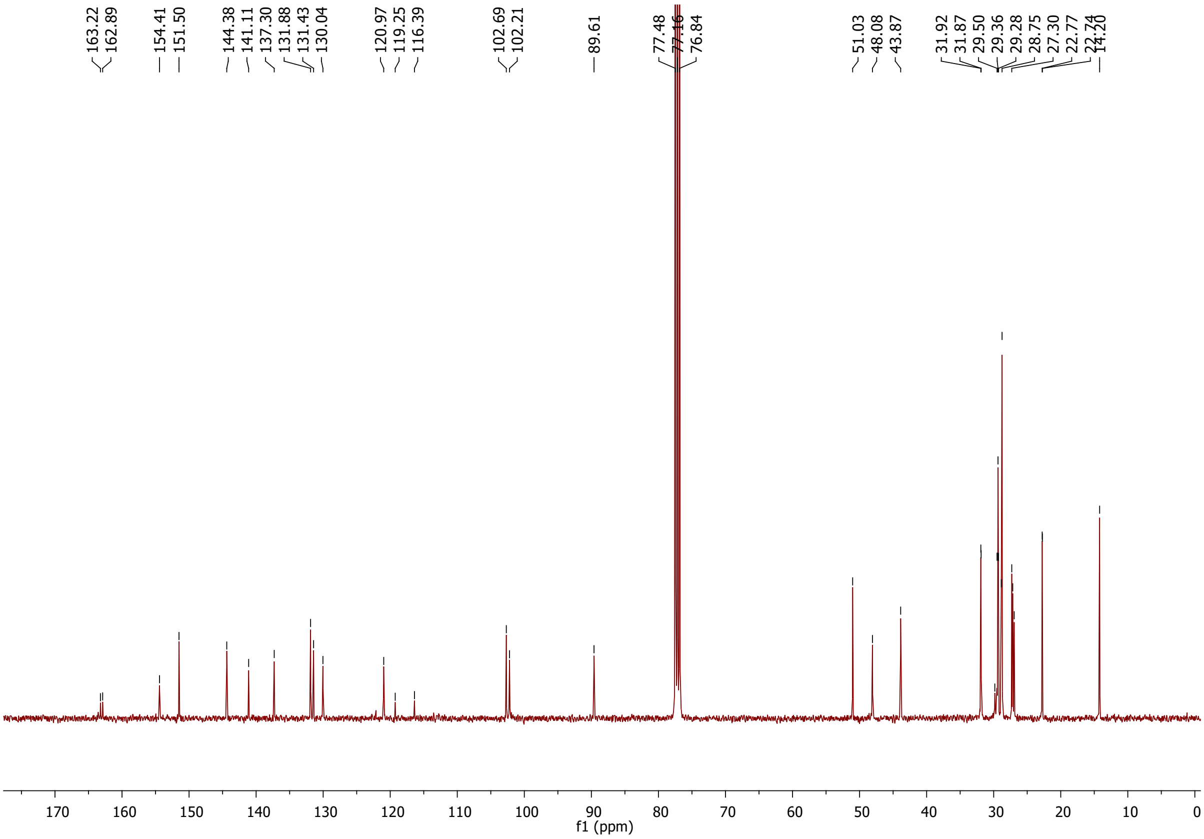

Figure S 19. $^{13}C\{^{1}H\}$ NMR (101 MHz, $CDCl_3$) of **TAP1c**.

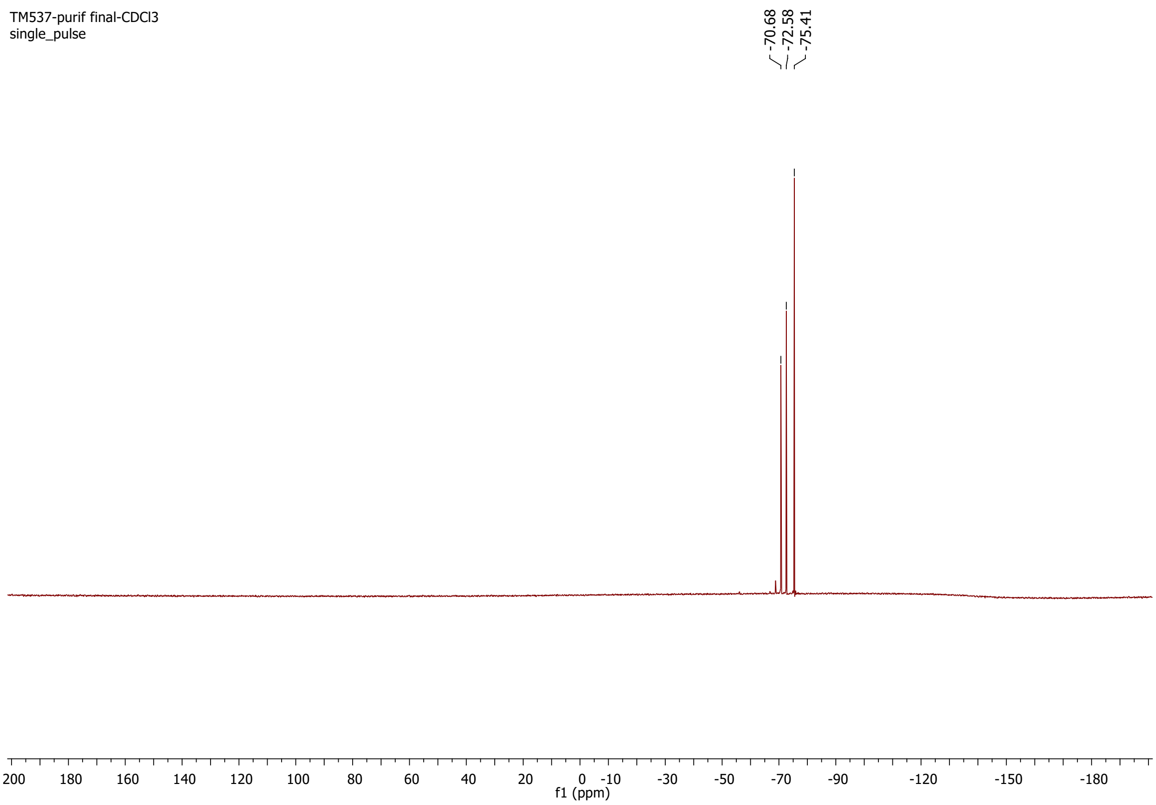

Figure S 20. ^{19}F NMR (376 MHz, $CDCl_3$) of **TAP1c**.

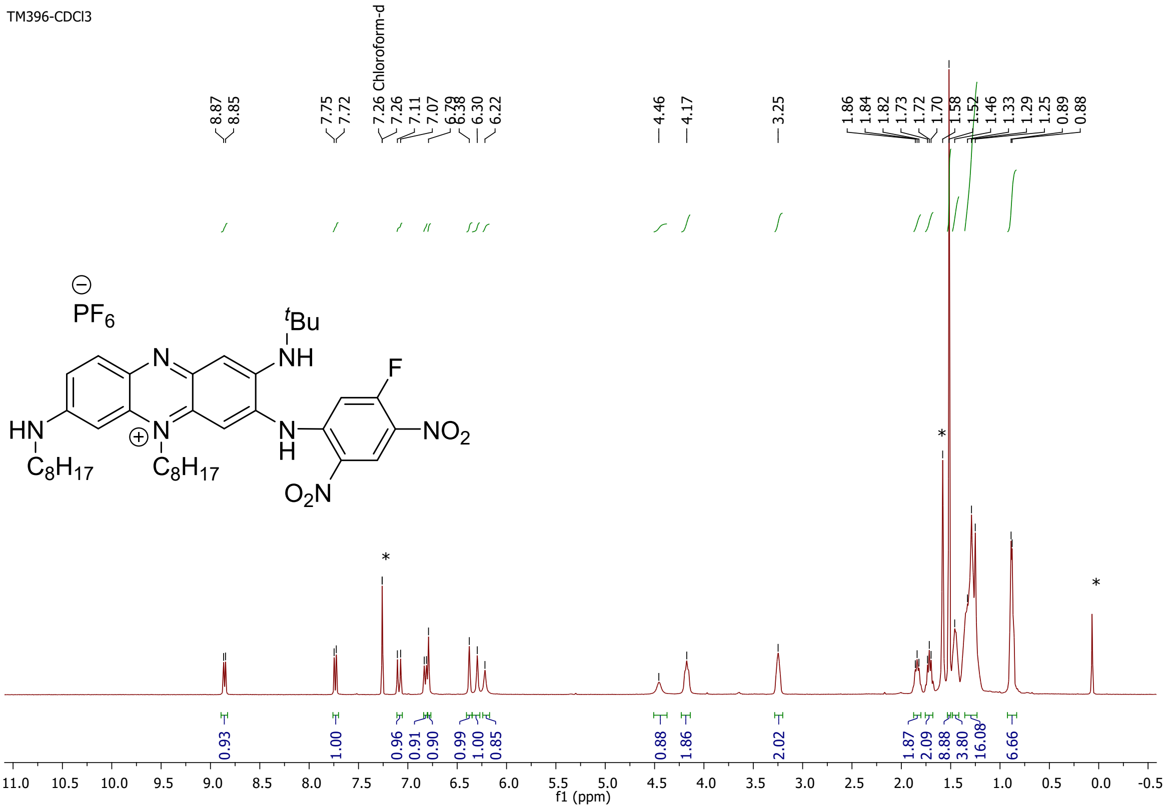

Figure S 21. ^{1}H NMR (400 MHz, $CDCl_3$) of **TAP2**.

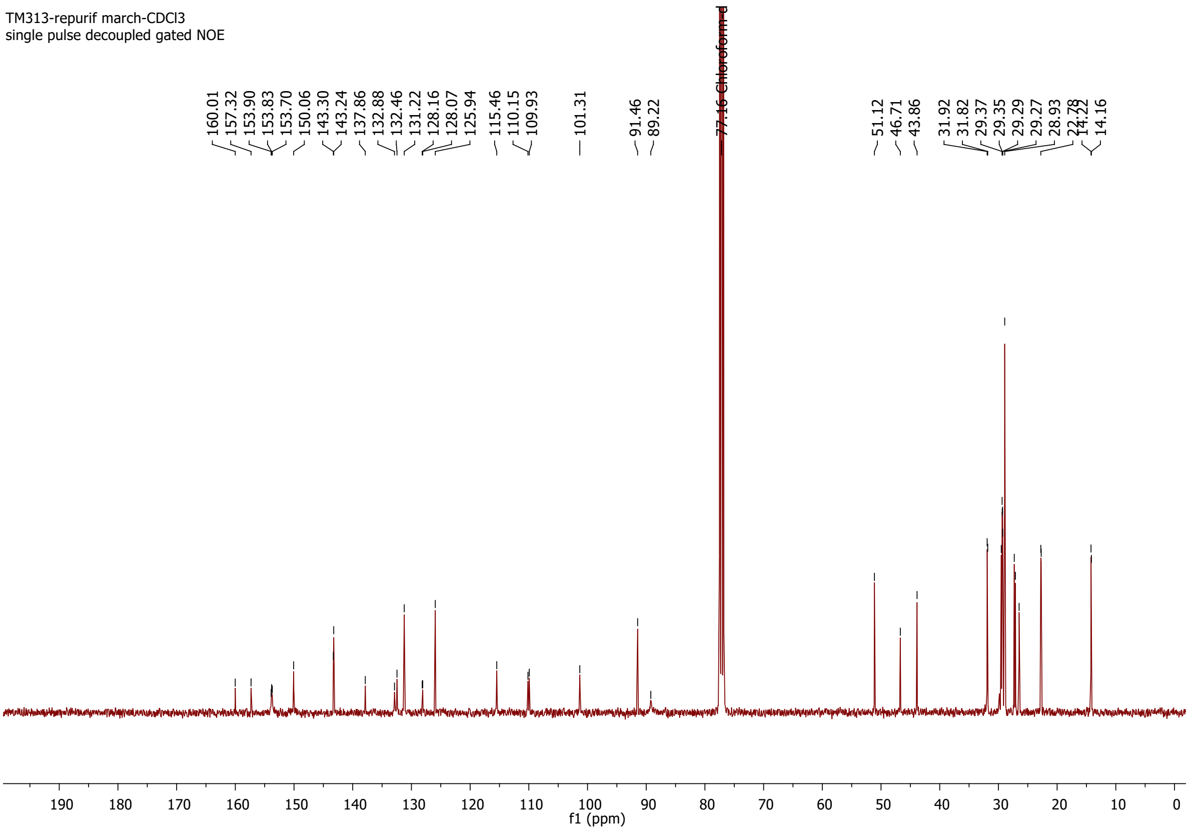

Figure S 22. $^{13}C\{^{1}H\}$ NMR (101 MHz, $CDCl_3$) of **TAP2**.

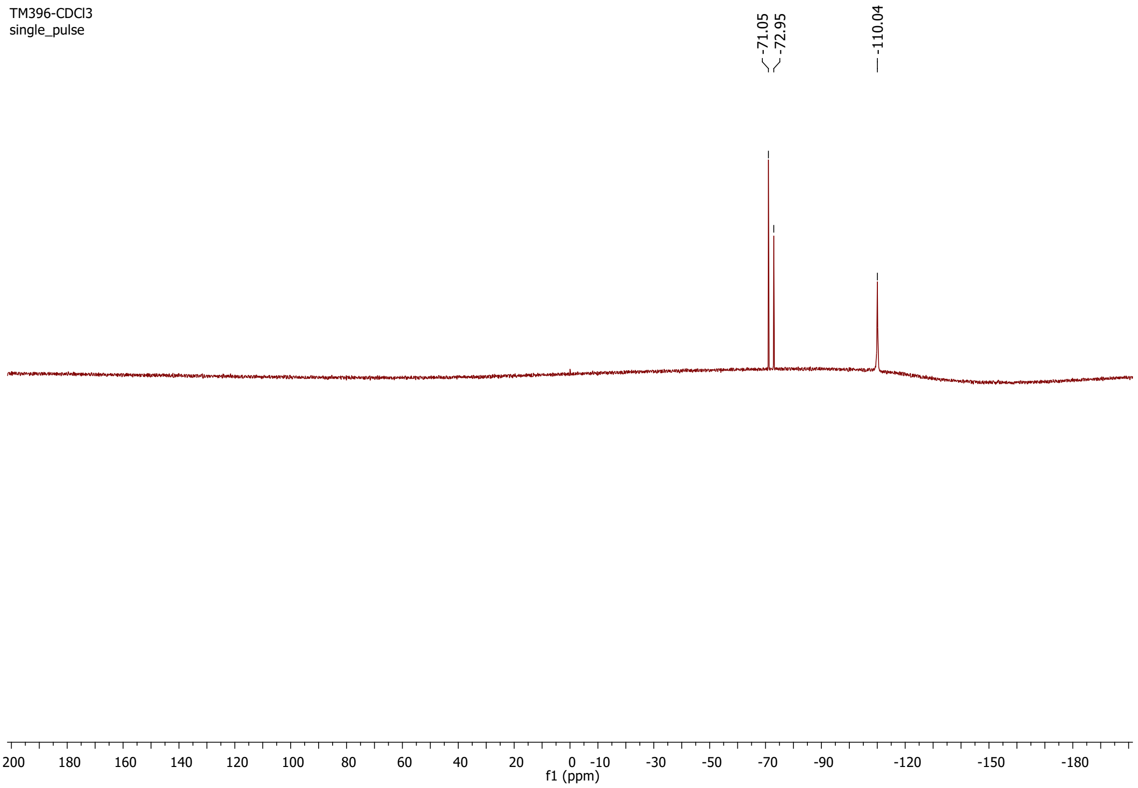

Figure S 23. ^{19}F NMR (376 MHz, $CDCl_3$) of **TAP2**.

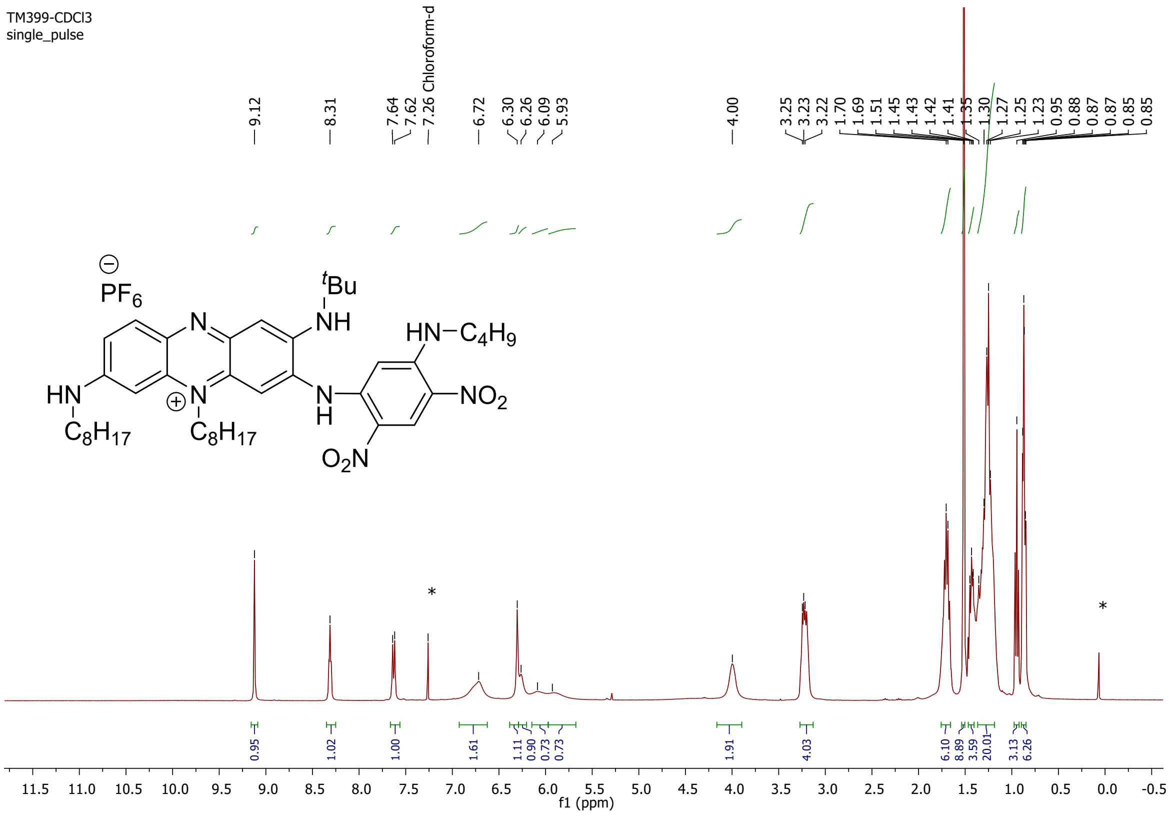

Figure S 24. ^{1}H NMR (400 MHz, $CDCl_3$) of **TAP3a**.

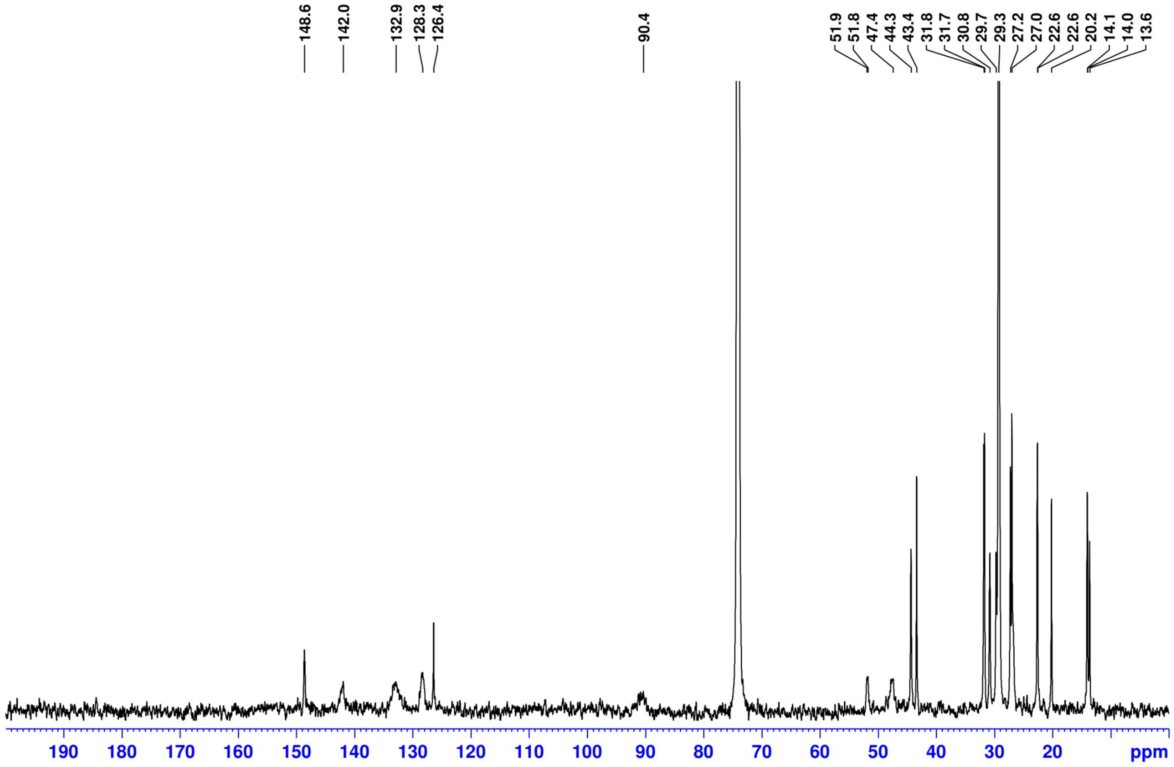

Figure S 25. $^{13}C\{^{1}H\}$ NMR (125 MHz, $C_4D_2Cl_4$, 343 K) of **TAP3a**.

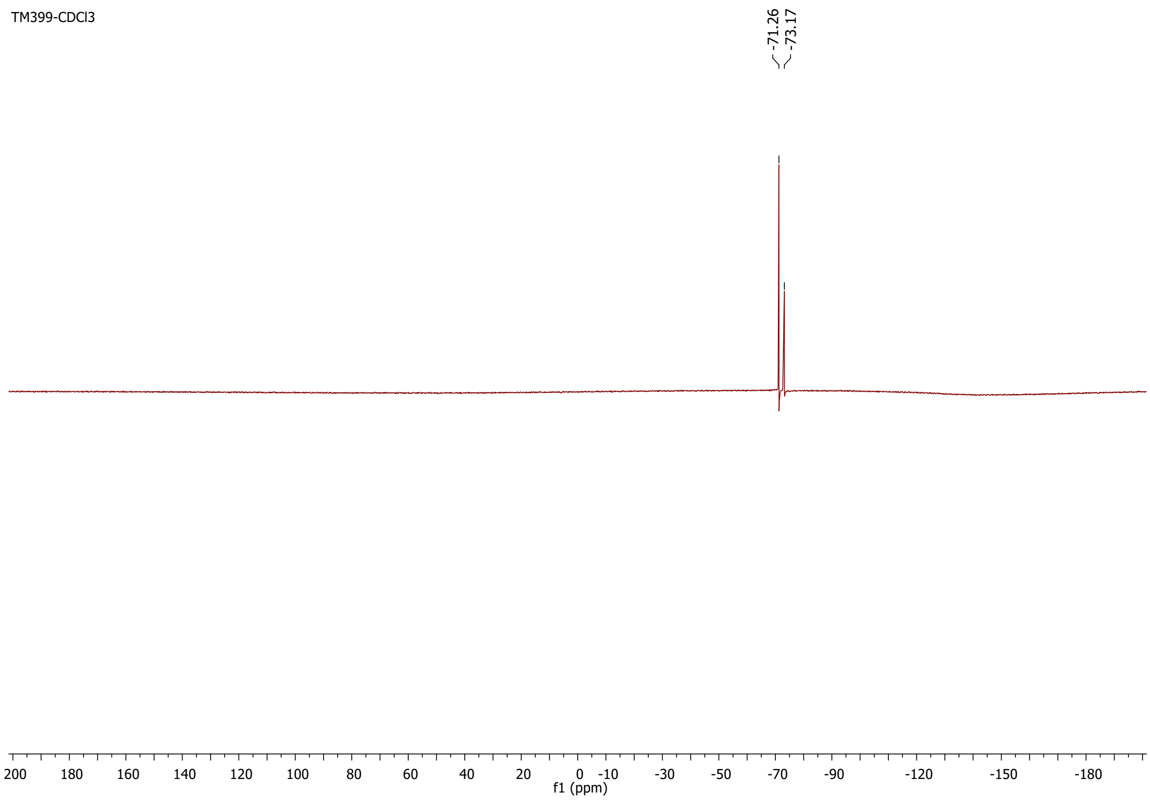

Figure S 26. ^{19}F NMR (376 MHz, $CDCl_3$) of **TAP3a**.

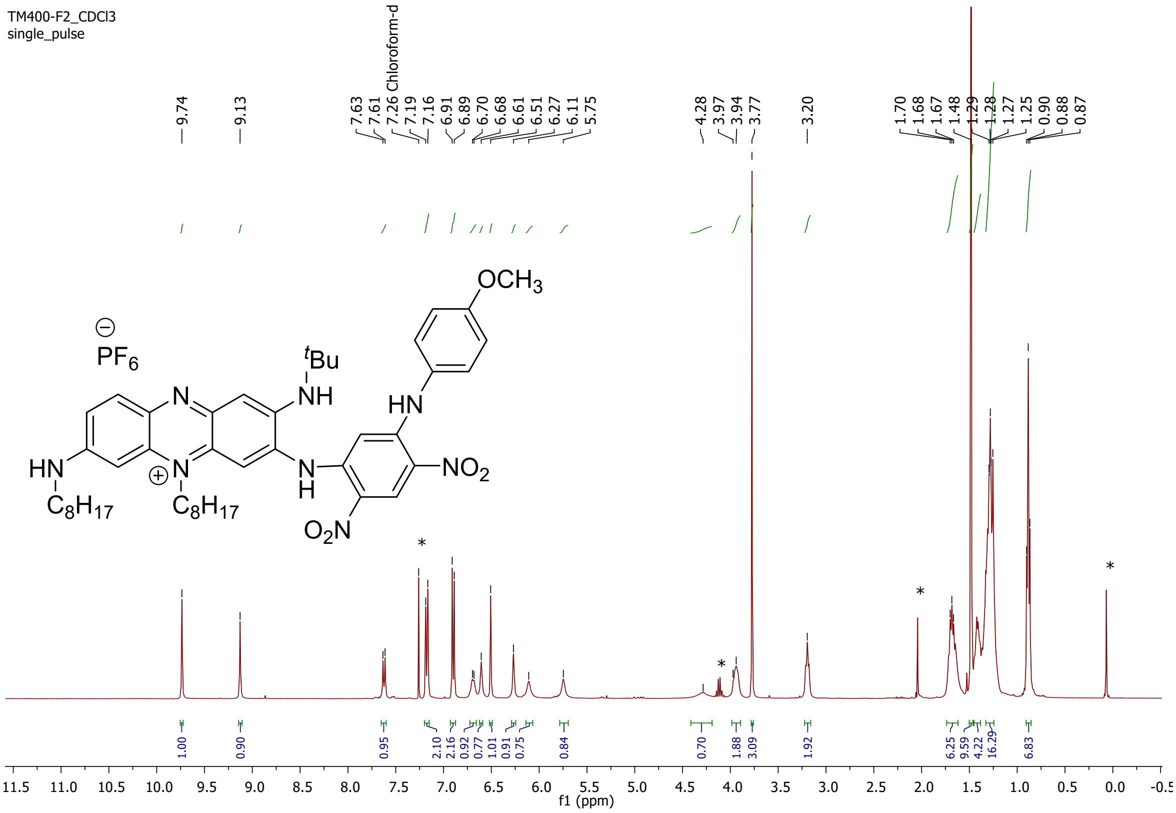

Figure S 27. ^{1}H NMR (400 MHz, $CDCl_3$) of **TAP3b**.

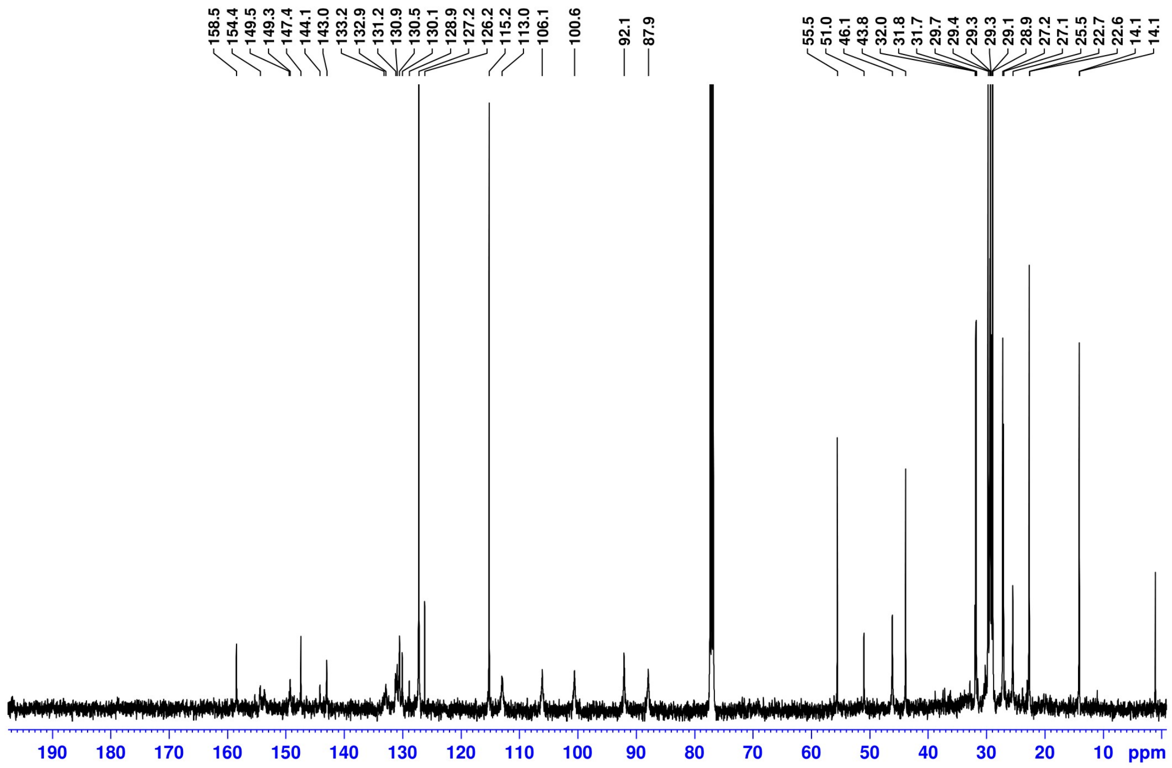

Figure S 28. $^{13}C\{^{1}H\}$ NMR (125 MHz, $CDCl_3$) of **TAP3b**.

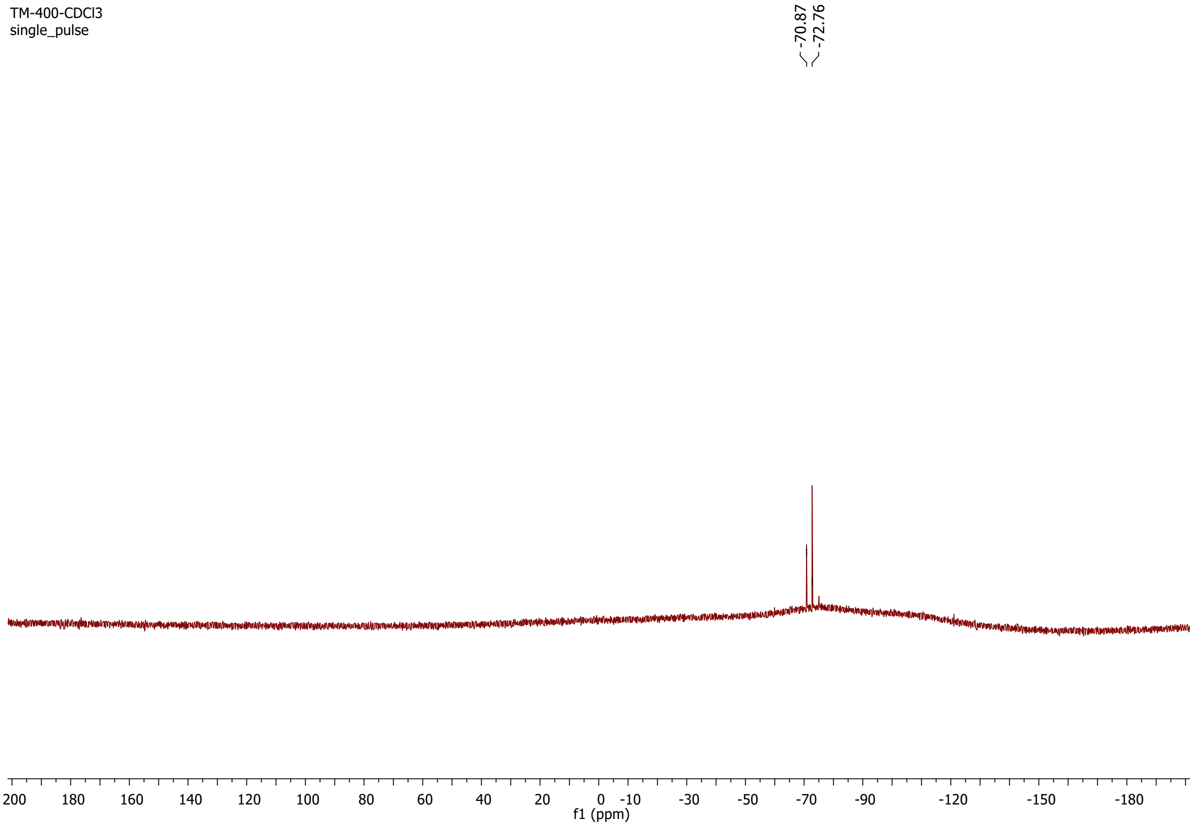

Figure S 29. ^{19}F NMR (376 MHz, $CDCl_3$) of **TAP3b**.

V. MASS SPECTROMETRY

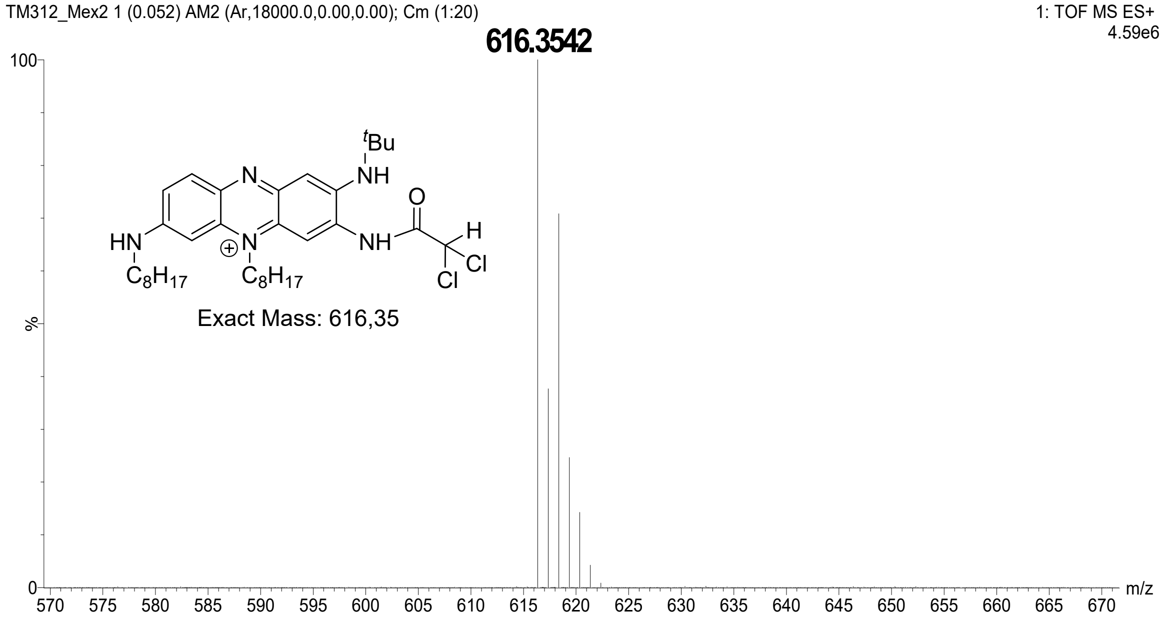

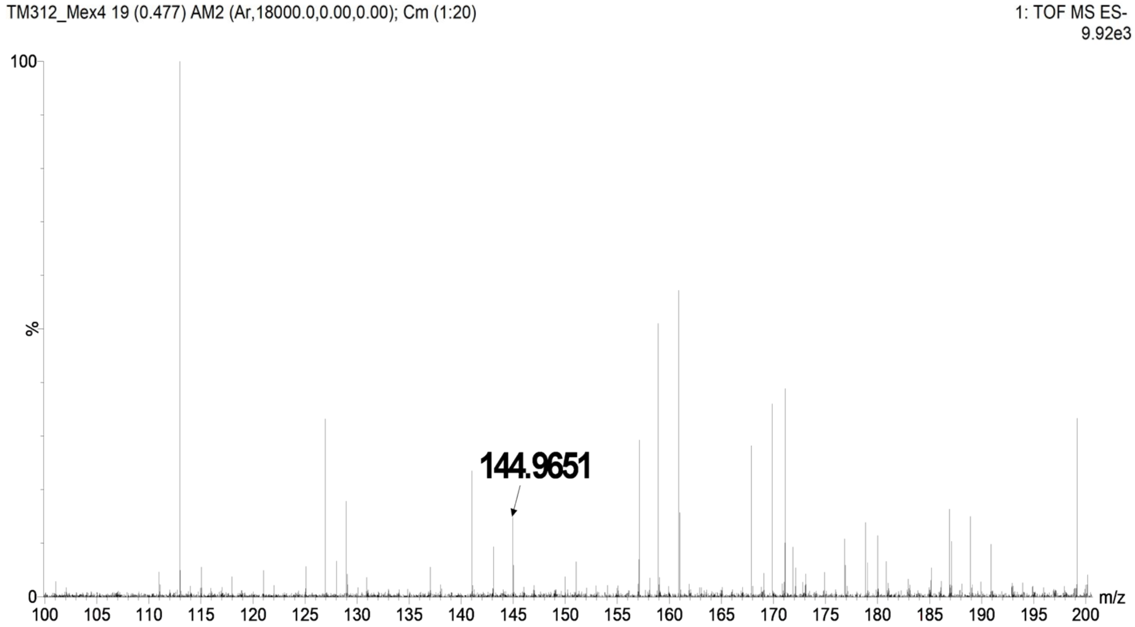

Figure S 30. HRMS spectra of **TAP1a** (cation, top and PF_6^- counterion, bottom).

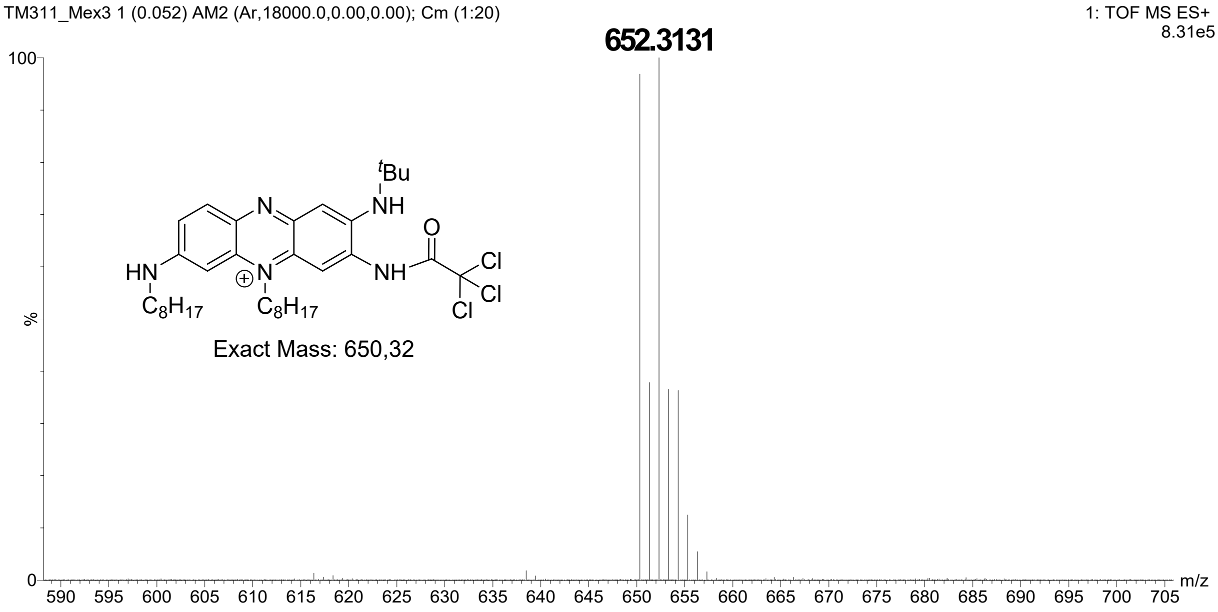

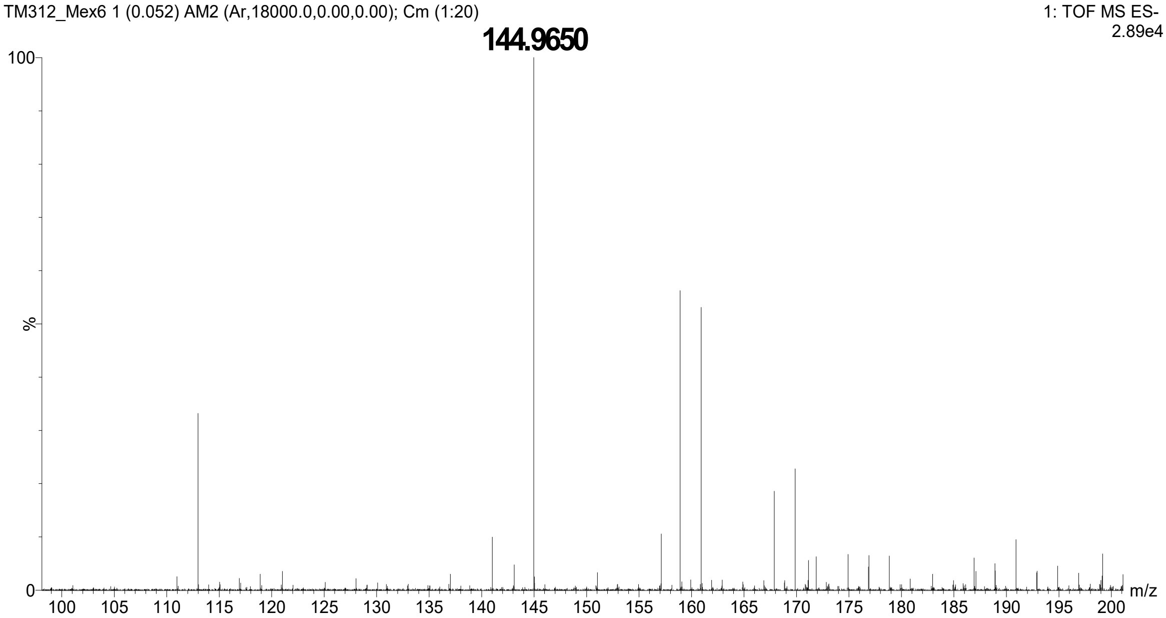

Figure S 31. HRMS spectra of **TAP1b** (cation, top and PF_6^- counterion, bottom).

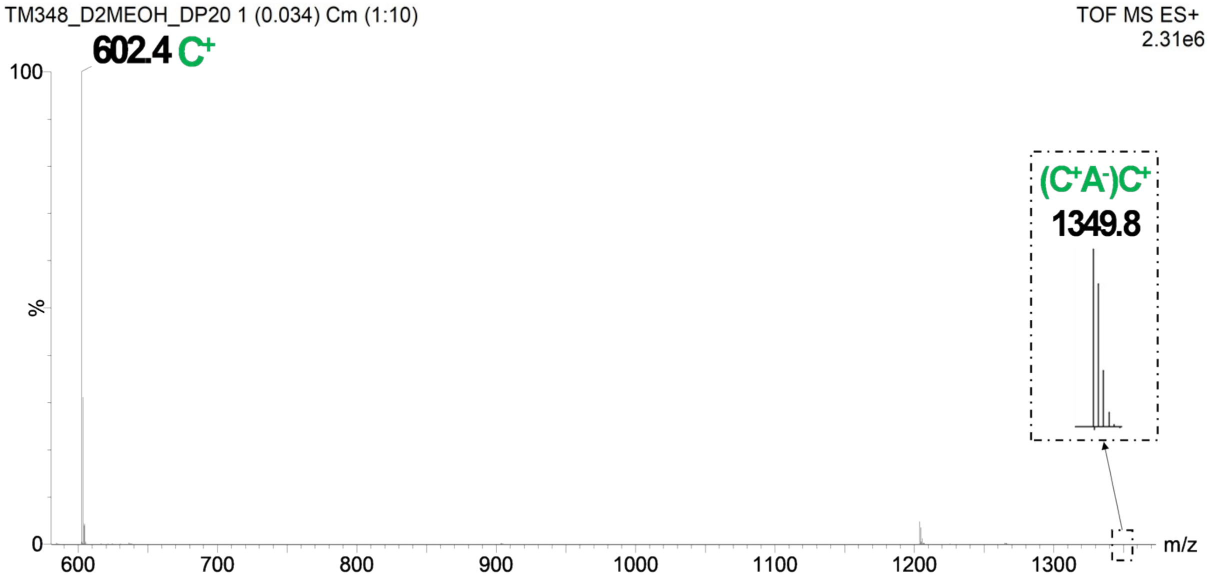

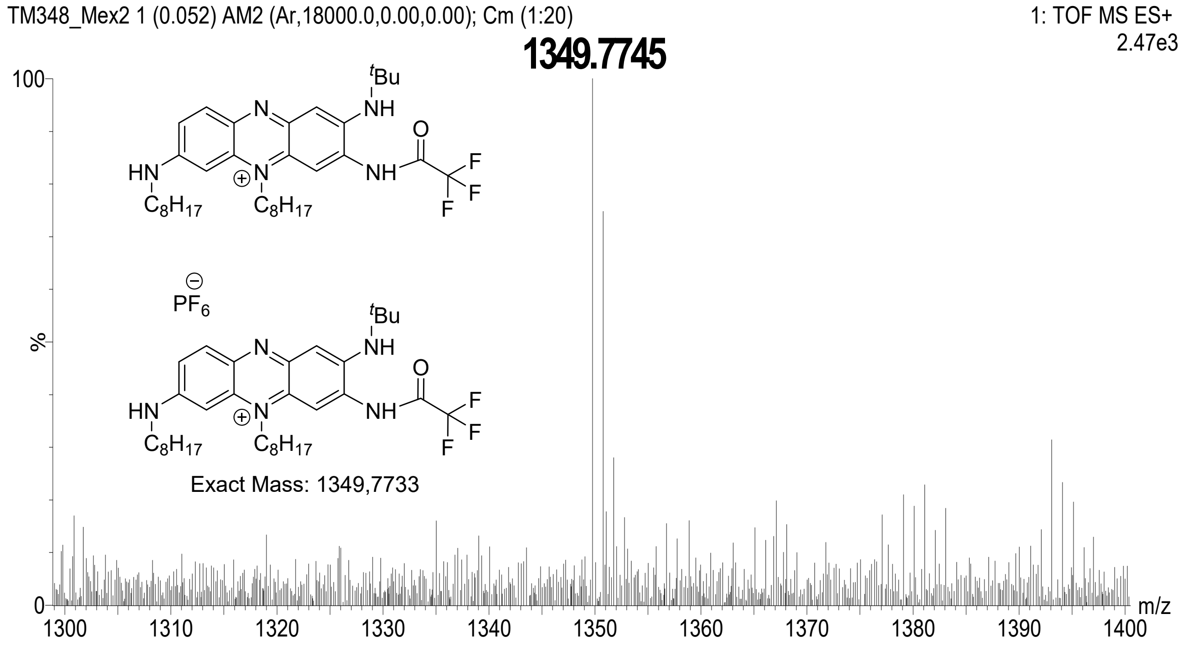

Figure S 32. HRMS spectrum of **TAP1c** detected as an agglomerate of two cations with one counterion.

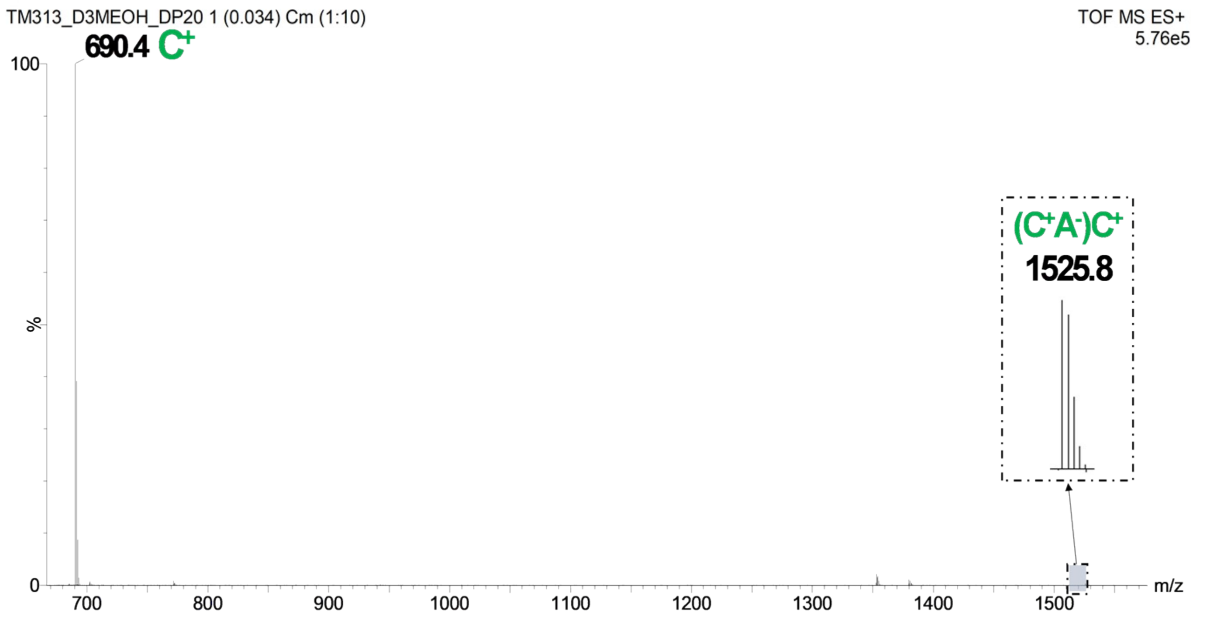

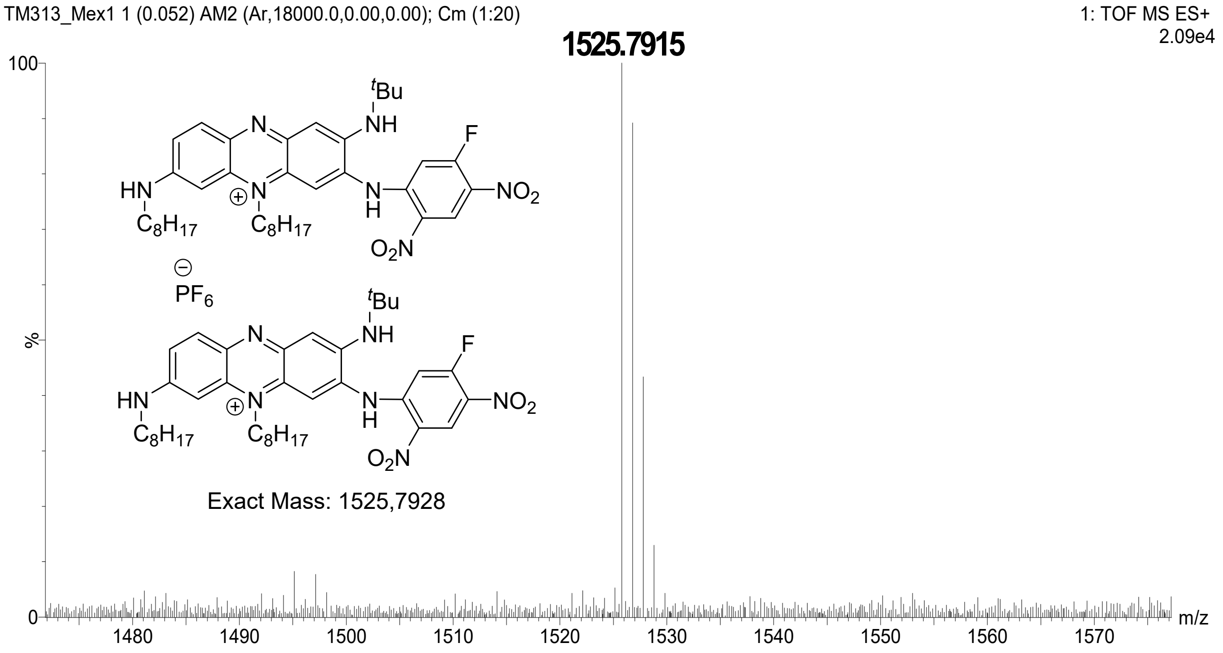

Figure S 33. HRMS spectrum of **TAP2** detected as an agglomerate of two cations with one counterion.

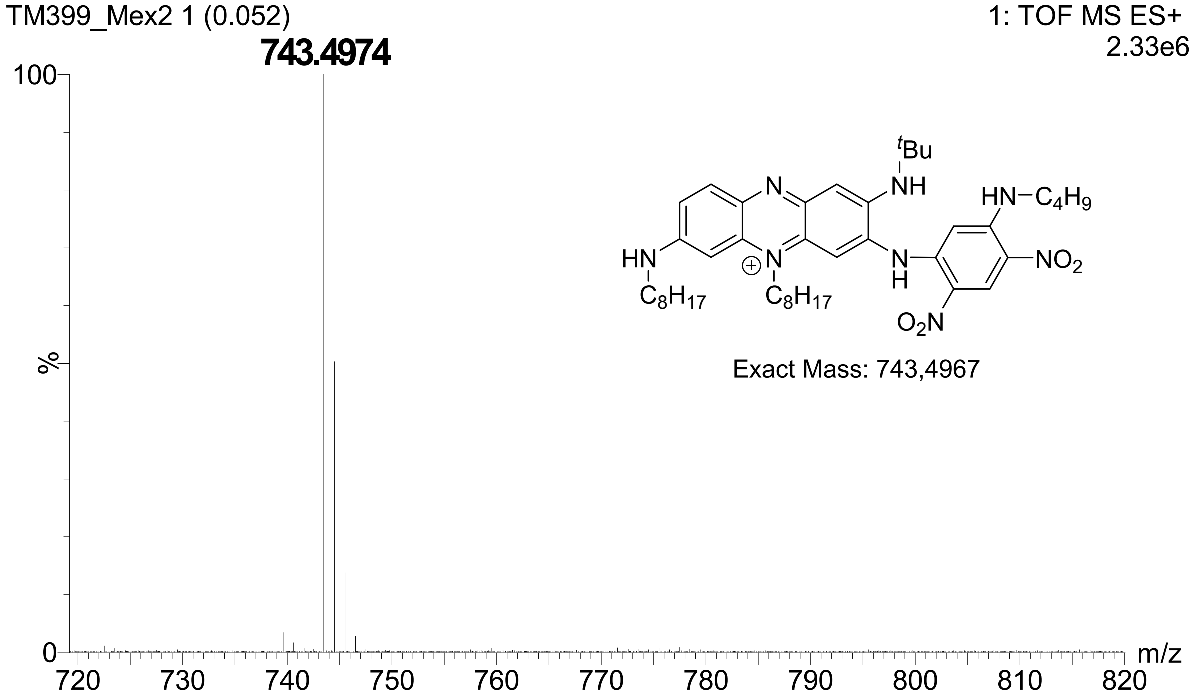

Figure S 34. HRMS spectrum of **TAP3a**.

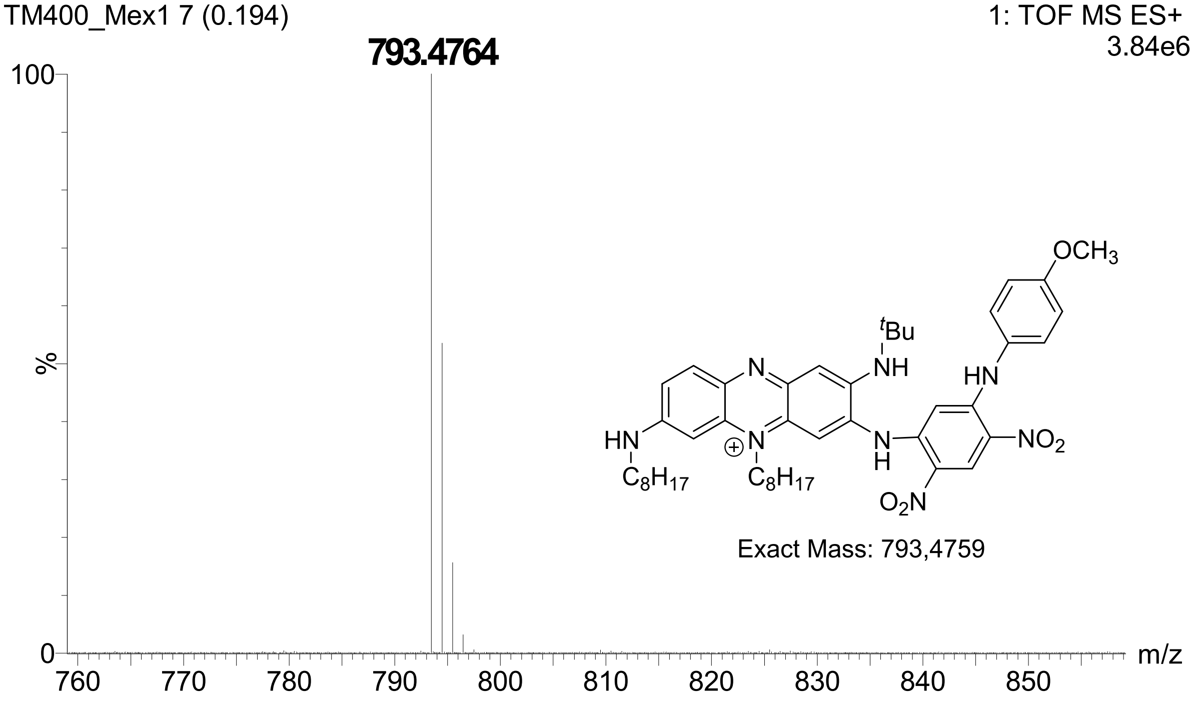

Figure S 35. HRMS spectrum of **TAP3b**.

VI. X-RAY DIFFRACTION

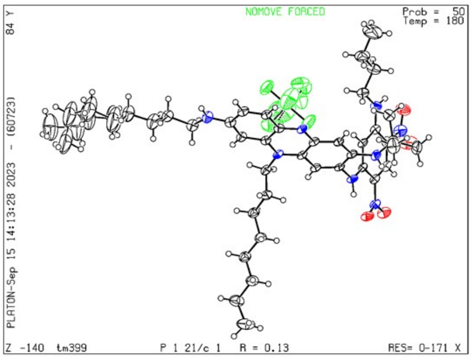

Table S 1. Crystal data and structure refinement for **TAP3a**.

Identification code	tm399
Empirical formula	$C_{42}H_{63}F_6N_8O_4P$
Formula weight	888.97
Temperature/K	180.00(10)
Crystal system	monoclinic
Space group	$P2_1/c$
a/Å	13.7061(5)
b/Å	35.5623(12)
c/Å	9.8813(3)
α/°	90
β/°	106.416(4)
γ/°	90
Volume/Å^3	4620.0(3)
Z	4
ρ_{calc}g/cm^3	1.278
μ/mm^{-1}	1.150
F(000)	1888.0
Crystal size/mm^3	0.18 × 0.14 × 0.04
Radiation	Cu Kα (λ = 1.54184)
2Θ range for data collection/°	6.724 to 139.924
Index ranges	-16 ≤ h ≤ 15, -43 ≤ k ≤ 42, -12 ≤ l ≤ 11
Reflections collected	20778
Independent reflections	8558 [R_{int} = 0.0744, R_{sigma} = 0.0563]
Data/restraints/parameters	8558/102/627
Goodness-of-fit on F^2	1.124
Final R indexes [I>=2σ (I)]	R_1 = 0.1313, wR_2 = 0.2814
Final R indexes [all data]	R_1 = 0.1501, wR_2 = 0.2901
Largest diff. peak/hole / e Å^{-3}	0.61/-0.60

VII. THEORETICAL CALCULATIONS

Table S 2. Charge transfer parameters for absorption: CT distance (Å), CT charge (e) and CT dipole (D). All values have been obtained with Le Bahers' model. LR(neq)-PCM-TD- M06-2X/6-311+G(2d,p) results.

	d^{CT}	q^{CT}	μ^{CT}
**TAP**	0.09	0.47	+0.1
**TAP1b**	1.60	0.54	-3.9
**TAP2**	1.20	0.52	-2.7
**TAP3a**	1.33	0.53	-3.1
**TAP3b**	1.19	0.54	-2.8

Table S 3. Theoretical best estimates for vertical absorption (nm), vertical emission (nm) and 0-0 energies (eV) for all compounds. These values are obtained with an approach based on CC2 and including solvent effects obtained by TD-DFT, see computational details.

	λ^{abs}	λ^{fluo}	ΔE^{00}
**TAP**	503	640	630
**TAP1b**	589	719	717
**TAP2**	575	705	706
**TAP3a**	583	706	707
**TAP3b**	585	723	856

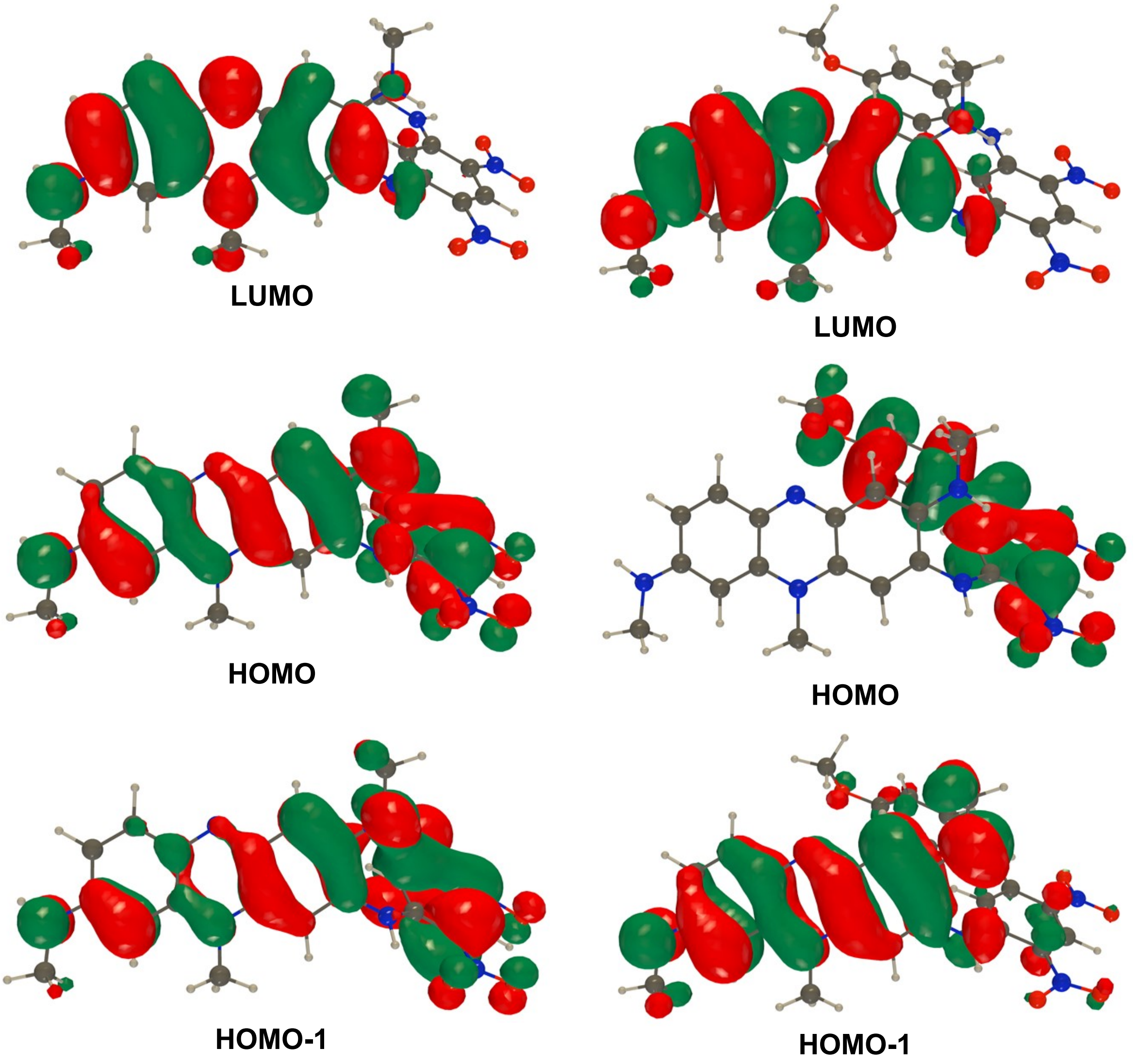

Figure S 36. Frontier MOs of **TAP3a** (left) and **TAP3b** (right), as obtained at the TD-M06-2X/6-311+G(2d,p) level (no solvent effects). At that level of theory, the lowest excited-state is dominated by a H-L transition for **TAP3a** (494 nm, *f=0.35)* but by a H-1 – L transition in **TAP3b** (497 nm, *f=0.29).*

Cartesian coordinates.

Below are the XYZ coordinates in Å for structures. All structures are stable minima (no imaginary frequency).

TAP (S_0)

C	3.4715770	-1.8910800	-0.0358480
C	2.1641520	-2.2294270	0.0013260
C	1.1419030	-1.2297270	0.0394610
C	1.5388880	0.1469490	0.0673440
C	2.8923450	0.4957730	0.0092480
C	3.8658190	-0.5066090	-0.0473650
C	-0.7781280	0.7137590	0.0634310
C	-1.0814410	-0.6864400	0.0331610
C	-2.4430030	-1.1035670	0.0176680
H	-2.6215600	-2.1694330	0.0495010
C	-3.4650190	-0.1993820	-0.0174080
C	-3.1374690	1.2129650	-0.1060400
C	-1.8217880	1.6432240	-0.0228160
H	4.2435560	-2.6509130	-0.0666100
H	1.8406550	-3.2625160	-0.0033470
H	3.1970470	1.5287800	-0.0331070
H	-1.6275840	2.7016360	-0.1118670
N	0.5376810	1.0795850	0.1534580
N	-0.1283460	-1.6143950	0.0438970
C	0.8885920	2.4922970	0.3350310
H	1.0632060	2.9698240	-0.6303310
H	0.0846070	2.9933260	0.8630830
H	1.7806000	2.5555980	0.9509940
N	-4.8066770	-0.5313980	-0.0528020
N	-4.1677010	2.0827500	-0.2413090
N	5.1751310	-0.2186070	-0.1185600
H	-5.0120880	1.7423930	-0.6794770
H	-3.9571560	3.0540670	-0.4151130
H	-5.3798460	0.0773090	0.5197810
H	5.8227300	-0.9884800	-0.1777910
C	-5.1806200	-1.9327120	0.0502570
H	-6.2659530	-2.0029690	0.0362730
H	-4.7894350	-2.4784450	-0.8099780
H	-4.8025220	-2.4037600	0.9653380
C	5.7086480	1.1281360	-0.1399520
H	5.3499110	1.6788890	-1.0140000
H	5.4234830	1.6738380	0.7636950
H	6.7930020	1.0703510	-0.1848290

TAP (S_1)

C	3.5166010	-1.8837900	-0.0617640
C	2.1975600	-2.2314060	-0.0021590
C	1.1542110	-1.2648950	0.0602080
C	1.5353300	0.1171970	0.0924750
C	2.8664410	0.4808740	0.0036080
C	3.8830530	-0.5111160	-0.0719640
C	-0.7861100	0.6844660	0.0607700
C	-1.0749810	-0.7236100	0.0361490
C	-2.4095690	-1.1195580	-0.0155500
H	-2.6144140	-2.1810070	0.0172460
C	-3.4586680	-0.1814440	-0.0584080
C	-3.1480150	1.2234520	-0.1590040
C	-1.8300980	1.6192580	-0.0775750
H	4.2914130	-2.6393970	-0.1087120
H	1.8932760	-3.2709000	-0.0052560
H	3.1529640	1.5207690	-0.0273240
H	-1.6086010	2.6737140	-0.1701020

N	0.5240930	1.0701460	0.2088560
N	-0.1191560	-1.6832960	0.0757310
C	0.8576790	2.4702310	0.4449940
H	1.0065020	3.0096000	-0.4943730
H	0.0552210	2.9354200	1.0115000
H	1.7600350	2.5266900	1.0479320
N	-4.7456050	-0.5487010	-0.0037360
N	-4.2138740	2.1105710	-0.2591290
N	5.1761620	-0.1598750	-0.1494120
H	-4.8492410	1.9210010	-1.0276470
H	-3.9401830	3.0843070	-0.2668180
H	-5.4204170	0.1885850	0.1548430
H	5.8553080	-0.9050390	-0.1984750
C	-5.2021070	-1.9221750	0.0981560
H	-6.2880500	-1.9251300	0.0719300
H	-4.8246850	-2.5084920	-0.7411720
H	-4.8644860	-2.3787220	1.0329090
C	5.6690570	1.2036910	-0.1654600
H	5.2880600	1.7494910	-1.0328530
H	5.3797750	1.7353250	0.7450320
H	6.7538900	1.1748160	-0.2216840

TAP1b (S_0)

C	-6.1622100	1.0444650	-0.4246280
C	-5.0459510	1.7909300	-0.3712690
C	-3.7597510	1.1889460	-0.1227110
C	-3.7080240	-0.2434290	0.0948330
C	-4.8726780	-1.0117770	0.0227430
C	-6.0984800	-0.3912460	-0.2366090
C	-1.3534870	0.0003670	0.3289550
C	-1.4885260	1.3943480	0.0978070
C	-0.3565190	2.2318760	0.0854380
H	-0.5331570	3.2885520	-0.0582780
C	0.9173040	1.7236610	0.2682870
C	1.0346460	0.3019510	0.4213800
C	-0.0539310	-0.5282510	0.4658900
H	-7.1273980	1.4982220	-0.6156900
H	-5.0617340	2.8629640	-0.5184140
H	-4.8365020	-2.0832630	0.1300530
H	0.1263630	-1.5897790	0.5506570
N	-2.4895020	-0.7742490	0.3760780
N	-2.7033980	1.9594450	-0.1020460
C	-2.4055030	-2.1980990	0.7255130
H	-2.4168260	-2.8085830	-0.1782570
H	-1.5019870	-2.3808970	1.2932890
H	-3.2523230	-2.4489210	1.3590230
N	2.0340330	2.4978940	0.2845850
N	2.3348540	-0.2556170	0.5514280
N	-7.2364910	-1.0833540	-0.3248160
H	2.5198000	-0.8558500	1.3445540
H	2.8573030	2.0930610	0.7035410
H	-8.0848810	-0.5742270	-0.5223120
C	1.9483590	3.9440490	0.2599780
H	2.9543140	4.3508150	0.3270460
H	1.5048530	4.2806000	-0.6797660
H	1.3488630	4.3348460	1.0900690
C	-7.3289320	-2.5211930	-0.1534380
H	-6.7253790	-3.0402660	-0.9022220
H	-6.9906970	-2.8131890	0.8440810
H	-8.3672730	-2.8171440	-0.2729030
C	3.2002110	-0.2449100	-0.5014470
O	2.9738790	0.2410250	-1.5729510
C	4.6049130	-0.8464320	-0.1709380
Cl	5.5235900	-1.0652920	-1.6540820
Cl	5.4319400	0.3281160	0.8901170
Cl	4.4439120	-2.4066770	0.6730130

TAP1b (S_1)

C	6.1952440	0.9890680	0.5059830
C	5.0724960	1.7590030	0.4113790
C	3.7954310	1.2117340	0.1063860
C	3.7216810	-0.1982300	-0.1383760
C	4.8485050	-0.9932230	-0.0208250
C	6.1076390	-0.4152290	0.3029290
C	1.3622930	0.0421660	-0.3795360
C	1.5270920	1.4478860	-0.1318050
C	0.3923710	2.2544110	-0.1169730
H	0.5417180	3.3153290	0.0291580
C	-0.9039730	1.7278290	-0.3070180
C	-1.0507900	0.3074810	-0.4493670
C	0.0606270	-0.4964970	-0.4902770
H	7.1554410	1.4305100	0.7426030
H	5.1136760	2.8288870	0.5742430
H	4.7869640	-2.0624790	-0.1513460
H	-0.0877190	-1.5646660	-0.5692730
N	2.4839690	-0.7344740	-0.4851080
N	2.7314980	2.0264130	0.0720360
C	2.3831150	-2.1222800	-0.9315950
H	2.2388910	-2.7988320	-0.0857350
H	1.5513730	-2.2138240	-1.6247440
H	3.2879800	-2.3897040	-1.4697890
N	-1.9748550	2.5215190	-0.3631510
N	-2.3490030	-0.2495670	-0.5603690
N	7.2011650	-1.1815690	0.4141610
H	-2.5420940	-0.8593120	-1.3449620
H	-2.8655550	2.0898540	-0.5696810
H	8.0690740	-0.7188690	0.6422810
C	-1.9452900	3.9653340	-0.2096230
H	-2.9626170	4.3363590	-0.2872410
H	-1.5408170	4.2348000	0.7678510
H	-1.3355420	4.4238750	-0.9913430
C	7.2330790	-2.6197200	0.2249740
H	6.5768540	-3.1231440	0.9394890
H	6.9297630	-2.8877000	-0.7906720
H	8.2510750	-2.9630500	0.3863460
C	-3.2147710	-0.2165980	0.4915030
O	-3.0042580	0.3399510	1.5338980
C	-4.6042680	-0.8668040	0.1973670
Cl	-5.4795330	-1.1048720	1.7038890
Cl	-5.4880790	0.2847290	-0.8452570
Cl	-4.4245470	-2.4229080	-0.6469920

TAP2 (S_0)

C	6.6588510	-1.3022610	-0.3204010
C	5.5603780	-1.8656400	0.2098870
C	4.3254600	-1.1294820	0.3163700
C	4.3098560	0.2475960	-0.1392200
C	5.4523170	0.8176810	-0.7044050
C	6.6257830	0.0626740	-0.8067300
C	2.0132630	0.2977710	0.4802320
C	2.1111740	-1.0553180	0.8958810
C	0.9872840	-1.7264800	1.4171640
H	1.1398230	-2.7407460	1.7585700
C	-0.2420730	-1.1025030	1.5140020
C	-0.3406240	0.2365970	1.0046690
C	0.7494820	0.9180010	0.5234360
H	7.5862600	-1.8562990	-0.4022090
H	5.5520520	-2.8859520	0.5703660
H	5.4349450	1.8218300	-1.0953000
H	0.5873000	1.9138790	0.1387150
N	3.1469590	0.9314600	0.0247770
N	3.2816370	-1.7326620	0.8222980
C	3.1206080	2.3640860	-0.2968390
H	3.0060650	2.5064250	-1.3721550

H	2.3078130	2.8420830	0.2356710
H	4.0505170	2.8121330	0.0434770
N	-1.3543840	-1.7076990	2.0213510
N	-1.5899900	0.8996590	1.0546400
N	7.7394730	0.5560990	-1.3502290
H	-1.6284310	1.8147800	1.4864950
H	-2.0735640	-1.0832530	2.3569440
H	8.5465650	-0.0475380	-1.4015640
C	-1.2635850	-2.9997970	2.6751890
H	-2.2489870	-3.2668570	3.0491570
H	-0.9512180	-3.7620570	1.9584880
H	-0.5538700	-2.9911490	3.5101640
C	7.8617370	1.9027770	-1.8769710
H	7.1615770	2.0611990	-2.7008970
H	7.6675600	2.6410510	-1.0949620
H	8.8746870	2.0384120	-2.2451740
C	-2.6780390	0.5402900	0.3182020
C	-2.6127390	-0.5634610	-0.5529920
C	-3.9272380	1.2142940	0.3924050
C	-3.6990180	-0.9605930	-1.2908670
H	-1.6936310	-1.1211150	-0.6690440
C	-5.0185080	0.8019890	-0.3515460
C	-4.9238350	-0.2823810	-1.1955100
H	-5.9556210	1.3339570	-0.2682520
F	-3.5532640	-2.0109210	-2.0788200
N	-6.1005840	-0.6609510	-1.9719420
O	-5.9454810	-1.4150090	-2.9063120
O	-7.1645530	-0.1854410	-1.6348030
N	-4.1325860	2.3888420	1.2347500
O	-5.2574000	2.8092530	1.3677340
O	-3.1559870	2.8973520	1.7645850

TAP2 (S_1)

C	6.6455050	1.1871680	0.5918210
C	5.5612290	1.8259290	0.0651400
C	4.3371990	1.1535120	-0.2086320
C	4.2800870	-0.2530230	0.0588400
C	5.3639750	-0.9070040	0.6182060
C	6.5692260	-0.2005160	0.8926470
C	1.9909680	-0.2126780	-0.6061550
C	2.1365090	1.1974790	-0.8531520
C	1.0274540	1.9107580	-1.3002120
H	1.1709220	2.9589990	-1.5236190
C	-0.2278520	1.2957230	-1.4912270
C	-0.3758830	-0.0892960	-1.1403440
C	0.7166520	-0.8108330	-0.7227730
H	7.5652160	1.7225100	0.7931710
H	5.5923080	2.8844530	-0.1616030
H	5.3085920	-1.9538610	0.8734830
H	0.5628950	-1.8461090	-0.4508630
N	3.1020810	-0.9272500	-0.2514010
N	3.3035630	1.8585400	-0.6883120
C	3.0467970	-2.3859810	-0.1755230
H	2.7459590	-2.7164600	0.8215310
H	2.3420930	-2.7536190	-0.9161000
H	4.0223540	-2.7934140	-0.4234060
N	-1.2721800	1.9689060	-1.9822740
N	-1.6417250	-0.7066830	-1.2712980
N	7.6217310	-0.8332270	1.4255510
H	-1.7409040	-1.4918720	-1.9034260
H	-2.1101210	1.4386090	-2.1832840
H	8.4530660	-0.2855320	1.5947800
C	-1.2463780	3.3701730	-2.3639990
H	-2.2409290	3.6501790	-2.6978310
H	-0.9686790	3.9905600	-1.5101600
H	-0.5347390	3.5365870	-3.1761630
C	7.6652900	-2.2440970	1.7628670

H	6.9111380	-2.4909260	2.5144630
H	7.5035190	-2.8625060	0.8761870
H	8.6477810	-2.4672170	2.1694250
C	-2.6807520	-0.4796880	-0.4205710
C	-2.5338210	0.4371250	0.6390660
C	-3.9570900	-1.0927480	-0.5527520
C	-3.5666610	0.7152280	1.4984650
H	-1.5865090	0.9301790	0.8127650
C	-4.9925090	-0.8020770	0.3174040
C	-4.8187660	0.0992170	1.3449220
H	-5.9509580	-1.2846280	0.1880840
F	-3.3414620	1.5898790	2.4617630
N	-5.9425900	0.3531900	2.2410100
O	-5.7224170	0.9448090	3.2741240
O	-7.0317390	-0.0543820	1.8951850
N	-4.2479580	-2.0736380	-1.5934740
O	-5.3844060	-2.4674960	-1.7095250
O	-3.3273590	-2.4529230	-2.3018030

TAP3a (S_0)

C	6.7408340	-1.3176130	-0.0672340
C	5.6369430	-1.7670610	0.5547400
C	4.4089420	-1.0141800	0.5259700
C	4.4030670	0.2548610	-0.1721390
C	5.5502510	0.7044480	-0.8310510
C	6.7188690	-0.0625670	-0.7902360
C	2.1081120	0.4388730	0.4294980
C	2.1961950	-0.8183900	1.0839510
C	1.0665890	-1.3709270	1.7208180
H	1.2112320	-2.3067680	2.2423700
C	-0.1551040	-0.7248020	1.7110510
C	-0.2468640	0.5052350	0.9685880
C	0.8509850	1.0711630	0.3672820
H	7.6631270	-1.8860680	-0.0464990
H	5.6205800	-2.7056980	1.0931760
H	5.5402880	1.6202350	-1.3991860
H	0.6980460	1.9834640	-0.1900320
N	3.2446610	0.9662520	-0.1375820
N	3.3587130	-1.5075860	1.1324890
C	3.2279860	2.3130500	-0.7227070
H	3.1085680	2.2507350	-1.8050550
H	2.4216300	2.8897660	-0.2874690
H	4.1630640	2.8090430	-0.4763720
N	-1.2604570	-1.2118750	2.3383220
N	-1.4784520	1.1905110	0.9142920
N	7.8383170	0.3217570	-1.4081300
H	-1.4701340	2.1855060	1.1020290
H	-1.9979450	-0.5455190	2.5132520
H	8.6444270	-0.2813980	-1.3438220
C	-1.1772450	-2.3539750	3.2269720
H	-2.1584510	-2.5248490	3.6627630
H	-0.8904370	-3.2481830	2.6686580
H	-0.4508270	-2.1964860	4.0322860
C	7.9679280	1.5543710	-2.1621520
H	7.2689970	1.5695660	-3.0019460
H	7.7768080	2.4197200	-1.5224720
H	8.9815270	1.6185290	-2.5475680
C	-2.5951260	0.7260570	0.2652130
C	-2.5858390	-0.5173720	-0.3527480
C	-3.8124480	1.4753180	0.2232280
C	-3.7098430	-1.0887650	-0.9824860
H	-1.6636420	-1.0766620	-0.3512370
C	-4.9233480	0.9545800	-0.4086980
C	-4.9028310	-0.2924830	-0.9959050
H	-5.8333270	1.5358870	-0.4393020
N	-6.1306600	-0.7314360	-1.6241900
O	-6.1447560	-1.8263330	-2.1723810

O	-7.0997700	-0.0025880	-1.5851160
N	-3.9496850	2.7987190	0.7889360
O	-5.0507240	3.3050690	0.8256730
O	-2.9454600	3.3638910	1.2081800
N	-3.6200090	-2.3096090	-1.5211530
H	-4.4430380	-2.6736660	-1.9753810
C	-2.4222510	-3.1257570	-1.4565340
H	-1.5934060	-2.6600050	-1.9960890
H	-2.1204930	-3.2941460	-0.4189960
H	-2.6427230	-4.0848410	-1.9173760

TAP3a (*S_1*)

C	6.7079360	-1.3294120	0.0197950
C	5.6146540	-1.6502190	0.7707200
C	4.4059990	-0.9010320	0.7189680
C	4.3760000	0.2475940	-0.1371170
C	5.4690220	0.5682880	-0.9226230
C	6.6568670	-0.2129410	-0.8592520
C	2.0875710	0.5458680	0.4631980
C	2.2077260	-0.6136630	1.3059210
C	1.0854920	-1.0358160	2.0141590
H	1.2083260	-1.8802590	2.6785170
C	-0.1566430	-0.3747360	1.9071280
C	-0.2845240	0.7117640	0.9731500
C	0.8239740	1.1522990	0.2895770
H	7.6152090	-1.9183470	0.0757010
H	5.6264910	-2.5031320	1.4380420
H	5.4312070	1.3992540	-1.6097400
H	0.6901550	1.9588180	-0.4182320
N	3.2136810	1.0144970	-0.1577090
N	3.3607250	-1.3031620	1.4553510
C	3.1926220	2.2968960	-0.8581270
H	2.9147270	2.1689820	-1.9070580
H	2.4859660	2.9596980	-0.3664700
H	4.1742500	2.7567250	-0.7880960
N	-1.2078070	-0.7347580	2.6470630
N	-1.5338700	1.3453180	0.8127890
N	7.7168500	0.0980800	-1.6169270
H	-1.5903750	2.3473980	0.9477880
H	-2.0421630	-0.1680300	2.5651950
H	8.5360780	-0.4856470	-1.5293330
C	-1.2083690	-1.8162340	3.6158940
H	-2.1971570	-1.8740320	4.0608880
H	-0.9805670	-2.7659650	3.1274860
H	-0.4717400	-1.6312500	4.4010660
C	7.7796790	1.2119460	-2.5448970
H	7.0172750	1.1172180	-3.3224250
H	7.6433860	2.1631350	-2.0237040
H	8.7588710	1.2096180	-3.0155810
C	-2.6027740	0.7651240	0.1793900
C	-2.5067790	-0.5348570	-0.3074460
C	-3.8589290	1.4325320	0.0344620
C	-3.5737430	-1.2256020	-0.9160730
H	-1.5505410	-1.0306970	-0.2379370
C	-4.9197230	0.7860210	-0.5674540
C	-4.8125930	-0.5086860	-1.0273190
H	-5.8601040	1.3065820	-0.6759390
N	-5.9957060	-1.0771940	-1.6372060
O	-5.9362700	-2.2223780	-2.0670420
O	-7.0025860	-0.4031450	-1.6982120
N	-4.0862610	2.7941730	0.4621540
O	-5.2107120	3.2440010	0.4019550
O	-3.1313360	3.4466580	0.8693860
N	-3.3966710	-2.4779650	-1.3501460
H	-4.1852950	-2.9281280	-1.7884450
C	-2.1474810	-3.2034550	-1.2187460
H	-1.3428480	-2.7127060	-1.7723950

H	-1.8534560	-3.2932320	-0.1692400
H	-2.2929630	-4.2000950	-1.6262040

TAP3b (S_0)

C	-6.5358360	0.4210470	0.1910150
C	-5.4497920	0.7007170	0.9326300
C	-4.2255710	-0.0393730	0.7646930
C	-4.2032000	-1.1094990	-0.2111130
C	-5.3317380	-1.3814870	-0.9882590
C	-6.4982560	-0.6323070	-0.8028690
C	-1.9231320	-1.4426950	0.3815480
C	-2.0297120	-0.3811600	1.3183560
C	-0.9136200	0.0035670	2.0886570
H	-1.0736460	0.7781620	2.8261350
C	0.3189330	-0.5938250	1.9159460
C	0.4320790	-1.6005050	0.8916160
C	-0.6568420	-2.0259750	0.1721270
H	-7.4548860	0.9810360	0.3164210
H	-5.4473520	1.4880790	1.6754730
H	-5.3066830	-2.1382940	-1.7552770
H	-0.4904230	-2.7618960	-0.6007220
N	-3.0475930	-1.8192350	-0.3139160
N	-3.1927400	0.2830000	1.5023550
C	-3.0187380	-2.9964750	-1.1913370
H	-2.8780550	-2.6897430	-2.2284570
H	-2.2205640	-3.6601250	-0.8823240
H	-3.9586640	-3.5314650	-1.0827890
N	1.4244170	-0.2403350	2.6313540
N	1.6996590	-2.1700110	0.6404160
N	-7.6000670	-0.8497240	-1.5246600
H	1.7822960	-3.1772100	0.5914510
H	2.1746040	-0.9163020	2.6334230
H	-8.4048730	-0.2681280	-1.3460290
C	1.3148920	0.6139760	3.7981060
H	2.2969100	0.6993870	4.2570070
H	0.9869670	1.6161000	3.5088500
H	0.6078220	0.2171990	4.5352420
C	-7.7108600	-1.8690710	-2.5510770
H	-7.0000490	-1.6835950	-3.3601840
H	-7.5235110	-2.8603290	-2.1308220
H	-8.7185500	-1.8434460	-2.9559200
C	2.7272110	-1.4458680	0.0845850
C	2.5146440	-0.1217910	-0.2665060
C	4.0370710	-1.9755750	-0.1165800
C	3.5176110	0.7399520	-0.7398780
H	1.5167550	0.2753660	-0.1699620
C	5.0363080	-1.1708850	-0.6235880
C	4.8115770	0.1592100	-0.9225330
H	6.0207520	-1.5872320	-0.7815500
N	5.9361200	0.9050830	-1.4485250
O	5.7461070	2.0564370	-1.8184100
O	7.0200800	0.3643620	-1.5029120
N	4.3747820	-3.3561970	0.1510260
O	5.5368740	-3.6948720	0.0860430
O	3.4676140	-4.1306440	0.4296200
N	3.2264850	2.0348110	-0.9895880
H	3.9406800	2.5875960	-1.4430580
C	1.9532270	2.6174070	-0.7252400
C	1.4405990	2.6341840	0.5671320
C	1.2034100	3.1563460	-1.7707040
C	0.1671830	3.1348500	0.8179630
H	2.0317600	2.2233150	1.3789310
C	-0.0542990	3.6793270	-1.5253180
H	1.6056950	3.1474760	-2.7768070
C	-0.5922520	3.6501110	-0.2346550
H	-0.2153230	3.1246760	1.8292070
H	-0.6533020	4.0958330	-2.3254020

O	-1.8474190	4.1376580	-0.1019390
C	-2.4677870	4.0249220	1.1705630
H	-1.9322870	4.6096980	1.9232840
H	-3.4708900	4.4260560	1.0496600
H	-2.5273240	2.9777690	1.4840790

TAP3b (*S_1*)

C	6.0346210	0.3824270	0.0122360
C	5.1365100	1.2757020	0.5188430
C	3.7929510	1.3502680	0.0591020
C	3.4194220	0.5004620	-1.0320460
C	4.3130240	-0.4268430	-1.5379460
C	5.6320050	-0.5162420	-1.0134360
C	1.2070480	1.3659980	-0.8533050
C	1.6554350	2.1585430	0.2598350
C	0.7235350	2.9464150	0.9303360
H	1.0861070	3.5563020	1.7466460
C	-0.6496130	2.9252540	0.5966910
C	-1.0823430	2.1106930	-0.5026590
C	-0.1597320	1.3714810	-1.2038770
H	7.0491860	0.3365380	0.3888230
H	5.4137730	1.9541130	1.3169040
H	4.0189320	-1.1134160	-2.3164790
H	-0.5302450	0.7267270	-1.9894890
N	2.1305420	0.6249140	-1.5394920
N	2.9410700	2.1782440	0.6799340
C	1.7418920	-0.0810050	-2.7570890
H	1.3043930	-1.0550210	-2.5195840
H	1.0234370	0.5224980	-3.3058300
H	2.6121340	-0.2085940	-3.3929640
N	-1.5543770	3.6096750	1.2993160
N	-2.4716260	1.9488850	-0.7292560
N	6.4919130	-1.4284400	-1.4851070
H	-2.9973180	2.6347430	-1.2571680
H	-2.5224830	3.4949800	1.0265260
H	7.4167900	-1.4457840	-1.0805160
C	-1.2554880	4.4511720	2.4436630
H	-2.1845250	4.8879450	2.7975840
H	-0.8079810	3.8623960	3.2477840
H	-0.5695630	5.2523630	2.1611610
C	6.1886720	-2.4047640	-2.5146770
H	5.3729870	-3.0621580	-2.2019010
H	5.9129820	-1.9132430	-3.4512820
H	7.0759260	-3.0078430	-2.6863660
C	-3.0873930	0.7495210	-0.4811640
C	-2.3712670	-0.2616990	0.1495630
C	-4.4437310	0.4624140	-0.8228670
C	-2.8714410	-1.5422080	0.4170090
H	-1.3529310	-0.0634780	0.4347100
C	-4.9815930	-0.7791160	-0.5408860
C	-4.2376350	-1.7734300	0.0624700
H	-6.0097590	-0.9795340	-0.8060270
N	-4.8980050	-3.0408770	0.2900190
O	-4.2513370	-3.9442930	0.8034760
O	-6.0610000	-3.1601290	-0.0324160
N	-5.3102020	1.4237190	-1.4665700
O	-6.4437430	1.0960920	-1.7438650
O	-4.8618250	2.5395190	-1.7047520
N	-2.0502880	-2.4594830	0.9736060
H	-2.4181390	-3.3864770	1.1330080
C	-0.6612760	-2.1823410	1.1813390
C	-0.2426460	-1.5338790	2.3346000
C	0.2696430	-2.4894240	0.1865750
C	1.0945850	-1.1867340	2.5110320
H	-0.9720270	-1.2841320	3.0964270
C	1.6048970	-2.1644970	0.3587090
H	-0.0624680	-2.9872710	-0.7174990

C	2.0280780	-1.5123090	1.5241540
H	1.3921460	-0.6791500	3.4176930
H	2.3488190	-2.4191980	-0.3875960
O	3.3524000	-1.2530390	1.6124970
C	3.8190880	-0.6116620	2.7916220
H	3.5630120	-1.1940840	3.6802450
H	4.8995030	-0.5546470	2.6917050
H	3.4046790	0.3985830	2.8698400

VIII. REFERENCES

1 O. V. Dolomanov, L. J. Bourhis, R. J. Gildea, J. A. K. Howard and H. Puschmann, *J. Appl. Crystallogr.*, 2009, **42**, 339–341.
2 G. M. Sheldrick, *Acta Crystallogr. Sect. Found. Adv.*, 2015, **71**, 3–8.
3 N. G. Connelly and W. E. Geiger, *Chem. Rev.*, 1996, **96**, 877–910.
4 A. M. Brouwer, 2011, **83**, 2213–2228.
5 M. Frisch, G. Trucks, H. Schlegel, G. Scuseria, M. Robb, J. Cheeseman, G. Scalmani, V. Barone, G. Petersson and H. Nakatsuji, .
6 M. Frisch, G. Trucks, H. Schlegel, G. Scuseria, M. Robb, J. Cheeseman, G. Scalmani, V. Barone, G. Petersson and H. Nakatsuji, *Gaussian09 Revis. D*.
7 Y. Zhao and D. G. Truhlar, *Theor. Chem. Acc.*, 2008, **120**, 215–241.
8 J. Tomasi, B. Mennucci and R. Cammi, *Chem. Rev.*, 2005, **105**, 2999–3094.
9 C. A. Guido, A. Chrayteh, G. Scalmani, B. Mennucci and D. Jacquemin, *J. Chem. Theory Comput.*, 2021, **17**, 5155–5164.
10 A. D. Laurent and D. Jacquemin, *Int. J. Quantum Chem.*, 2013, **113**, 2019–2039.
11 O. Christiansen, H. Koch and P. Jørgensen, *Chem. Phys. Lett.*, 1995, **243**, 409–418.
12 TURBOMOLE V7.3/V7.5, a development of University of Karlsruhe and Forschungszentrum Karlsruhe GmbH, 1989−2007; TURBOMOLE GmbH. http://www.turbomole.com.
13 D. Jacquemin, I. Duchemin and X. Blase, *J. Chem. Theory Comput.*, 2015, **11**, 5340–5359.
14 Z. Chen, S. Pascal, M. Daurat, L. Lichon, C. Nguyen, A. Godefroy, D. Durand, L. M. A. Ali, N. Bettache, M. Gary-Bobo, P. Arnoux, J.-F. Longevial, A. D'Aléo, G. Marchand, D. Jacquemin and O. Siri, *ACS Appl. Mater. Interfaces*, 2021, **13**, 30337–30349.
